# Cobalt-Based Electrocatalysts for Sustainable Nitrate Conversion: Structural Design and Mechanistic Advancements

**DOI:** 10.1007/s40820-025-01877-z

**Published:** 2025-10-01

**Authors:** GuoLiang Chang, Xueqiu Chen, Jing-Jing Lv, Zhijie Kong, Zheng-Jun Wang

**Affiliations:** 1 Henan Key Laboratory of Crystalline Molecular Functional Materials, Green Catalysis Center, and College of Chemistry, Zhengzhou University, Zhengzhou, 450001 People’s Republic of China; 2 School of Mechanical and Electrical Engineering and Intelligent Manufacturing, Henan Open University, Zhengzhou, 450046 People’s Republic of China; 3 Key Laboratory of Biohealth Materials and Chemistry of Wenzhou, College of Chemistry and Materials Engineering, Wenzhou University, Wenzhou, 325035 People’s Republic of China; 4 Key Laboratory of Advanced Energy Materials Chemistry (Ministry of Education), Nankai University, Tianjin, 300071 People’s Republic of China

**Keywords:** Electrocatalytic nitrate reduction reaction, Cobalt-based Electrocatalysts, Electronic structure, Coordination environment

## Abstract

Electrocatalytic nitrate-to-ammonia conversion offers dual environmental and sustainable synthesis benefits, but achieving high efficiency with low-cost catalysts remains a major challenge. This review focuses on cobalt-based electrocatalysts, emphasizing their structural engineering for enhanced the performance of electrocatalytic nitrate reduction reaction (NO_3_RR) through dimensional control, compositional tuning, and coordination microenvironment modulation. Notably, by critically analyzing metallic cobalt, cobalt alloys, cobalt compounds, cobalt single atom and molecular catalyst configurations, we firstly establish correlations between atomic-scale structural features and catalytic performance in a coordination environment perspective for NO_3_RR, including the dynamic reconstruction during operation and its impact on active site. Synergizing experimental breakthroughs with computational modeling, we decode mechanisms underlying competitive hydrogen evolution suppression, intermediate adsorption-energy optimization, and durability enhancement in complex aqueous environments. The development of cobalt-based catalysts was summarized and prospected, and the emerging opportunities of machine learning in accelerating the research and development of high-performance catalysts and the configuration of series reactors for scalable nitrate-to-ammonia systems were also introduced. Bridging surface science and applications, it outlines a framework for designing multifunctional electrocatalysts to restore nitrogen cycle balance sustainably.

## Highlights

- This review covers almost all cobalt-based electrocatalysts for nitrate reduction reaction (NO_3_RR), including metallic cobalt, cobalt alloys, cobalt compounds, cobalt single-atom and molecular catalysts, etc.
- The mechanism of enhancing the NO_3_RR performance by suppressing the hydrogen evolution reaction, as well as the durability and degradation processes, was discussed from the perspective of the electronic structure and adsorption behavior.
- The influence of different coordination environments of Co active sites on NO_3_RR performance was discussed, including different isomorphic forms of the same elements around Co, different types of elements, doping of trace elements, and in situ evolution of constituent elements, etc.

## Graphical abstract

**Figure:**
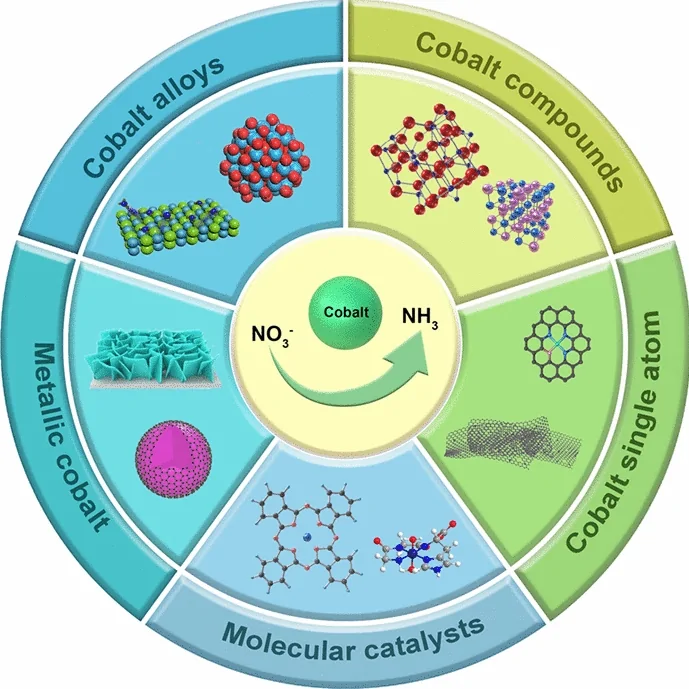

## Introduction

Nitrogen, as a fundamental constituent of the Earth's ecosystem, plays a pivotal role in sustaining life and driving technological advancements through its intricate biogeochemical cycle [1–3]. In the era of carbon neutrality and sustainable development, nitrogen-based compounds have garnered significant attention as promising hydrogen carriers, owing to their exceptional energy density (3 kWh kg^−1^), facile storage characteristics, and efficient transportability [4–8]. However, the widespread use of agricultural fertilizers and uncontrolled discharge of industrial effluents have led to severe nitrate (NO_3_^−^) pollution, disrupting the global nitrogen cycle and posing serious human health risks [9, 10]. Current nitrate remediation strategies, including physical separation (electrodialysis, ion exchange, reverse osmosis) and biological denitrification, face substantial limitations such as high operational costs, low efficiency, stringent reaction conditions, and potential secondary pollution [10]. While biological treatments offer an eco-friendly approach, their practical implementation is hindered by slow reaction kinetics and incomplete nitrate conversion. Moreover, these conventional methods predominantly yield N_2_ as the end-product, which lacks economic value despite being environmentally benign.

Electrocatalytic nitrate reduction reaction (NO_3_RR) has emerged as a transformative technology, offering a sustainable pathway for nitrate conversion with exceptional efficiency, environmental compatibility, and operational flexibility. This approach enables the selective reduction of nitrate to either nitrogen gas or value-added ammonia (NH_3_), leveraging nitrate's favorable physicochemical properties such as high water solubility (9.16 g L^−1^ at 20 °C), relatively low N–O bond energy (204 kJ mol^−1^), and strong chemical polarity [6]. The production of NH_3_ through NO_3_RR is particularly attractive: NH_3_ serves as a crucial industrial chemical and has recently gained recognition as a carbon-free energy carrier for next-generation fuel cells [11, 12]. This electrochemical approach presents a sustainable alternative to the conventional Haber–Bosch process, which accounts for approximately 1.4% of global CO_2_ emissions and operates under energy-intensive conditions (300–500 °C, 150–300 bar) [3]. So far, many kinds of catalysts have been developed to achieve this goal, including precious metals, transition metals, and their oxide/phosphide. For example, metal Pt, Rh, Cu, and metal oxides CuO, Co_3_O_4_ all showed electrochemical activity for NO_3_RR [14, 15]. Although noble metal-based catalysts exhibit relatively decent NO_3_RR performance, their high cost and insufficient supply obstruct their large-scale application [15, 16]. Therefore, the most promising candidates are transition metal-based materials, especially for Cu, Fe, and Co [17–21]. Among them, copper (Cu) is the most studied catalyst, however, Cu-based materials generally require a large overpotential in order to attain satisfactory NH_3_ selectivity and production rates [21,22]. Besides, Cu exhibits hydrogen evolution reaction (HER) inhibition capabilities, this leads to a lack of *H on the electrode surface, which hinders the hydrogenation during NO_3_RR, resulting an insufficient NH_3_ selectivity production rates [22, 23]. Iron (Fe) represents the lowest cost but has an insufficient electrocatalytic activity [24, 25]. Even so, a large effort has been devoted to developing efficient materials, and several review articles have summarized the latest progress on Cu- and Fe-based electrocatalysts for NO_3_RR [22, 25]. Cobalt (Co)-based materials have the merits of high intrinsic activity toward electrocatalytic reaction, in addition, cobalt is earth-abundant and environmentally friendly, and even can be obtained from recycled batteries, thus showing great advantage for practical application [20, 26, 27]. However, the slow reaction kinetics, low product selectivity, and insufficient stability hinder their further application [16, 17]. While several excellent reviews summarize recent NO_3_RR progress, they focus either on some specific catalyst, such as Ti/Fe-based materials, or an overview involving multiple types of catalyst, neglecting Co-based catalysts [25, 31–33]. Wang's group recently categorized advances in Co-based NO_3_RR electrocatalysts into five types: oxides/hydroxides, alloys, metals, heteroatom-doped materials, and MOFs/derivatives [9 ]. Supplementing this, our review comprehensively analyzes the latest progress using a novel taxonomy focused on the Co active site's coordination environment (O, metal, P, B, S) and its correlation with NO_3_RR performance. We also further elucidate reaction mechanisms, including HER competition, work function relationships, and durability/degradation processes In addition, special attention is given to molecular catalysts such as cobalt phthalocyanine (CoPc), with particular emphasis on the effects of dimensional architecture, compositional variation, and coordination environment on catalytic performance. The review systematically discusses the mechanistic aspects of these materials, establishes fundamental design principles for catalyst development, and concludes with a critical analysis of current challenges and future perspectives. This work aims to provide a scientific foundation for the rational design of high-performance cobalt-based electrocatalysts, ultimately advancing their practical implementation in sustainable nitrate conversion and ammonia synthesis.

## Electrocatalytic NO_3_RR Mechanism on Cobalt-based Materials

The NO_3_RR is a complex process for electrocatalysts, involving various intermediates and reaction paths. Nitrate ion in the electrolyte can be converted into multiple products, with NH_3_ and N_2_ being the most stable. Detecting and identifying the product composition is crucial for improving selectivity. The reduction of NO_3_^−^ to NH_3_ involves an eight-electron and 9-proton transfer process (NO_3_^−^  + 6H_2_O + 8e^−^  → NH_3_ + 9OH^−^), while to N_2_ involves a five-electron transfer. The NO_3_RR pathway is governed by a sophisticated interplay of multiple physicochemical parameters, including but not limited to solution-phase NO_3_^−^ concentration, applied electrochemical potential, local pH microenvironment at catalytic interfaces, density of active sites, and the fundamental cobalt electronic configuration. Synergistic optimization of these interdependent variables through meticulous engineering is prerequisite for achieving enhanced Faradaic efficiency (FE), product selectivity, and reaction yield in the nitrate conversion process.

### Reaction Pathways of Electrochemical Nitrate Conversion

Under the condition of low NO_3_^−^ concentrations (< 1 M), a direct reduction reaction mechanism dominates for most NO_3_RR studies, which undergoes multiple deoxygenation and hydrogenation processes. In this reaction mechanism, two pathways proceed: the electron transfer pathway and the atomic hydrogen.^13^ NO_3_^−^ in the solution is first adsorbed on the surface of the catalyst to form *NO_3_, which is commonly considered to be the rate-determining step. Then *NO_3_ is converted to *NO_2_ and further to *NO through one of reduction pathways mentioned above, which can be clarified by in situ Raman spectra and online differential electrochemical mass spectrometry (DEMS) [26].

**Fig. 1:**
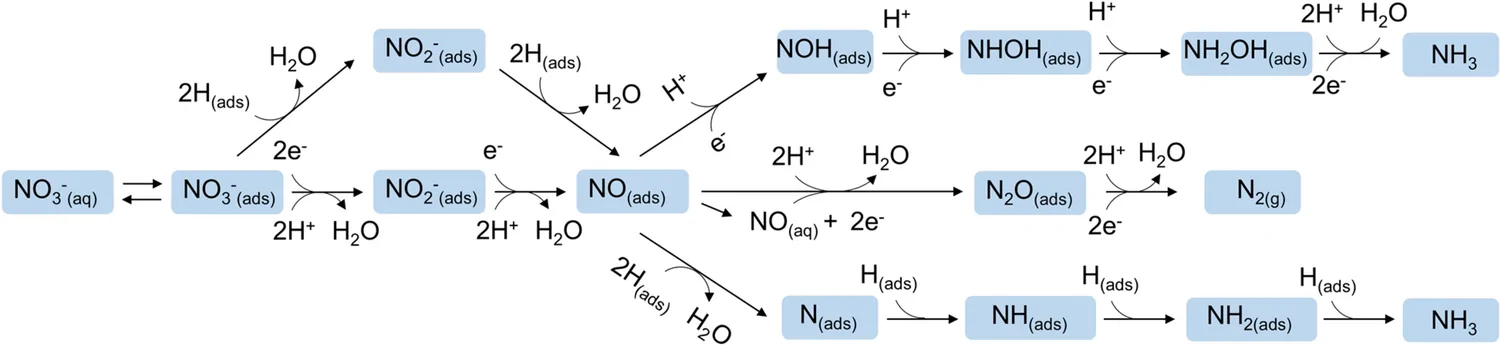
Direct mechanism for electrochemical NO_3_RR mediated by electron transfer and atomic hydrogen

As shown in Fig. 1, the rates of the subsequent steps that diverges at *NO will control the selectivity of the final product such as N_2_O, N_2_, NH_2_OH, and NH_3_ [27, 28]. For example, the dimerization of *NO could generate N_2_O or further to N_2_. Through the atomic hydrogen path, the *NO undergoes a series of hydrogenation procedures to obtain *N, *NH, *NH_2_, *NH_3_, and finally NH_3_, namely a subsequent Langmuir–Hinshelwood-like hydrogenation of *N by *H to ammonium. While for the electron transfer way, a reduction process proceeds through the intermediates of *NOH, *NOH_2_, *NOH_3_, *NOH_2_, and then NH_3_ can be obtained, which is also called as Eley–Rideal-like proton-coupled electron transfer progress-(PCET), and this reduction process has been detected by the in situ electrochemical FTIR characterizations [28–30]. The reaction processes can be described by the following equations:

1 $${\mathrm{NO}}_{{3}}^{ - } \to {\text{ NO}}_{{3}}^{ - } \left( {{\mathrm{ads}}} \right)$$

2 $${\mathrm{NO}}_{{3}}^{ - } \left( {{\mathrm{ads}}} \right) \, + {\text{ 2H}}^{ + } \, + {\text{ 2e}}^{ - } \to {\mathrm{NO}}_{{2}}^{ - } \left( {{\mathrm{ads}}} \right) \, + {\text{ H}}_{{2}} {\mathrm{O}}$$

3 $${\mathrm{NO}}_{{3}}^{ - } \left( {{\mathrm{ads}}} \right) \, + {\text{ 2H}}\left( {{\mathrm{ads}}} \right) \, \to {\mathrm{NO}}_{{2}}^{ - } \left( {{\mathrm{ads}}} \right) \, + {\text{ H}}_{{2}} {\mathrm{O}}$$

4 $${\mathrm{NO}}_{{2}}^{ - } \left( {{\mathrm{ads}}} \right) \, + {\text{ 2H}}^{ + } + {\text{ e}}^{ - } \to {\text{ NO}}\left( {{\mathrm{ads}}} \right) \, + {\text{ H}}_{{2}} {\mathrm{O}}$$

5 $${\mathrm{NO}}_{{2}}^{ - } \left( {{\mathrm{ads}}} \right) \, + {\text{ H}}\left( {{\mathrm{ads}}} \right) \, \to {\text{ NO}}\left( {{\mathrm{ads}}} \right) \, + {\text{ OH}}^{ - }$$

6 $${\mathrm{NO}}\left( {{\mathrm{ads}}} \right) \, + {\text{ NO}}\left( {{\mathrm{aq}}} \right) \, + {\text{ 2H}}^{ + } + {\text{ 2e}}^{ - } \to {\text{ N}}_{{2}} {\mathrm{O}}\left( {{\mathrm{ads}}} \right) \, + {\text{ H}}_{{2}} {\mathrm{O}}$$

7 $${\mathrm{N}}_{{2}} {\mathrm{O}}\left( {{\mathrm{ads}}} \right) \, + {\text{ 2H}}^{ + } + {\text{ 2e}}^{ - } \to {\text{ N}}_{{2}} \left( {\mathrm{g}} \right) \, + {\text{ H}}_{{2}} {\mathrm{O}}$$

8 $${\mathrm{NO}}\left( {{\mathrm{ads}}} \right) \, + {\text{ H}}^{ + } + {\text{ e}}^{ - } \to {\text{ NOH}}\left( {{\mathrm{ads}}} \right)$$

9 $${\mathrm{NOH}}\left( {{\mathrm{ads}}} \right) \, + {\text{ H}}^{ + } + {\text{ e}}^{ - } \to {\text{ NHOH}}\left( {{\mathrm{ads}}} \right)$$

10 $${\mathrm{NHOH}}\left( {{\mathrm{ads}}} \right) \, + {\text{ H}}^{ + } + {\text{ e}}^{ - } \to {\text{ NH}}_{{2}} {\mathrm{OH}}\left( {{\mathrm{ads}}} \right)$$

11 $${\mathrm{NH}}_{{2}} {\mathrm{OH}}\left( {{\mathrm{ads}}} \right) \, + {\text{ H}}^{ + } + {\text{ e}}^{ - } \to {\text{ NH}}_{{3}} + {\text{ H}}_{{2}} {\mathrm{O}}$$

12 $${\mathrm{NO}}\left( {{\mathrm{ads}}} \right) \, + {\text{ 2H}}\left( {{\mathrm{ads}}} \right) \, \to {\text{ N}}\left( {{\mathrm{ads}}} \right) \, + {\text{ H}}_{{2}} {\mathrm{O}}$$

13 $${\mathrm{N}}\left( {{\mathrm{ads}}} \right) \, + {\text{ H}}\left( {{\mathrm{ads}}} \right) \, \to {\text{ NH}}\left( {{\mathrm{ads}}} \right)$$

14 $${\mathrm{NH}}\left( {{\mathrm{ads}}} \right) \, + {\text{ H}}\left( {{\mathrm{ads}}} \right) \, \to {\text{ NH}}_{{2}} \left( {{\mathrm{ads}}} \right)$$

15 $${\mathrm{NH}}_{{2}} \left( {{\mathrm{ads}}} \right) \, + {\text{ H}}\left( {{\mathrm{ads}}} \right) \, \to {\text{ NH}}_{{3}} \left( {{\mathrm{ads}}} \right)$$

Although great development has been obtained toward the reaction mechanism, the exact reactive pathways during the NO_3_^−^ to NH_3_ conversion, as well as veritable active species across a wide potential range are still full of controversy. Moreover, the detailed reaction route can be greatly influenced by the catalytic sites of electrocatalysts, the pH of the electrolyte, the applied potential, and the anions in the electrolytes. The reaction routes vary hugely for different cobalt-based materials, for example, Co_3_O_4_ is mainly induced by the Co^2+^–Co^3+^–Co^2+^ redox cycle, and the synergistic effect of Co^3+^ and Co^2+^ in the redox cycle can be clarified as: Co^3+^ prefers the adsorption of NO_3_^−^ while Co^2+^ favors the production of H* for the hydrogenation, which has been evidenced by the density functional theory calculations, electron spin resonance analysis, and cyclic voltammetry [35, 36]. In another case, a Co-supported N-doping carbon catalyst (Co–N–C) represented an active hydrogen (H*) reduction mechanism during the reduction from NO_3_^−^ to NO_2_^−^, but a direct electron mechanism was predominated for the corresponding N-free catalyst (Co–C). While the subsequent reduction of NO_2_^−^ to NH_4_^+^ was conducted by the direct electron mechanism for both Co–N–C and Co–C [31]. A combination of kinetic studies, in situ spectroscopy, and computational work may be a powerful tool to understand the reaction mechanism and further explore efficient catalysts with satisfactory nitrate conversion.

### Regulation of Mechanism and Performance by Electronic Structure and Adsorption Behavior

The electrocatalytic NO_3_RR presents considerable complexity due to its multi-step reaction pathways and the competing hydrogen evolution reaction (HER). Despite these challenges, cobalt-based catalysts have emerged as promising candidates, demonstrating exceptional catalytic activity, remarkable FE, and superior ammonia selectivity, which are important parameters for evaluating the performance of the catalysts for NO_3_RR [32, 33]. These outstanding performance metrics are fundamentally governed by both intrinsic and extrinsic factors. The intrinsic catalytic properties of cobalt, including its unique electronic structure, optimal hydrogen chemisorption energy, and favorable nitric oxide adsorption characteristics, play a pivotal role in determining its catalytic behavior. Additionally, extrinsic operational parameters, particularly the applied electrochemical potential and electrolyte pH, significantly influence the reaction kinetics and product distribution. The interplay between these intrinsic material properties and external reaction conditions ultimately dictates the overall catalytic performance, making cobalt-based materials particularly suitable for selective nitrate-to-ammonia conversion [32, 33].

#### Electronic Structure with Work Function Descriptor

The electronic structure of active Co site has a great impact on the process of NO_3_RR, including the work functions, d-band center (E_d_) proximity to Fermi level, which are highly related with the HER activity, H chemisorption energies and NH_3_ selectivity strong exhibit significantly diminished NO_3_RR, ultimately affecting the performance of electrocatalytic activity, selectivity and FE. Carvalho et al. systematically investigated the role of electronic structure on the reduction process of nitrate to ammonia for a series of polycrystalline 3*d* (Ti, Fe, Co, Ni, Ni_0.68_Cu_0.32_, and Cu) and 4*d*^10^ (Ag) transition-metal foils (TMs), in terms of activity, selectivity and FE [39]. During the NO_3_RR progress, the HER reaction is kinetically easier to occur, resulting an insufficient FE and selectivity for many transition metals [39]. For the transition-metal binding H weakly (e.g., Ag, Cu), HER activity is limited by proton adsorption, while for metals binding H strongly (e.g., Fe, Ti), it is H−H bond-formation kinetics. HER activity can be also described by work function, where higher work function TMs (e.g., Ni) demonstrate greater HER activity than lower work function TMs [25, 39]. This can be explained that potential of zero charge becomes more positive as the metal work function increases, leading to enhanced electrostatic attraction of cations (i.e., protons for HER) at relevant NO_3_RR potentials [34, 42–40].

**Fig. 2:**
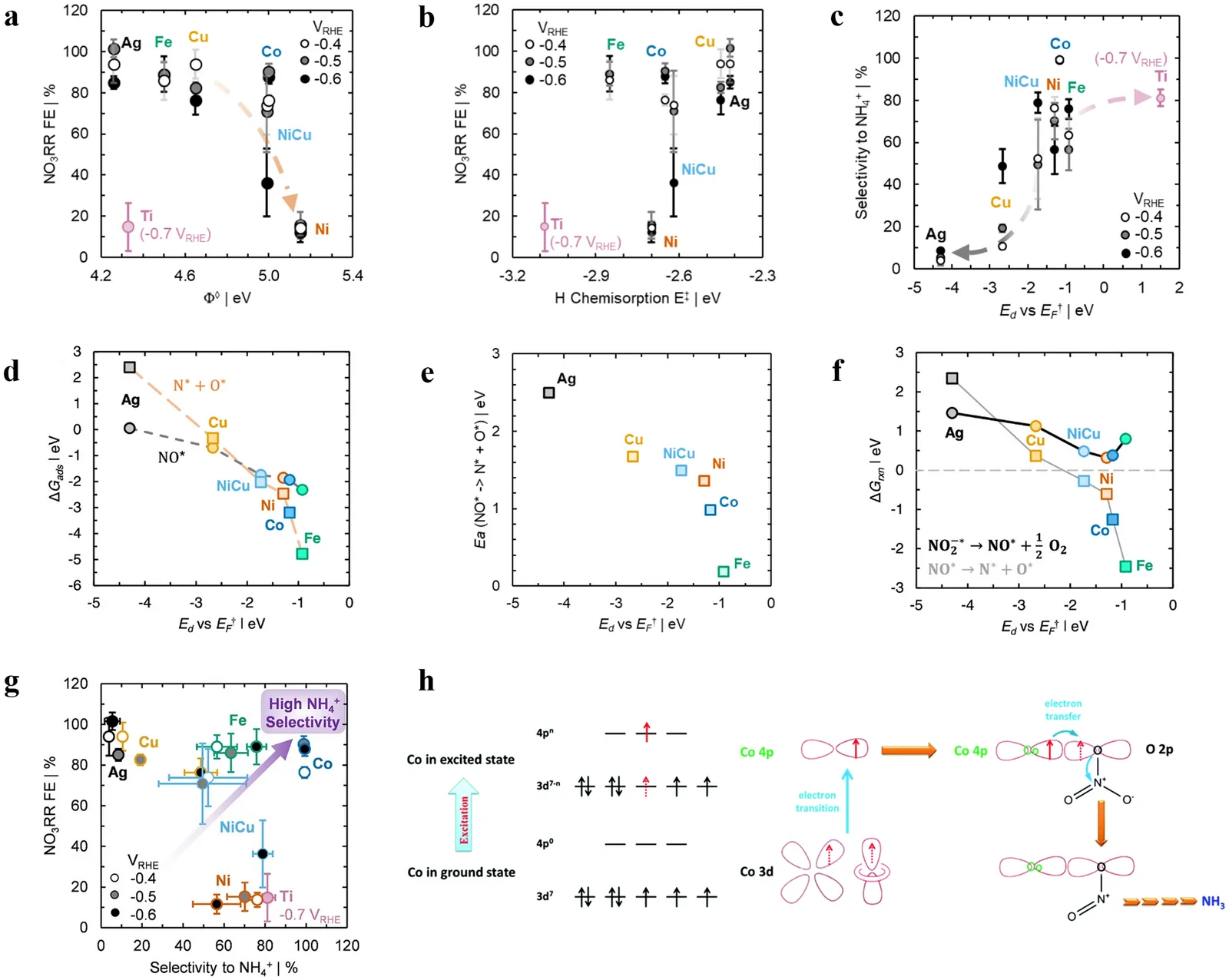
**a** NO_3_RR FE against work function [39]. Copyright 2022, American Chemical Society. **b** NO_3_RR FE against H chemisorption energy [39]. Copyright 2022, American Chemical Society. **c** Selectivity toward ammonium against E_d_ vs E_F_ [39]. Copyright 2022, American Chemical Society. **d** Free energy of associative (circles with short black dashes) and dissociative (squares with long orange dashes) nitric oxide adsorption against E_d_ vs E_F_ for denoted TM surfaces [39]. Copyright 2022, American Chemical Society. **e** Activation barrier (Ea) for dissociation of adsorbed nitric oxide against E_d_ vs E_F_ [39]. Copyright 2022, American Chemical Society. **f** Reaction free energies (ΔG_rxn_) of nitrite reduction to nitric oxide (NO*, circles with thick black lines) and nitric oxide dissociation (squares with thin gray lines) against E_d_ vs E_F_ for the denoted TMs; the dashed gray line denotes zero reaction free energy for clarity [39]. Copyright 2022, American Chemical Society. **g** NO_3_RR FE against selectivity to ammonium for a series of TM foils, measured in stirred 0.1 M Na_x_H_3−x_PO_4_ with 0.1 M NaNO_3_ by 85% iR-corrected chronoamperometry to 0.2 e^−^/NO_3_^−^ at − 0.4, − 0.5, and − 0.6 V_RHE_ (denoted) [39]. Copyright 2022, American Chemical Society. **h** Mechanism of the NO_3_RR on CoP NAs/CFC. Reproduced with permission [43]. Copyright 2022, Royal Society of Chemistry

The FE of the NO_3_RR is fundamentally governed by the competitive electron transfer between NO_3_RR and the HER, which is strongly correlated with two key descriptors: the hydrogen adsorption affinity (H* binding energy) and the work function of the catalyst surface. As illustrated in Fig. 2a, b, TMs with optimal combinations of low work functions and moderate H chemisorption energies, such as Ag, Cu, Co, and Fe, consistently demonstrate superior NO_3_RR FE exceeding 70%. In contrast, TMs with either excessively high work functions (e.g., Ni) or strong H chemisorption energies exhibit significantly diminished NO_3_RR performance.

This behavior can be attributed to distinct mechanistic origins: (1) Metals with high work functions facilitate HER through enhanced proton attraction, resulting in negatively charged surfaces that electrostatically favor proton adsorption and subsequent H−H coupling kinetics. (2) Conversely, metals with low work functions but excessively strong H chemisorption tends to form H*-saturated surfaces that hinder both nitrate adsorption and PCET kinetics, thereby impeding the hydrogenation steps crucial for NO_3_RR.

#### Regulation of Adsorption Behavior

Different adsorption behaviors and modes have a significant impact on the catalytic reaction pathways and mechanisms, also affect the expression of performance. The type of catalyst, voltage and local microenvironment (such as pH) significantly affect the way of adsorption. Cobalt's optimal H chemisorption energy and balanced HER activity enable efficient NO_3_RR (93.7% FE). Ammonium selectivity correlates with *d*-band center (E_d_) proximity to Fermi level (E_F_): E_d_ > E_F_ enhances selectivity, whereas low-E_d_ metals (Cu/Ag) exhibit minimal NH_4_^+^ yield. While Ni, Co, Fe, and Ti showed nominally high selectivity toward ammonium as their E_d_ approaching or above E_F_, as shown in (Fig. 2c). As explained by d-band model, the antibonding molecular orbital formed between the TM surface and adsorbed nitric oxide (NO*) becomes increasingly unoccupied as E_d_ approaches and overcomes E_F_, manifesting a stronger binding of NO* and a preference toward dissociative adsorption (NO* → N* + O*); then the N* will selectively form ammonium in the end [39].

On the other hand, the high ammonium selectivity of Co can be explained by the intermediate adsorbate energy. Nitrite (NO_2_^−^) plays a key role during the NO_3_RR process, and is highly related with the ammonium selectivity. Nitrate (NO_3_^−^*) is first reduced to NO_2_^−^*, and then NO* (NO_2_* → NO* + O). It is acknowledged that the surfaces of catalysts which favor dissociative nitric oxide adsorption (NO* → N* + O*) and subsequent N* adatom hydrogenation by H* (N* + H* → NH*) show a higher selectivity toward ammonium. Theoretical calculations identified that free energy of nitric oxide adsorption (ΔGNO*) became more negative as TM E_d_ approaches E_F_, as shown in Fig. 2d. Furthermore, dissociation activation barriers (NO* → N* + O*) decrease with E_d_ vs E_F_ increase (Fig. 2e). These suggested preferential associative adsorption on weak-binding TMs (ΔGNO* < ΔGN* + O*; e.g., Ag and Cu), and dissociative adsorption on strong binding TMs (ΔGNO* > ΔGN* + O*; e.g., Ni, Co, Fe), as shown in Fig. 2f (squares with thin gray lines). These results preference of nitrite reduction to nitric oxide (further to ammonium) over desorption, and a higher selectivity toward ammonium can be obtained for the later (Ni, Co, Fe). Interestingly, Co represents an optimal ammonium selectivity (> 95%), much higher than Fe and Ni (Fig. 2c). This can be explained through the calculated reaction free energies of nitrite reduction to nitric oxide and its further dissociation. As inferred above, nitrite reduction is more favorable on Ni than Co, but a poorer ammonium selectivity than Co. For this, Ni has a much lower driving force for NO* dissociation than Co, which can be indicated by the reaction free energies (ΔG_rxn_) of NO* dissociation for Co and Ni, as shown in Fig. 2f (squares with thin gray lines). In contrast, while NO* dissociation is more favorable on Fe compared to Co, nitrite reduction on Fe is less favorable (Fig. 2f, circles with thick black lines), resulting in greater nitrite selectivity on Co. In summary, Co could maximize ammonium selectivity for two factors: (1) strong nitrite binding benefitting subsequent reduction to ammonium, (2) promotion of nitric oxide dissociation, and subsequent selective reduction of the nitrogen adatom (N*) to ammonium [32].

As illustrated above, Co shows an optimal performance among the transition metals during NO_3_RR process either for FE and ammonium selectivity due to the appropriate work function, H chemisorption energy, and E_d_ vs E_F_ of Co, as shown in Fig. 2g. These results provide design considerations that Co-based catalysts must have sufficient H* affinity to reduce nitrate without activating the HER pathway or hindering nitrate hydrogenation kinetics, as well as appropriate E_d_ sufficiently close to E_F_ to activate the dissociation of nitric oxide [32, 39]. These identify competing design considerations, where TMs must have sufficient H* affinity to reduce nitrate without activating the HER pathway or hindering nitrate hydrogenation kinetics. Further, TMs must have E_d_ sufficiently close to E_F_ to activate the dissociation of nitric oxide, but not too close where nitrite reduction becomes selectivity-limiting. These results identify competing design considerations linking electronic structure to mechanistic selectivity-limiting steps offering strategies to improve existing catalysts and design new alloy compositions or and design new Co-based materials for NO_3_RR in the future.

In addition to catalysts, external added factors (such as voltage and catalytic microenvironment) also have a significant impact on the regulation of catalytic mechanisms and performance. The potential applied in the electrocatalytic system is determined by the efficiency of energy conversion, thus reducing the applied potential and the resulting overpotential is indispensable for a high and commercially viable energy efficiency (EE) [26]. Overpotential needed for the reaction depends on the maximum Gibbs free energy change (ΔG_max_) been different reaction intermediates, which is also signified as the potential determining step (PDS) of NO_3_RR progress. Many previous studies present that the PDS is *NO_3_ dissociation to *NO_2_, meaning that the reduction rate of NO_3_^−^ to NO_2_^−^ is often slow and requires high activation energy. In fact, this slow process can be attributed to the high-energy of lowest unoccupied molecular π* orbital (LUMO π*) for NO_3_^−^. The d orbitals of transition metals (except Hg) are hard to match with the LUMO of NO_3_^−^ ion, therefore, electrons are difficulty to be injected into the π* orbital of NO_3_^−^ ion, resulting a high overpotential for triggering NO_3_RR [26, 35, 39]. Ye et al. systematic studied the reactive mechanism of NO_3_RR on metallic Co and CoP; they revealed that Co 4p orbitals may directly participate in the nitrate adsorption and electron transfer step of the NO_3_RR. The results of operando X-ray absorption fine structure (XAFS) implied that electrons in the Co 3d orbitals were excited to 4p orbitals when NO_3_RR was triggered. Further, it could be inferred that the excited electrons were transferred to the O 2*p* orbitals of nitrate via the Co–O–N covalent bond and injected into the π* orbitals of NO_3_^−^, then the adsorbed NO_3_^−^ was destabilized and eventually reduced, as shown in Fig. 2e. According to this, upshifting 3d orbitals to reduce the energy gap between 3*d* orbitals and 4*p* orbitals of metal sites might be an alternative to reduce the overpotential of NO_3_RR.

### Durability and Degradation Mechanisms

During the NO_3_RR progress, the NO_x_ intermediate products, as well as the hydrogen solid solution, surface coordination hydrogen compounds (M-H_ads_) and hydride formation may block the active sites and lead to surface poisoning, shortening the lifespan of the catalyst [39–45]. For example, the generated H_ads_ in NO_3_RR can serve as hydrogen sources, but may also lead to surface poisoning due to competitive adsorption on active site, namely the H-poisoning [46]. Furthermore, the local strong alkaline microenvironment can corrode metal sites during the NO_3_RR process, leading to problems such as oxidation and dissolution of metal sites, therefore, the stability of the catalyst is crucial. Self-reconstruction of the catalysts is a common phenomenon during the electrocatalytic process. However, the reconstruction may create new active sites, promoting the NO_3_RR process, or destroy the predesigned active site structures, leading to reduced performance and stability of the catalyst [47–50]. Both the physical and chemical driving forces take part in the reconstruction, among which physical driving forces lead to cracks, detachment of the catalytic layer, and change of the particle size, probably leading to the collapse of electrocatalyst and a rapid decline of the NO_3_RR performance. Gas bubble generation/breaking, electrolyte convection, and random atom immigration may cause these physical driving forces [51]. While the chemical driving force causes the change of valence state, leaching/redeposition, ion-exchanging, and new phase [43]. Oxidation/reduction, chemical adsorption/desorption, intercalation/de-intercalation of ions/molecules, combination, hydrolysis, dissociation, and double decomposition of pre-catalyst are the familiar chemical driving forces. Differently, these in situ reconstruction driven by chemical driving force may be helpful when it may provide open structures or create new active sites for electrocatalysis. Otherwise, it may hamper the electrocatalytic reactions [43]. For one case, the CoO_x_ skeletons in the spinel may collapse in the NO_3_RR and HER condition, while the crystalline/amorphous domains (nanocrystalline glass-like structure) to stabilize the CoO_6_ and CoO_4_ of Co oxide, and obtained a satisfactory durability [26].

Self-reconstruction of the catalysts is a common phenomenon during the electrocatalytic process. For a typical case, CoP is widely used as catalysts for NO_3_RR due to the low cost and high catalytic activity across a wide potential range, however, many reports proved the formation of Co(OH)_2_ on the surface of CoP catalysts after NO_3_RR [43]. Due to the oxyphilic nature of phosphorus (P) element, some researchers attributed this phenomenon to the inevitable oxidation of superficial Co and then ignored the role of Co(OH)_2_ [43, 50]. In contrast, other researchers believed that the CoP/Co(OH)_2_ interface could promote the dissociation of H_2_O to release active hydrogen (*H), thereby assisting NO_3_RR [20, 29]. In another case, Qiao et al. found that the incorporation nickel into Co_3_O_4_ can improve the NO_3_RR performance. The nickel incorporated Co_3_O_4_ (Co_2_NiO_4_) was reconstructed to cobalt nickel hydroxides (Co_y_Ni_1−y_(OH)_2_) under catalytic conditions, which could promote the step of *NO to *NOH and surpass HER process [47].

For Co-based alloys, it is usually considered that superficial Co species work as active sites for nitrate continuous hydrogenation to ammonia [38, 39]. Very recently, Yu’s group reported a non-electrochemical electron transfer process between metallic Co^0^ and nitrate and a dynamic transformation between Co^δ+^ and Co^0^ on a RuCo catalyst, which will be detailed discussed in the next section [72]. In another work, they adopted CoP, Co, and Co_3_O_4_ as model materials to further investigate the reaction mechanism, in terms of the self-reconstruction and the effect of applied potential [19]. They found that the P element in the superficial CoP will be quickly dissolved after exposure to KOH solution, and a core@shell structured CoP@Co(OH)_2_ was obtained, which formed CoP@Co by a pre-reduction process before NO_3_^−^ reduction. For this core@shell CoP@Co and metallic Co catalysts, a three-step relay mechanism was inferred over superficial dynamical Co^δ+^ active species under low applied potential:

16 $${\mathrm{NO}}_{{3}}^{ - } + {\text{ Co }} + {\text{ H}}_{{2}} {\text{O }} \to {\text{ NO}}_{{2}}^{ - } + {\text{ Co}}\left( {{\mathrm{OH}}} \right)_{{2}}$$

17 $${\mathrm{Co}}\left( {{\mathrm{OH}}} \right)_{{2}} + {\text{ e}}^{ - } \to {\text{ Co }} + {\text{ 2OH}}^{ - }$$

18 $${\mathrm{NO}}_{{2}}^{ - } + {\text{ 5H}}_{{2}} {\text{O }} + {\text{ 6e}}^{ - } \to {\text{ NH}}_{{3}} + {\text{ 7OH}}^{ - }$$

While a continuous hydrogenation mechanism dominated over superficial Co species under high applied potential. In comparison, Co_3_O_4_ species were stable and steadily reduced nitrate by a hydrogenation progress across a wide potential range. It was also found that CoP@Co and Co exhibited much higher NO_3_RR activity than Co_3_O_4_ especially under a low applied potential, and the conversion performance of CoP@Co was higher than Co, although both of which experienced the same reaction mechanism. This could be explained that CoP core donated abundant electrons to superficial active species, benefiting the generation of active hydrogen for the reduction of nitrogen-containing intermediates.

Although pH value also influences the NO_3_RR process, there are few works focusing on this effect for Co-based materials. Even so, the pH value influencing mechanism on other materials may give us helpful understanding and guidance to design more efficient Co-based catalysts. For example, Guo’s group conducted a systematic study of the pH influence on NO_3_RR on copper through density functional theory (DFT) calculations [12]. They found the RDS was hydrogenation of *NO (*NO + H^+^  + e^–^ → *NOH) under pH of 14 or 7, while moved to the desorption of *NH_3_ at pH of 0. The HER dominated at pH 0, but in neutral media, HER was greatly suppressed, and a predominance of NO_3_RR over HER could be observed. Other reports such as Ti cathode were also in agreement with this trend [41].

In terms of specific materials, amorphous materials with long-range disordered structures have exhibited superior electrocatalytic performance and durability compared to their crystalline counterparts [50–52]. For single/dual atom catalysts, it needs resistance to corrosion and degradation caused by electrolyte species, pH changes, and electrode potential cycling. As for tandem systems, the stability and durability of these composites under the harsh conditions of electrolysis are a concern for practical application due to their complex composite .

**Table 1:** Some typical parameters of wastewater discharged from the factory workshop

Parameters	Unit	Value
pH	–	7.4
Suspended solids (SS)	mg L^−1^	2079 ± 251
Chemical oxygen demand (COD)	mg L^−1^	< 12
Conductivity	mS cm^−1^	37.8 ± 1.3
Ba^2+^	mg L^−1^	11,773 ± 285
Cl^−^	mg L^−1^	45.2 ± 3.9
SO_4_^2−^	mg L^−1^	3824 ± 29
NO_3_^−^ -N	mg L^−1^	2174 ± 12
NH_4_^+^ -N	mg L^−1^	3.6 ± 0.8

As Co-based materials show great potential for large-scale applications, it is necessary to evaluate the catalyst performance under the actual wastewater, in which the coexistence of organic and ionic impurities may have a certain adverse effect on the kinetics of NO_3_RR. Duan et al. evaluated the NO_3_RR performance of Co metal and other 15 types of commercially available metal foils in real wastewater sample, where multiple ionic impurities and suspended solids exist, as shown in Table 1 [53]. They found that Co exhibited higher performance than other metal foils, and a long-term stability of the Pb, Sn, In, and Co cathodes was assessed to evaluate the reliability and repeatability of their NO_3_RR activity. Interestingly, upon 6 cycles of electrolysis, metal ions were leached from the cathodes, with values of 0.05, 0.03, 0.01, and 0.008 mg L^−1^ for Co, Sn, In, and Pb, respectively. This may be attributed to the high alkalinity at the cathode surface, which can attack the metals. It is worth noting that the complete inhibition of metal dissolution is a critical requirement for potential practical applications, and addressing this issue will be an important focus for future research [53]. High-performance catalysts for which insufficient discussion on the complex factors in actual wastewater treatment, such as coexisting ion interference, long-term operational stability, and catalyst recovery costs.

**Table 2:** Summary of the electrochemical performance of some Co-based electrodes for NO_3_RR

Materials	Electrolyte	NH_3_ yield (vs RHE)	Faradaic efficiency (vs RHE)	Refs.
Co nanoarray	1 M KOH with 0.1 M NO_3_^−^	10.4 mmol h^−1^ cm^−2^ at − 0.24 V	100% at − 0.14 V > 96% at -0.24 ~ 0.11 V	[28]
Ru_15_Co_85_	0.1 M KOH with 0.1 M KNO_3_	3.2 ± 0.17 mmol h^−1^ mg_cat_^−1^ at + 0 V	97 ± 5% at + 0 V	[72]
Co_0.5_Cu_0.5_	1 M KOH with 0.05 M KNO_3_	> 0.235 mmol h^−1^ mg ^−1^ at − 0.15 V	> 95% at − 0.03 V	[71]
Cu_2.5_Co	0.5 M Na_2_SO_4_ with 0.1 M KNO_3_	0.164 mmol h^−1^ cm^−2^ at − 0.25 V	96.29% at − 0.05 V	[69]
Au nanocrystals-Co_3_O_4_	1 M KOH with 0.5 M KNO_3_	0.0016 mmol h^−1^ cm^−2^ at 0.437 V	83.1% at 0.437 V	[84]
Co_3_O_4_-NS/Au-NWs	0.5 M K_2_SO_4_ with 0.05 M KNO_3_	0.156 mmol h^−1^ mg^−1^ at − 0.5 V	97.76% at − 0.5 V	[83]
Co_3_O_4_/Co	0.1 M Na_2_SO_4_ with 1 mg mL^−1^ of NO_3_^−^	0.26 mmol h^−1^ cm^−2^ − 0.8 V	88.7% at − 0.8 V	[85]
Co_3_O_4_/Cu	0.4 M Na_2_SO_4_ with 50 mg L^−1^ NO_3_^−^	0.04 mmol h^−1^ mg^−1^ − 0.4 V	94.6% at—0.4 V	[89]
CuO NWAs@Co_3_O_4_	1 M KOH with 1400 ppm NO_3_^−^	1.915 mmol h^−1^ cm^−2^ − 0.23 V	99.17% at − 0.23 V	[87]
Cu_2_O + Co_3_O_4_	0.1 M NaOH with 0.1 M NO_3_^−^	0.75 mmol h^−1^ cm^−2^ at − 0.3 V	85.4% at − 0.3 V	[113]
Cu/CuO_x_ Co/CoO	0.1 M NO_3_^−^ with 0.1 M KOH	1.17 mmol h^−1^ cm^−2^ − 0.175 V	93.3 ± 2.1% at − 0.175	[114]
Fe-doped Co_3_O_4_ nanoarray	0.1 M KH_2_PO_4_ and 0.05 M K_2_HPO_4_ with 0.05 M KNO_3_	0.037 mmol h^−1^ mg^−1^ at − 0.7 V	95.5% at − 0.7 V	[91]
Mn-incorporated Co_3_O_4_	0.5 M K_2_SO_4_ + 0.1 M KNO_3_	2.06 mmol h^−1^ cm^−2^ at − 1.2 V	99.5% at − 1.2 V	[95]
CoO_x_ nanosheets	0.1 M KOH with 0.1 M KNO_3_	4.85 mmol h^−1^ mg^−1^ at 0.3 V	93.4 ± 3.8% at − 0.3 V	[92]
CoO NWA/TM	0.1 M NaOH with 0.1 M NO_3_^−^	0.4 mmol h^−1^ cm^−2^ at − 0.6 V	95.1% at − 0.6 V	[93]
CoP nanoshuttles	0.5 M K_2_SO_4_ + 0.05 M KNO_3_	1.134 mmol h^−1^ cm^−2^ at 0.5 V	94.24 ± 2.8% at − 0.5 V	[111]
CoP/TiO_2_@TP	0.1 M NaOH with 0.1 M NO_3_^−^	0.499 mmol h^−1^ cm^−2^ at − 0.5 V	95.0% at − 0.3 V	[115]
Co_2_P nanodendrites	0.5 M Na_2_SO_4_ with 0.1 M NaNO_3_	0.3 mmol h^−1^ cm^−2^ at − 0.6 V	∼90% − 0.4 V	[116]
CoB_x_	0.1 M KOH with 0.05 M KNO_3_	0.787 mmol h^−1^ cm^−2^ at − 1.3 V	94.00 ± 1.67% at − 0.9 V	[117]
3D ZnCo_2_O_4_	~	0.124 mmol h^−1^ mg^−1^ at − 0.6 V	95.4% at − 0.4 V	
ZnCo_2_O_4_ NSA/CC	0.1 M NaOH with 0.1 M NaNO_3_,	0.634 mmol h^−1^ cm^−2^ at − 0.8 V	98.33% at − 0.6 V	[96]
CuCo_2_O_4_/CFs	1 M KOH with 0.1 M NO_3_^−^	0.394 mmol h^−1^ mg^−1^ at − 0.3 V	81.9% at − 0.3 V	[97]
Co_2_AlO_4_/CC	0.1 M PBS with 0.1 M NO_3_^−^	0.465 mmol h^−1^ cm^−2^ at − 0.9 V	92.6% at − 0.7 V	[101]
Ni_3_Co_6_S_8_	1 M KOH 50 mg L^−1^ NO_3_^−^	0.14 mmol h^−1^ cm^−2^ at − 0.4 V	85.3% at − 0.4 V	[103]
CuCo-CoO	1 M KOH and 0.1 M KNO_3_	12.63 mmol h^−1^·cm^−2^ at − 0.5 V vs	96.68% at − 0.3 V	[118]
LaCoO_3_ perovskite	1 M Na_2_SO_4_ and 0.5 M KNO_3_	4.18 mmol h^−1^ mg^−1^ at − 1.0 V	91.5% at − 1.0 V	[108]
CoP_1_N_3_	0.02 M Na_2_SO_4_ with 100 g/L N-NO_3_	0.025 mmol h^−1^ cm^−2^ at − 0.69 V	92.0% at − 0.69 V	[119]
Co doping TiO_2_	0.1 M NaOH with 0.1 M NO_3_^−^	1.127 mmol h^−1^ cm^−2^ at − 0.9 V	98.2% at − 0.5 V	[120]
CoPc@ RGO	0.1 M K_2_SO_4_ with 200 ppm KNO_3_	0.0035 mmol h^−1^ mg ^−1^ at − 0.2 V	95.12% at − 0.2 V	[121]

## Co-based Materials with Different Coordination Environment

Cobalt, a rare magnetic element akin to iron and nickel, predominantly exists as Co(II) in industry and occurs naturally in arsenides, oxides, and sulfides [26, 45]. As noted, the electronic structure of Co sites critically governs NO_3_RR performance. This structure can be tuned via coordination environments (e.g., Co–O, Co–P, Co–Cu bonds), which are extensively discussed below.

### Monometallic Cobalt (Co–Co Coordination)

Metallic cobalt and its alloys are strategic resources widely used in equipment manufacturing, catalysis, and energy storage due to their low cost, mechanical strength, corrosion resistance, and stability [42–44, 54–56]. Co–Co coordination bond predominates in these metallic cobalt materials, which exhibits high electrocatalytic ability for nitrate electroreduction and low HER activity [46]. This chapter summarizes the application of monometallic cobalt in electrocatalytic NO_3_RR.

#### Pure Cobalt Nanomaterials

**Fig. 3:**
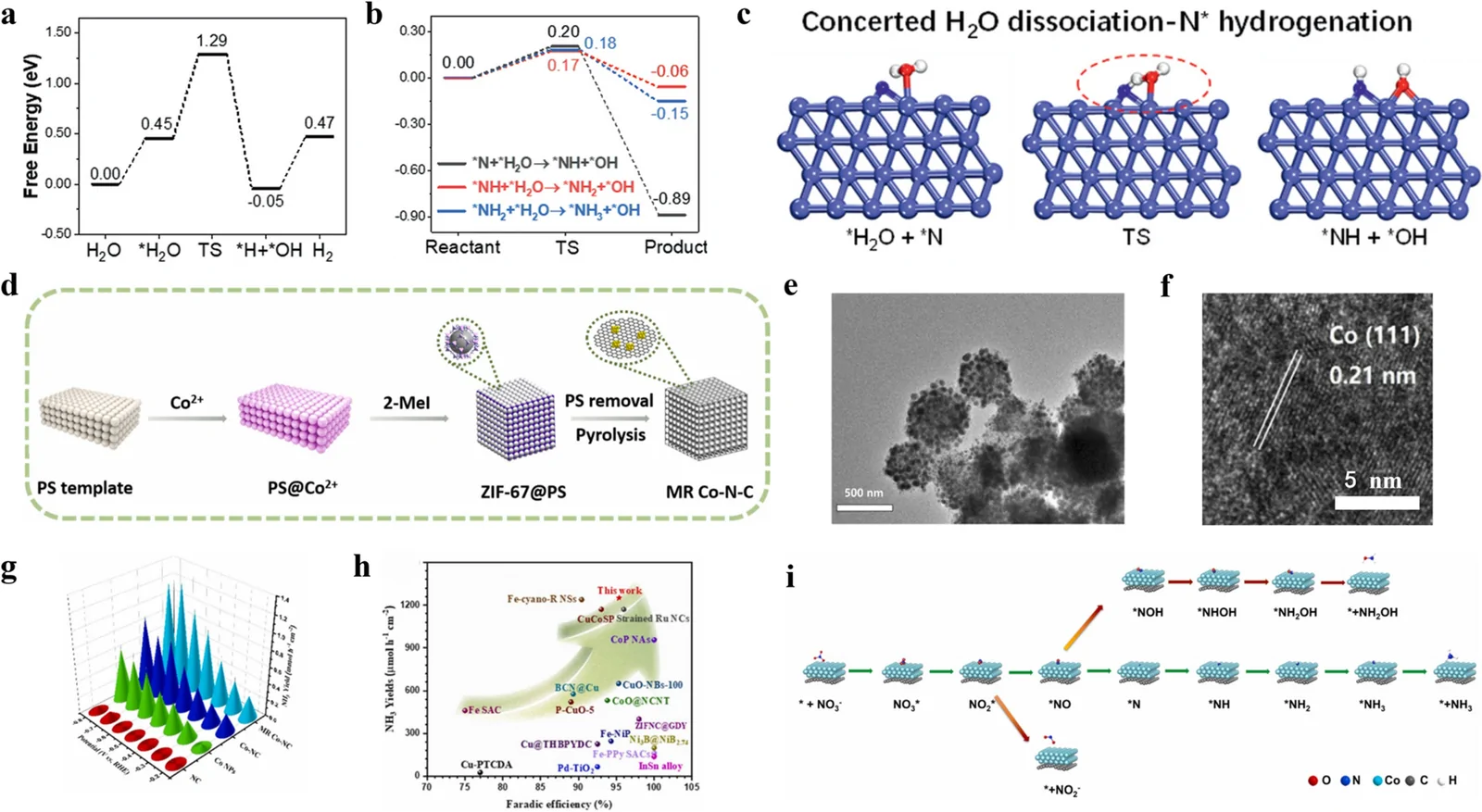
**a** Reaction free energies for intermediates on Co (111) toward HER [28]. Copyright 2021, Wiley–VCH. **b** Reaction free energies by considering the concerted H_2_O dissociation and hydrogenation of *N, *NH, and *NH_2_, TS represents transition state [28]. Copyright 2021, Wiley–VCH. **c** Proposed initial state, transition state, and final state for *N + *H_2_O → *NH + *OH on Co (111) surface. The light blue, blue, red, and white spheres represented Co, N, O, and H atoms, respectively [28]. Copyright 2021, Wiley–VCH. **d** Schematic illustration of the synthesis process of MR Co-NC [57]. Copyright 2023, Elsevier. **e** TEM and **f** HRTEM image of MR Co-NC [57]. Copyright 2023, Elsevier. **g** Yield for Co NPs, NC, Co-NC, and MR Co-NC under the potential range of − 0.2 ~  − 0.8 V vs RHE [57]. Copyright 2023, Elsevier. **h** FE values and NH_3_ yields of MR Co-NC compared with other recently reported catalysts [57]. Copyright 2023, Elsevier. **i** The proposed reaction pathways for the production of NH_3_, NH_2_OH and NO_2_^−^, where the minimum energy pathway is shown in green arrows [57]. Copyright 2023, Elsevier

Before using the metallic Co as catalysts for NO_3_RR, Ru- and Cu-based electrocatalysts were the most active and selective candidates. However, the achieved current density toward NO_3_RR remains lower than 200 mA cm^−2^ in most cases, limiting the practical application in NH_3_ production. To explore the catalyst library and solve the bottleneck problem of current density, Deng et al. reported a catalyst of HER-inert monometallic Co nanoarrays, which were obtained from the electrochemical reduction of Co(OH)_2_ nanoarrays, and studied the reaction mechanism in detail [28]. These Co nanoarrays consisted of abundant nanosheets and possessed large surface-active sites; therefore, they could exhibit a current density of − 2.2 A cm^−2^ and NH_3_ production rate of 10.4 mmol h^−1^ cm^−2^ at − 0.24 V vs. RHE under alkaline conditions. In addition, the catalyst showed a high FE toward NH_3_ (≥ 96%) over a wide application range (0.11 to − 0.24 V vs. RHE) and good long-term electrolysis stability at − 500 mA cm^−2^ for 10 h. DFT calculations revealed that metallic Co exhibited optimized intermediate adsorption energy compared with pristine Co(OH)_2_; however, the Volmer step (H_2_O → *H + *OH) exhibited an overall energy barrier of 1.29 eV, as shown in Fig. 3a, reflecting the sluggish water dissociation kinetic. And this could be well correlated with the poor HER activity on Co nanoarrays, which was contradictory to the exceptional NH_3_ producing capability at potentials more positive than HER onset (− 0.1 V vs. RHE). Thus, the authors speculated that the reduction intermediates *N, *NH, and *NH_2_ could functionalize as nucleophiles and were directly participating in the deprotonation (dissociation) of adsorbed H_2_O molecules, which had been supported by experimental and theoretical evidence (Fig. 3b, c).

By this way, the kinetically sluggish Volmer step could be omitted and a sufficient quantity of protons could be supplied from H_2_O molecules at potentials more positive than those required for HER. Furthermore, it is proposed that the water dissociation process was significantly facilitated by the interaction between surface adsorbed *H_2_O and nucleophilic *N, *NH, and *NH_2_ species. Finally, the authors considered that this concerted water dissociation-hydrogenation mechanism may open-up new opportunities in other catalytic systems. In another case, a three-dimensional porous Co foam supported on a Ti plate (Co foam/TP) was prepared by a simple electrodeposition method, and was used to drive the electrochemical NO_3_^−^-to-NH_3_ conversion [58]. Such Co foam/TP exhibited a maximum NH_3_ FE of 96.5% with a corresponding NH_3_ yield of up to 600.6 μmol h^−1^ cm^−2^ at − 0.4 V vs. RHE. Xu et al. designed a Co 3D nanoarray catalyst and used it as a cathode in a bipolar membrane flow reactor, which achieved a yield of 68.4 mg h^−1^ cm^−2^ and a FE of over 86.2% at 1000 mA cm^−2^ in 2000 ppm NO_3_^−^ alkaline electrolytes [48]. It is demonstrated that the Co 3D nanoarray cathode was full of abundant catalytic sites and the mass transfer was specifically fabricated due to the unique morphology, resulting a high efficiency. Besides, an excellent stability with a > 100 h operation at 1000 mA cm^−2^ was also obtained.

#### Synergistic Effect between Carrier and Pure Cobalt

Despite metallic Co exhibiting high activity for NO_3_RR, issues such as catalyst aggregation, oxidation, and subsequent reduction in electrical conductivity need addressing. A common approach to mitigate these problems is to composite cobalt with conductive carbon. This not only prevents particle aggregation but also boosts electrical conductivity, thereby enhancing catalytic performance. Based on this, Sun's group synthesized a catalyst comprising Co/N-doped carbon nanospheres derived from an adenine-based metal–organic framework (Co@NC) specifically for NO_3_RR [60]. Such Co@NC manifested a notable FE of 96.5% and a high NH_3_ yield of up to 758.0 μmol h^−1^ mg_cat_^−1^ in 0.1 M NaOH containing 0.1 M NO_3_^−^. Moreover, it also demonstrated strong electrochemical stability for recycling tests and bulk electrolysis. Similarly, another catalyst of Co nanoparticles decorated corncob-derived biomass carbon also achieved an amazing FE of 93.4% and a large NH_3_ yield of 0.60 mmol h^−1^ cm^−2^ in alkaline media [61]. Besides, favorable durability in long-term and cycle-electrolysis tests also demonstrated the advantage of composition with conductive carbon [50, 61].

Li’s group fabricated a catalyst of hierarchically mesoporous Co nanoparticles decorated with N-doped carbon, of which monodispersed polystyrene spheres (PS) were used as sacrificial templates (MR Co–NC), as shown in Fig. 3d–f [57]. Due to the large specific area of the pore-rich structures and the high intrinsic activity of metallic Co, the MR Co–NC showed a partial current density of 268 mA cm^−2^ with a FE of 95.35% ± 1.75% and a generation rate of 1.25 ± 0.023 mmol h^−1^ cm^−2^ for NH_3_ production, better than the other compared samples and many reported catalysts at that time, as shown in Fig. 3g, h. The porous carbon skeleton was found to play a dual role by simultaneously protecting the active sites from oxidation and facilitating long range charge and mass transfer. Theoretical calculations revealed a lower energy barrier of the rate-determining step (*NO_2_ + H_2_O + 2e^−^  → *NO + 2OH^−^) on the metallic Co of Co–NC over β-Co(OH)_2_ formed by the reconstruction of carbon-free Co nanoparticles (Fig. 3i).

Recently, Liu et al. used ZIF-67 as the precursor, prepared a Co-supported carbon material (Co–N–C) catalyst, and discussed in depth whether the electronic pathway or H* pathway dominated the electrocatalytic NO_3_RR process [62]. For this question, the authors also prepared a similar catalyst but not containing N as a contrast (Co–C). They found that chemical adsorption rather than electrostatic adsorption dominated among Co–N–C and free NO_3_^−^, which was significant for the subsequent electronic reduction process. Mechanism analysis indicated the Co–N_x_ bond formed by the introduction of N atoms could effectively utilize H* for NO_3_RR to NO_2_^−^, which suggested that an active hydrogen (H*) reduction mechanism was predominated for Co–N–C, but a direct electron mechanism was predominated for Co–C during the reduction from NO_3_^−^ to NO_2_^−^. While the subsequent reduction of NO_2_^−^ to NH_4_^+^ was conducted by the direct electron mechanism for both Co–N–C and Co–C. This work presented a strategy by an introducing Co–N bond that not only improved the performance of the NO_3_RR to ammonia but also provided a method to study the reaction mechanism and even control the reaction route. In addition, it also inspired the preparation of other Co-based functional materials for reducing the production of nitrite.

**Fig. 4:**
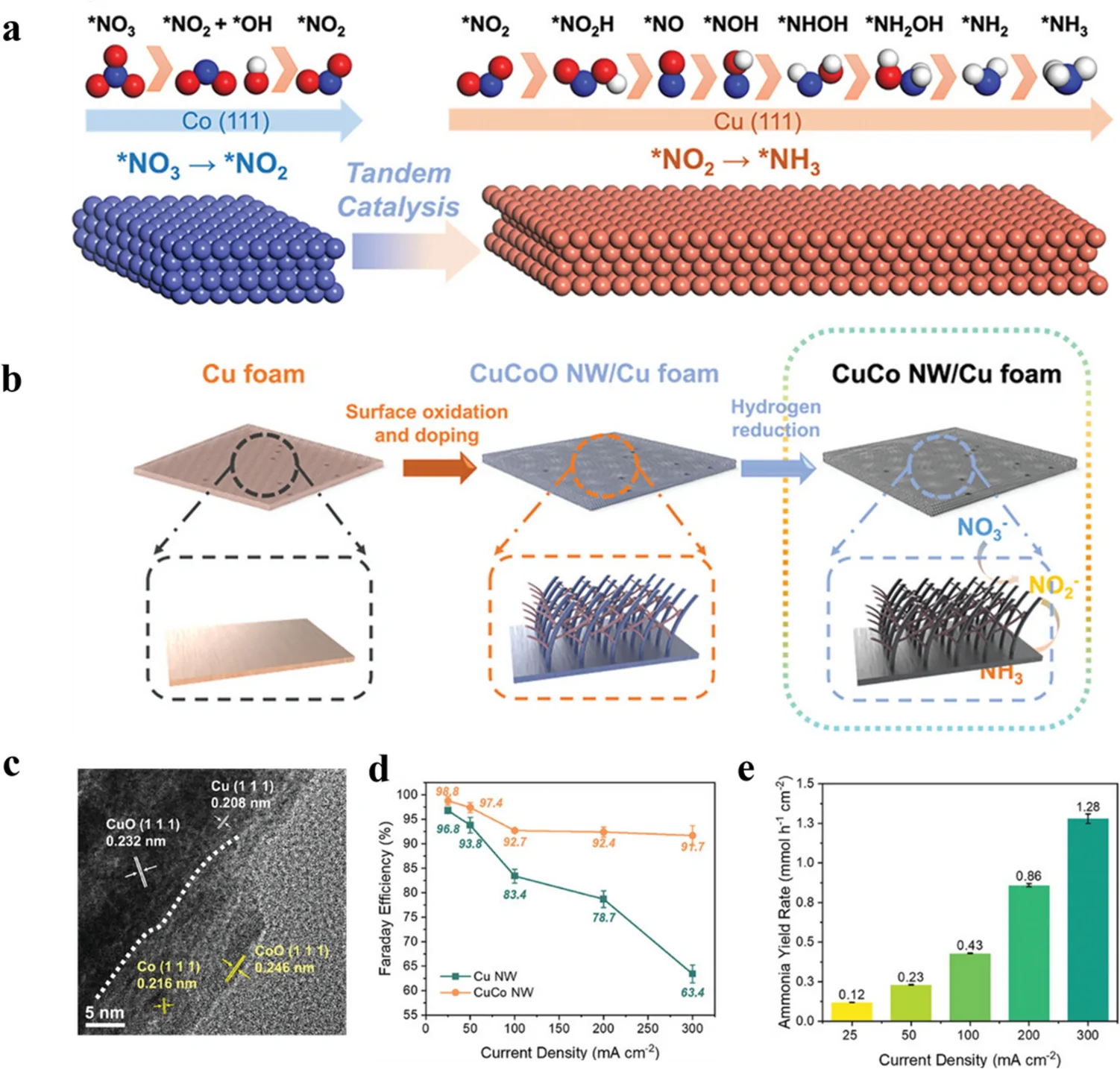
**a** Tandem catalysis mechanism of Co (111) and Cu (111) facets [63]. Copyright 2024, Wiley–VCH. **b** Synthesis process of CuCo NW/Cu foam [63]. Copyright 2024, Wiley–VCH. **c** HRTEM image of CuCo NW [63]. Copyright 2024, Wiley–VCH. **d** FE of ammonia production on CuCo NW and Cu NW under different current densities [63]. Copyright 2024, Wiley–VCH. **e** Ammonia yield rate of CuCo NW [63]. Copyright 2024, Wiley–VCH

An’s group developed a tandem catalysis strategy and prepared a composite of CuCo branched nanowires (CuCo NW) catalyst, which efficiently converted NO_3_^−^ to NH_3_ on Co (111) and Cu (111) crystal facets through a tandem catalysis mechanism, as shown in Fig. 4a, b; the HRTEM image of CuCo NW is shown in Fig. 4c [63]. The in situ grown CuCo NW on Cu foam demonstrated a remarkable FE of 90.3% at 1.0 A cm^−2^ and maintained stable operation for 200 h at 100 and 200 mA cm^−2^ in a flow reactor, as shown in Fig. 4d, e. Density functional theory calculations suggested that the initial absorption and subsequent deoxygenation of *NO_3_ on Co (111) leading to the formation of *NO_2_, followed by its transfer to Cu (111) and further conversion to *NH_3_, establish an optimal pathway by managing rate-determining steps on individual surfaces for NO_3_RR. Although tandem systems can utilize different materials with complementary properties, resulting an improved performance, there are still challenges for tandem electrocatalysts. The stability and durability of these composites under the harsh conditions of electrolysis are a concern for practical application. In situ reconstruction or degradation of the catalysts could occur, affecting their performance over time.

**Fig. 5:**
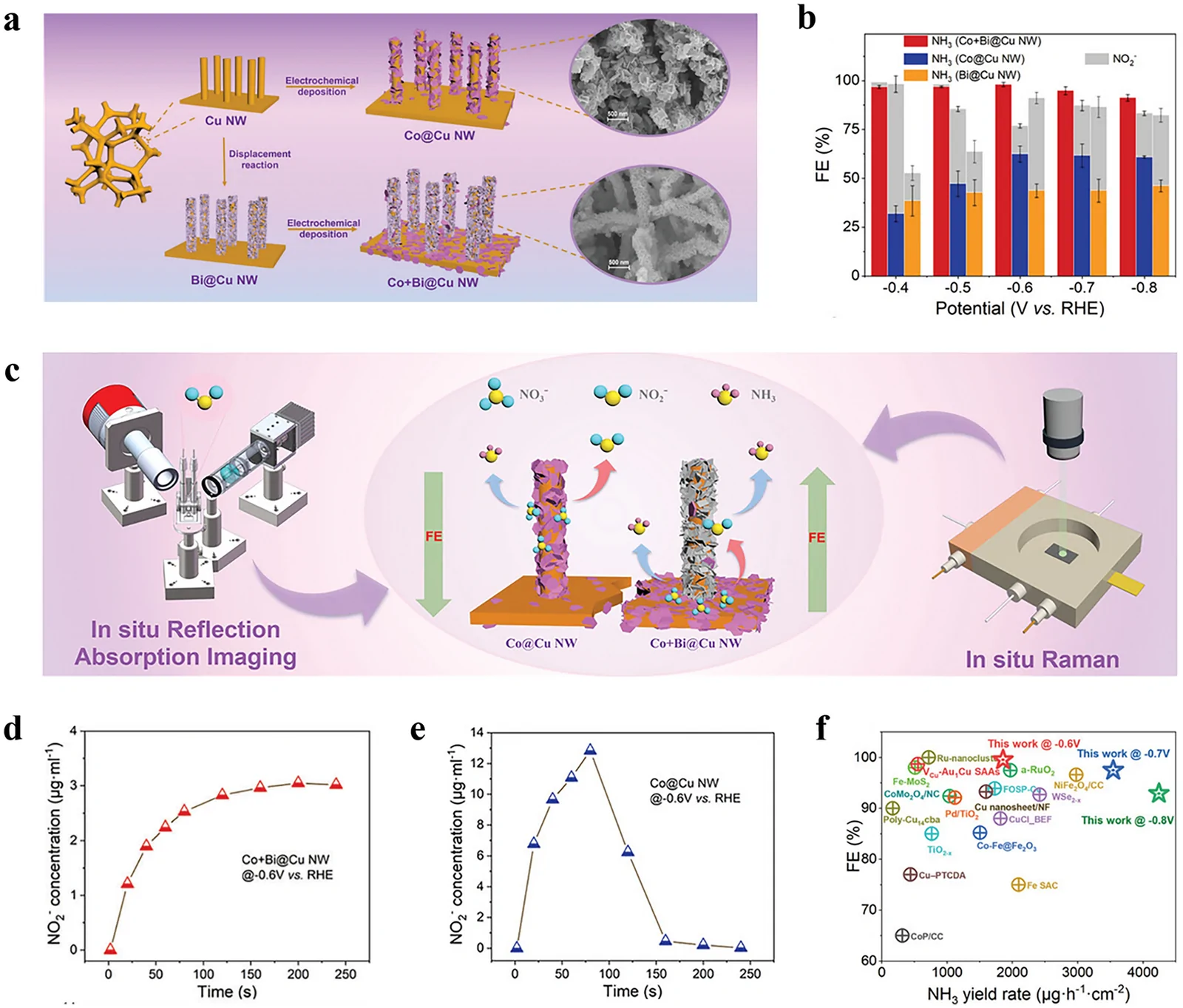
**a** Schematic diagram of preparation process and corresponding SEM images [49]. Copyright 2023, Wiley–VCH. **b** FE of NO_3_RR for samples at different potentials [49]. Copyright 2023, Wiley–VCH. **c** schematic diagram of in situ reflection absorption imaging and in situ Raman spectra, as well as NO_3_RR mechanism on the catalysts [49]. Copyright 2023, Wiley–VCH. **d, e** Curves of NO_2_^−^ concentration on Co + Bi@Cu NW and Co@Cu NW surface changing with time measured by in situ reflection absorption imaging [49]. Copyright 2023, Wiley–VCH. **f** Comparison of the NO_3_RR performance of the Co + Bi@Cu NW (This work) with other catalysts reported in the literatures [49]. Copyright 2023, Wiley–VCH

Similarly, Zhao et al. designed a Bi–Co corridor-like structure by exploiting the limited electron accessibility of Bi element to deposit Co to the bottom of the catalyst (Co + Bi@Cu NW) [49]. As shown in Fig. 5a, the Bi–Co corridor catalyst has a three-dimensional structure with Bi nanoparticles in the outer layer and Co hexagonal sheets in the inner layer, which showed a high FE toward NH_3_ (Fig. 5b). They found that the NO_2_^−^ concentration on the surface of Co + Bi@Cu NW was low during the process of NO_3_RR by means of in situ reflection absorption imaging (Fig. 5c, left) and showed a trend of firstly increasing followed by stabilizing, as shown in Fig. 5d. Differently, Co@Cu NW showed a rapid change in NO_2_^−^ concentration, and reached the highest surface concentration then began to decrease rapidly (Fig. 5e). It was inferred that the catalytic reaction on Co@Cu NW is forced to shift to NO_2_RR from NO_3_RR with increasing NO_2_^−^ concentration, while Co + Bi@Cu NW could actively consume NO_2_^−^ at lower concentrations. These in situ reflectance absorption imaging tests provided Bi in Co + Bi@Cu NW has a strong trapping effect on produced NO_2_^−^ during NO_3_RR, further demonstrating the successful implementation of the strategy that the Bi–Co corridor structure could enhance selective NH_3_ production. Besides, the intermediates *NOH and *NH of the NO_3_RR process of Co + Bi@Cu NW were found by in situ Raman spectroscopy (Fig. 5c, right), and the reaction path of the catalyst was determined to be the NOH path. Density functional theory calculations also showed that a small amount of Co loaded onto Bi could facilitate the rapid conversion of intermediate *NO_2_ in the NO_3_RR process and synergistically improve the FE of NH_3_ production. As a result, the authors inferred that NO_3_^−^ was preferentially adsorbed on the Co site at the bottom of the catalyst for the reaction, and part of the escaped NO_2_^−^ will be captured and converted into NH_3_ by the outer Bi, finally showing a FE of nearly 100% (Fig. 5b). Figure 5f compares NO_3_RR performance between Co + Bi@Cu NW and other catalysts reported in the literature, exhibiting the advantage of this unique structure design.

Beside the composition with carbon, metal oxides can also improve the performance of metal Co. Fan et al. constructed a Schottky heterostructure that coupled metallic Co with semiconductor TiO_2_ [64]. The built-in electric field in the heterostructure could accelerate the rate determining step and facilitate NO_3_^−^ adsorption, ensuring the selective conversion to NH_3_. Expectantly, the Co@TiO_2_ electrocatalyst attained an excellent FE of 96.7% and a high NH_3_ yield of 800 µmol h^−1^ cm^−2^ under neutral solution, as well as remarkable stability in 50-h electrolysis test.

### Cobalt Alloys (Coordination with Heterometal)

Coordination with heterometal atoms can alter the electronic structure and surface strain of cobalt, shifting the d-band center, optimizing adsorption/desorption energy and eventually improving catalyst performance [66–68]. The introducing of heterometal atoms can be easily achieved by alloying technique, and a facilitated electron transfer can be realized during the electrochemical processes. In fact, numerous Co-based alloys have been reported for electrocatalytic NO_3_RR, and showed suitable performances with a lower cost than metallic cobalt. For example, a Cu_2.5_Co alloy designed by Chen et al. achieved a remarkable ammonia yield of 164.23 μmol h^−1^ cm^−2^ at − 0.25 V vs. RHE and a FE of 96.29% at − 0.05 V vs. RHE in a neutral solution [69]. The electronic regulation of Co on Cu reduced the Tafel slope from 182 mV dec^−1^ of monometallic Cu to 108 mV dec^−1^ of bimetal Cu_2.5_Co alloy; the addition of Co optimized the overall catalytic activity of the catalyst. In another case, He et al. designed an alloy nanostructure of sub-20 nm thick nanoribbons, which were assembled by sub-5 nm Cu/Co nanophases [70]. Theoretical and experimental studies showed that the Cu/Co nanophases could be rapidly activated and subsequently stabilized by a specifically designed redox polymer (> − 0.1 V vs. RHE), thus enabling the formation of exposed binary phase boundaries for enhanced NO_3_^−^ adsorption, and the adjacent Cu/Co sites at sub-5 nm distance could act as efficient tandem catalysis for NO_3_^−^ to NH_3_. As a result, this Cu–Co nanoribbon attained a high FE of 93%–98% in 1–100 mM NO_3_^−^ at − 0.1 V vs RHE and the highest EE of ~ 42%. Furthermore, the covering layer of protective redox polymer has cationic viologen moieties for efficient transport of NO_3_^−^ anions and dynamically reduces the released NO_2_ by its reversible redox behavior. Hence, a high FE of > 97% for NH_3_ is maintained up to industry level current densities of ~ 450 mA cm^−2^ at − 0.1 V vs RHE, opening up new possibilities for designing advanced NO_3_RR catalysts. Jeon et al. reported a catalyst of cobalt–copper (Co_1−x_Cu_x_) nanoparticles supported on carbon fiber paper [71]. They found that the optimized Co_0.5_Cu_0.5_ catalyst showed a high NH_3_ FE of over 95% at − 0.03 V, with NH_3_ partial current density of ~176 mA cm^−2^ at 50 mM nitrate, which was 7.3- and 1.7-fold higher than that of its pure Co and Cu counterparts. And a stability test over 12 cycles confirmed the long-term operation of this catalyst. The authors suggested that replacing of Co with parts of Cu enabled the tuning of onset potential on Co catalyst while maintaining a high selectivity toward NH_3_.

Recently, a CuCo alloy composited with conductive metal–organic frameworks (HHTP) was prepared and exhibited outstanding electrocatalytic activity with a high NH_3_ yield rate of 299.9 μmol h^−1^ cm^−2^ and a large Faradic efficiency of 96.4% [72]. Both theoretical and experimental results revealed that the Co sites could affect the electron structure of Cu sites in Cu1Co1 HHTP slab and decrease the ΔG of the potential determining step in the NO_3_RR process. Moreover, the Co sites brought a higher selectivity to Cu active sites for reducing *NO_2_ to *NO, rather than the desorption of NO_2_^−^.

**Fig. 6:**
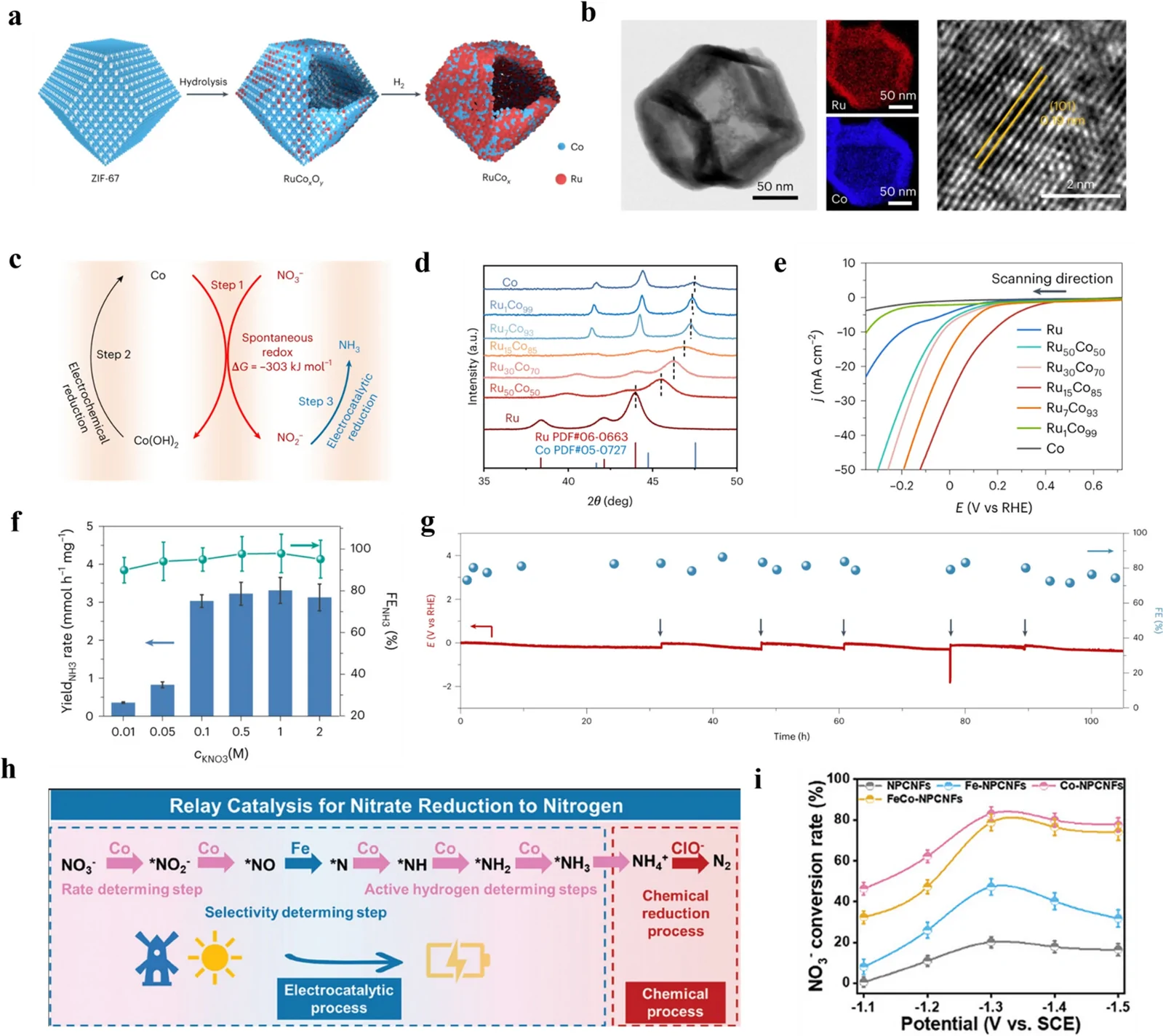
**a** Schematic of Ru_x_Co_y_ HND synthesis [72]. Copyright 2023, Springer Nature. **b** TEM, corresponding EDS mapping and HRTEM images of Ru_15_Co_85_ HNDs [72]. Copyright 2023, Springer Nature. **c** The three-step relay mechanism for the NO_3_RR [72]. Copyright 2023, Springer Nature. **d** Locally enlarged XRD patterns of Ru_x_Co_y_ HNDs [72]. Copyright 2023, Springer Nature. **e** LSV curves for the NO_3_RR over Ru_x_Co_y_ HNDs under 1,600 r min^−1^ with 80% iR correction [72]. Copyright 2023, Springer Nature. **f** NO_3_RR performance over Ru_15_Co_85_ HNDs at different concentrations of KNO_3_, Error bars correspond to the standard deviations of three independent measurements, and the center value for the error bars is the average of the three independent measurements [72] Copyright 2023, Springer Nature. **g** Long-term electrocatalytic stability test of the NO_3_RR (0.1 M KNO_3_ + 0.1 M KOH) over Ru_15_Co_85_ HNDs at 200 mA cm^−2^ using a continuous-flow system in an H-cell. Black arrows indicate the renewal of fresh electrolytes [72]. Copyright 2023, Springer Nature. **h** Schematic of the electrocatalytic NO_3_RR to N_2_ under ambient conditions with renewable energy [73]. Copyright 2024, Wiley–VCH. **i** NO_3_^−^ conversion rate of FeCo-NPCNFs and other contrast samples at various potentials [73]. Copyright 2024, Wiley–VCH

Zhang’s group designed a Ru_x_Co_y_ hollow nano-dodecahedron alloy catalyst (Ru_x_Co_y_ HNDs, Fig. 6a, b) and proposed a three-step relay mechanism, namely spontaneous redox reaction, electrochemical reduction, and electrocatalytic reduction (Fig. 6c) [72]. As Co can undergo a spontaneous redox reaction with NO_3_^−^ to produce Co(OH)_2_ and NO_2_^−^ with a Gibbs free energy change of − 303.01 kJ mol^−1^ (step 1). Then, Co(OH)_2_ and NO_2_^−^ can be reduced into Co and NH_3_ through electrochemical and electrocatalytic processes, respectively. According to the classic scaling relation, the adsorption energy of a hydrogen atom can be regarded as the descriptor for active hydrogen formation. Ru possess moderate adsorption energies for hydrogen atoms and is low cost among the noble metals. Thus, the Ru was chosen to alloy with Co alloy in this work. Figure 6d shows the locally enlarged XRD patterns of this synthesized Ru_x_Co_y_ HNDs. Moreover, they found that Ru_15_Co_85_ could exhibit an onset potential of + 0.4 V vs. RHE (Fig. 6e), and a NH_3_ yield as high as 3.2 ± 0.17 mol g_cat_^−1^ h^−1^ was obtained under + 0 V vs. RHE, with a corresponding FE of 97% ± 5%, and an EE of 42% ± 2% at + 0.3 V vs. RHE for NH_3_ synthesis, as shown in Fig. 6e, g. In another case, Yang’s group reported a tactic to raise rate of NO_3_RR and increase selectivity to N_2_ using bimetal catalyst of FeCo: Co is inclined to act on the key steps needed in NO_3_RR process, rate-determining step (RDS: *NO_3_ to *NO_2_) and the subsequent *N hydrogenation, while Fe exhibits the efficient activity for the selectivity-determining step (SDS: *NO to *N then to N_2_), thus a relay catalysis mechanism could be achieved (Fig. 6h) [73]. In addition, in order to tune the electronic properties of the electrocatalysts, they combined confinement engineering and alloying strategies and prepared 0 D FeCo alloy nanoparticles confined in nitrogen-doped porous carbon nanofibers (FeCo–NPCNFs) via an electrospinning method. These alloy catalysts showed a removal efficiency of 78.5% and an ultra-long cycle stability of 60 cycles (12 h per cycle), as shown in Fig. 6i. DFT calculations unveiled that the introduction of Co active site not only regulates the d-band center of FeCo alloy, optimizes the adsorption of intermediates, but also had a strong capacity to supply active hydrogen species.

As discussed in Sect. 3.1, the Co nanostructure seems to exhibit poor HER activity, inhibiting the NH_3_ production. However, the coordination with heterometal atoms can effectively save this problem. For instance, noble metals such as Ru, Rh, Pd, Ir, and Pt, which possess moderate adsorption energies for hydrogen atoms, can be used for alloying with Co, benefiting the NH_3_ production of Co-based materials. While the transition metals such as Cu and Fe, shows a limited supply of active hydrogen species, and the FeCo alloy tends to produce N_2_ rather than NH_3_ during the NO_3_RR.

### Cobalt Compounds (Coordination with Heterometal Oxygen)

Compared with metal atoms, coordination with non-metal atoms can also alter the electronic structure of cobalt more extensively due to their electronegativity. The most common non-metal coordination in natural environment are the cobalt-based compounds, including cobalt oxides, cobalt phosphides, and cobalt borides. The strategic incorporation of non-metallic elements (e.g., O, P, B) into these compounds enables precise tuning of their electronic structures, which significantly enhances both catalytic stability and NO_3_RR performance. This electronic modulation manifests through several key mechanisms: (1) optimization of charge distribution across the catalyst surface, (2) modification of metal–ligand bond strengths, and (3) creation of highly active catalytic sites with tailored adsorption properties. These structural and electronic modifications collectively contribute to the development of robust, high performance catalysts that exhibit superior activity, selectivity, and durability in NO_3_RR applications.

#### Coordination with Oxygen

The most common O coordination catalyst is cobalt oxide, including CoO, Co_2_O_3_, and Co_3_O_4_, exhibiting distinct properties and applications. CoO is a black, magnetic, semiconductor powder used in catalysis, magnetic materials, and batteries [74–76]. Co_2_O_3_, a red powder with promising catalytic and electrochemical properties, has received limited attention in electrocatalysis but holds potential for future exploration [26]. Among these, Co_3_O_4_ stands out due to its black color, excellent catalytic, electrochemical, and magnetic properties, making it suitable for applications in catalysis, batteries, magnetic materials, and gas sensors [20, 65, 77–83]. Spinel Co_3_O_4_ is particularly promising as an electrocatalyst for NO_3_RR due to its high redox capability, cost-effectiveness, and stability in alkaline environments. It has been widely used in electrocatalysis, such as ORR, HER, and NRR [20, 77, 79]. Notably, Co_3_O_4_, including pure forms, composites, and heteroatom-doped versions, is the most commonly reported cobalt-based material for electrocatalytic NO_3_RR and will be discussed further in detail.

Spinel cobalt oxide (Co_3_O_4_) exhibits a p-type semiconducting property and is consisted of mixed valence oxide of CoO and Co_2_O_3_. In the structure of Co_3_O_4_, the tetrahedral 8(a) sites and the octahedral 16(d) sites are occupied by Co(II) and Co(III) ions, respectively [77]. Co_3_O_4_ possessing the merits of earth abundance, low cost, good environmental compatibility, attractive electrocatalytic activity, as well as the electrochemical durability [77, 78].Due to the high activity of Co_3_O_4_ toward electrocatalysis, pure Co_3_O_4_ may be an ideal candidate for electrocatalytic NO_3_RR. At first (2017), a Co_3_O_4_ film coated on Ti substrate was prepared by a sol–gel method and applied as a cathode catalyst for electrochemical reduction NO_3_^−^ [66]. As expected, this Co_3_O_4_/Ti catalyst exhibited quite outstanding ability for converting NO_3_^−^ to NH_3_. More importantly, the authors also prepared some other common cathodes at that time (Ti, Cu and Fe_2_O_3_/Ti), by contrast, Co_3_O_4_/Ti exhibited even better NO_3_^−^ removal and NH_3_ generation efficiencies. Even though this work focused more on polluted water treatment and lacked normalized or comparable data with respect to ammonia production, it demonstrated huge potential for the application of Co_3_O_4_ for electrocatalytic NO_3_RR. After this study, a Co_3_O_4_ nanosheet structured film was synthesized on Ti substrate through the electrodeposition method by Jia’s group [79]. Due to the large surface area and highly efficient electron transfer, this unique nanostructure showed better electrochemical NO_3_^−^ removal performance than Co_3_O_4_ solid particle cathode. In addition, the effects of reaction temperature were investigated, which showed that NO_3_^−^ removal rate was enhanced with reaction temperature rising from 5 to 45 °C. Interestingly, the physical properties of Co_3_O_4_ nanosheet cathode were more stable at high reaction temperature. Low reaction temperature could destroy the nanosheet structure, and obviously reduce the ratio of Co^2+^/Co^3+^, but significantly increase the ratio of oxygen vacancy/lattice oxygen, thus leading to inferior performance.

To enhance the electrocatalytic activity by controlling the crystal facet exposure, a facile hydrothermal method was used by Li’s group, during which different crystal facet exposure and Co^2+^/Co^3+^ ratios could be obtained by adjusting hydrothermal pressure and duration time [81]. The authors found that low pressure and a short hydrothermal duration time tended to obtain rod-like film with almost pure (220) facet exposure, while sheet-like film with a mixture of (220) and (222) facets was formed with relatively high pressure. Besides, the Co^2+^/Co^3+^ ratio was 2.067 for Co_3_O_4_ rods and 1.717 for Co_3_O_4_ sheets, which possibly resulted from the doping of CO_3_^2−^/OH^−^, and the introduction of oxygen vacancies. The larger exposure of (220) facet and the more amount of Co^2+^ and oxygen vacancies result in the better NO_3_^−^ removal and NH_4_^+^ conversion kinetics of Co_3_O_4_ rods film than that of Co_3_O_4_ sheets, with a NO_3_^−^–N removal rate of 0.00907 and 0.00641 min^−1^, respectively, at a current of 50 mA. The rational design and synthesis of kinds of nanostructures with controlled spatial architecture is also significant in electrocatalysis owing to their abundant active sites and the expedited electron/mass transfer. For example, Fu et al. designed and prepared a 3D Co_3_O_4_ nanostructure on carbon felt (CF) by a simple electrodeposition method with subsequent calcination treatment. This nanostructure could deliver a high reaction rate for the NO_3_RR, with NO_3_^−^ removal of 91.09% within 90 min at − 1.3 V vs. Ag/AgCl, and exhibited excellent stability with a decrease of 6.4% after 10 cycles [37]. Density functional theory calculations, electron spin resonance analysis, and cyclic voltammetry results proved that atomic H* (indirect pathway) played a prominent role in NO_3_RR and clarified the synergistic effect of Co(III) and Co(II) in the Co(II)–Co(III)–Co(II) redox cycle during the reaction: Co(III) preferred the adsorption of NO_3_^−^ and Co(II) favored the production of H*. In addition, they found that the sample with the 1.3 Co(II)/Co(III) ratio performed best, with a lower energy barrier for H^∗^ formation of only 0.46 eV than other ratios. This study suggested that building 3D structure and optimizing Co(II)/Co(III) ratio were important for designing an efficient Co_3_O_4_ electrocatalyst for NO_3_RR. Although Co_3_O_4_-based materials are promising electrocatalysts for conversion of nitrate to ammonia due to low-cost and high selectivity, their activity is still unsatisfactory and the genuine active site is unclear. Very recently, Hu et al. discovered that the NO_3_RR activity of Co_3_O_4_ was highly dependent on the geometric location of the Co site: the reduction process tended to occur at octahedral Co (Co_Oh_) rather than tetrahedral Co (Co_Td_) sites [77]. And Co_Oh_O_6_ could be electrochemically transformed to Co_Oh_O_5_ along with the formation of O vacancies (O_v_) during the reduction process. Tt was also proved that this in situ generated Co_Oh_O_5_–O_v_ configuration was the genuine active site for the NO_3_RR. The replacement of inert Co_Td_ with different contents of Cu^2+^ cations could enhance the activity of Co_Oh_ sites. For example, (Cu_0.6_Co_0.4_) Co_2_O_4_ with optimized Co_Oh_ sites achieved a maximum NH_3_ FE of 96.5% with an ultrahigh NH_3_ rate of 1.09 mmol h^−1^ cm^−2^ at − 0.45 V vs. RHE, outperforming most reported non-precious metal-based electrocatalysts. This interesting work tells us that tuning local electronic structures of Co_Oh_ sites can effectively boost the NO_3_RR activity of Co_3_O_4_-based materials.

By combining Co_3_O_4_ with other metals, metal oxides, or non-metal materials, various electrocatalysts can be designed, offering promising research potential and enhanced NO_3_RR performance through synergistic effects or the creation of heterostructures. Moreover, the firmly neighboring hetero-structures provide a controllable coordination environment around Co sites. The careful design of the nanostructure and composition often results in more abundant active sites and accelerated electron/mass transfer. In this section, we will discuss strategies for composing Co_3_O_4_ with other materials to achieve high-performance NO_3_RR electrocatalysts.

**Fig. 7:**
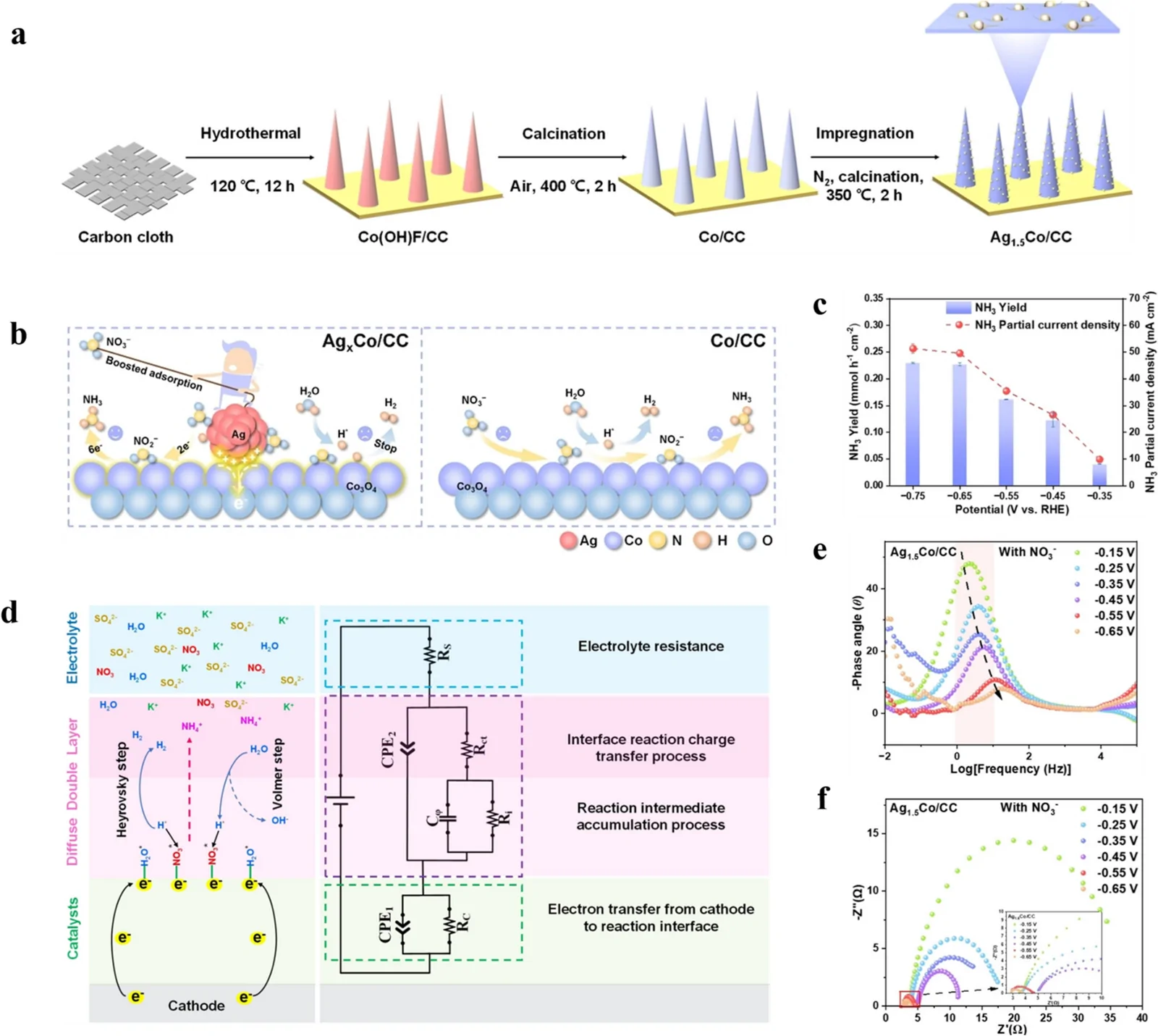
**a** A Scheme for catalysts synthesis of Ag-modified Co_3_O_4_ [82]. Copyright 2024, Wiley–VCH. **b** Schematic illustration of the NO_3_RR process over the Ag_x_Co/CC catalyst system [82]. Copyright 2024, Wiley–VCH. **c** Potential-dependent ammonia partial current density and yield rate on Ag_1.5_Co/CC [82]. Copyright 2024, Wiley–VCH. **d** Equivalent circuit model [82]. Copyright 2024, Wiley–VCH. **e** Bode phase plots of Ag_1.5_Co/CC at different potentials in electrolyte of 0.5 M K_2_SO_4_ with NO_3_^−^ [82]. Copyright 2024, Wiley–VCH. **f** Nyquist plots of Ag_1.5_Co/CC at different potentials in 0.5 M K_2_SO_4_ electrolyte with NO_3_^−^ [82]. Copyright 2024, Wiley–VCH

Coupling metal oxides with noble metals has been demonstrated to improve electrochemical catalytic efficiency [83]. Fan et al. designed a high-efficiency electrode of Ag-modified Co_3_O_4_ for NO_3_RR, as shown in Fig. 7a, b [82]. These catalysts exhibited an impressive nitrate conversion rate of 96.86%, ammonia FE of 96.11%, and ammonia selectivity of ~ 100% (Fig. 7c). Notably, the intrinsic activity of Ag_1.5_Co/CC is ~ 81 times that of Ag nanoparticles (Ag/CC). Multiple characterizations and theoretical computations confirmed the presence of interfacial electronic interactions (IEIs) between Ag and Co_3_O_4_, which stabilized the CoO_6_ octahedrons within Co_3_O_4_ and significantly promoted the adsorption of reactants (NO_3_*) as well as intermediates (NO_2_* and NO*), while suppressing the Heyrovsky step, thereby improving nitrate electroreduction efficiency, as shown in Fig. 7d–f [82]. Furthermore, these findings revealed a synergistic effect between different active sites that enables tandem catalysis for NO_3_RR: NO_3_* reduction to NO_2_* predominantly occurred at Ag sites while NO_2_* tended to hydrogenate to ammonia at Co sites. This study offered valuable insights for the development of high-performance NO_3_RR electrocatalysts.

Zhang et al. designed an Au/Co_3_O_4_ composite for the purpose of using Au species to tune the surface structure of Co_3_O_4_ and improve the efficiency of NO_3_RR to NH_3_ [84]. The obtained Au nanocrystals-Co_3_O_4_ catalyst exhibited an onset potential of 0.54 V vs. RHE, NH_3_ yield rate of 27.86 µg h^−1^ cm^−2^, and FE of 83.1% at 0.437 V vs. RHE in an H-cell, which was much higher than Au small species (Au clusters or single atoms)-Co_3_O_4_ (15.12 µg h^−1^ cm^−2^) and pure Co_3_O_4_ (11.38 µg h^−1^ cm^−2^), respectively. Combined experiments with theory calculations, they attributed the enhanced performance of Au nanocrystals-Co_3_O_4_ to the reduced energy barrier of *NO hydrogenate-on to the *NHO and suppression of HER, which originated from the charge transfer from Au to Co_3_O_4_. Moreover, a solar cell and an anion exchange membrane electrolyzer (AEM) were used to realize a solar-driven NO_3_RR to NH_3_ prototype, which reached a yield rate of 4.65 mg h^−1^ and FE of 92.1%, showing the potential for practical application. In another case, inspired by the enhancement effect of heterostructure on electrocatalysis, Zhang et al. designed and prepared Au nanowires decorated ultrathin Co_3_O_4_ nanosheets (Co_3_O_4_–NS/Au–NWs) nanohybrids for electrocatalytic NO_3_RR[83]. These nanohybrids had achieved an outstanding catalytic performance with a high NH_3_ yield rate of 2.661 mg h^−1^ mg_cat_^−1^. Theoretical calculation revealed that Au heteroatoms could effectively adjust the electron structure of Co active centers and reduce the energy barrier of the determining step (*NO → *NOH) during NO_3_RR. Besides, due to the localized surface plasmon resonance (LSPR) property of Au–NWs, this heterostructure showed an obviously plasmon-promoted activity for NO_3_RR, leading to an enhanced NH_3_ yield rate of 4.045 mg h^−1^ mg_cat_^−1^.

In addition to noble metals, non-noble metals such as Co and Cu have been utilized in combination with Co_3_O_4_, offering a significant cost advantage compared to noble metals [85–89]. Zhao et al. designed and fabricated a Co_3_O_4_/Co composite by combining electrodeposition and the calcination process [85]. Benefited from the interlaced nanosheets, the modulated oxygen vacancy could be obtained as well as an open-pore and defective structures. Therefore, this Co_3_O_4_/Co catalyst could accumulate more nitrate on activated sites and display excellent NO_3_RR properties in the neutral electrolyte with an ammonia yield rate of 4.43 mg h^−1^ cm^−2^, and a FE of 88.7%. Theoretical calculations revealed that oxygen vacancy could enhance nitrate adsorption energy, suppress the hydrogen evolution reaction, and weaken the rate-determining step of *NO → *HNO. Furthermore, the continuous NO_3_RR flow electrolyzer also demonstrated that this Co_3_O_4_/Co electrocatalyst acted effectively with not only a larger ammonia yield but also long durability, accelerating the electrocatalytic NO_3_RR to commercial application.

**Fig. 8:**
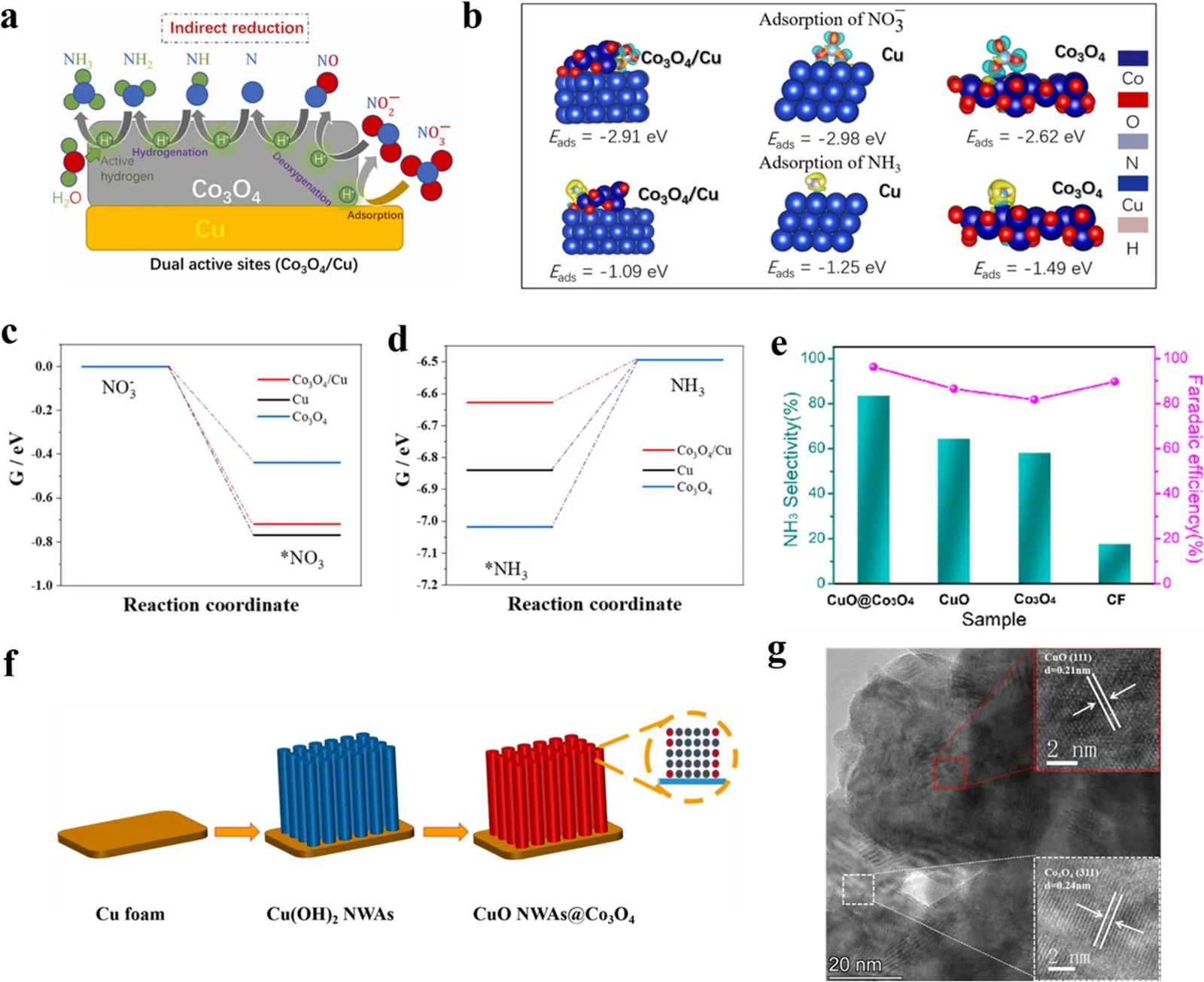
**a** Illustration of electrocatalytic mechanism of Co_3_O_4_/Cu dual active sites electrode for NO_3_RR. Theoretical insight of dual active sites for NO_3_RR [89]. Copyright 2021, Elsevier. **b** Adsorption structure with adsorption energy of adsorbing nitrate and ammonia on Co_3_O_4_/Cu, Cu and Co_3_O_4_ model, respectively [89]. Copyright 2021, Elsevier. **c, d** Gibbs free energy for nitrate of adsorption and ammonia of desorption on Co_3_O_4_/Cu, Cu and Co_3_O_4_ model, respectively [89]. Copyright 2021, Elsevier. **e** Nitrate rate of conversion and ammonia yield rate over CuO@Co_3_O_4_, CuO, Co_3_O_4_, Cu foam [87]. Copyright 2022, Elsevier. **f** Schematic illustration of the formation mechanism [87]. Copyright 2022, Elsevier. **g** TEM images of CuO NWAs@Co_3_O_4_ [87]. Copyright 2022, Elsevier

In order to solve the problem that strong adsorption of the target product may cause high activation energy for product desorption in scaling relations, Fu et al. proposed a strategy of dual active sites (Cu and Co_3_O_4_), as shown in Fig. 8a, which could achieve strong adsorption of nitrate (–2.91 eV) with low desorbing energy barrier of ammonia (0.13 eV) according to the DFT calculations (Fig. 8b–d) [89]. Experimental results manifested that this dual active site strategy was effective, and the Co_3_O_4_/Cu electrode achieved a high ammonia yield rate of 684 µg mg_cat_^−1^ h^−1^ with 94.6% faradic efficiency, surpassing single active site Co_3_O_4_ and Cu electrodes. In addition, in situ electrochemical characterization detected the vital intermediates *NH and *NH_2_, demonstrating that indirect reduction was the main pathway on the Co_3_O_4_/Cu electrode.

Beside these metallic materials, composition with metal oxides seems more common; for example, Wang et al. reported a catalyst of Co_3_O_4_@NiO hierarchical nanotubes (Co_3_O_4_@NiO HNTs) with NiO porous nanosheets assembled on Co_3_O_4_ nanotubes [86]. The as-obtained catalyst exhibited an outstanding performance for the cathodic nitrate electroreduction to ammonia reaction with a yield rate of 6.93 mmol h^−1^ g^−1^, a FE of 54.97%, as well as a robust stability. Similarly, a core–shell heterostructure that Co_3_O_4_ anchored on CuO nanowire arrays (CuO NWAs@Co_3_O_4_) was also prepared for NO_3_RR, as shown in Fig. 8f, g [87]. At − 0.23 V vs RHE, the NH_3_ yield rate of the CuO@Co_3_O_4_ reached 1.915 mmol h^−1^ cm^−2^, much higher than those of CuO nanowire arrays (1.472 mmol h^−1^ cm^−2^), and Co_3_O_4_ (1.222 mmol h^−1^ cm^−2^), even higher than the reported Cu-based catalysts at that time, as shown in Fig. 8e. The authors proposed that the synergetic effects of the heterostructure combining atom hydrogen adsorption and NO_3_RR led to enhanced NO_3_RR performance. Gao et al. prepared a Co_3_O_4_–TiO_2_/Ti cathode via a solgel method, during which the dispersion of the Co_3_O_4_ catalyst particles could be improved by the addition of PVP to the coating liquid [88]. The presence of anatase not only benefited the preparation process, which effectively stabilized Co_3_O_4_ and prevented the release of toxic Co ions into the solution, but also optimized the performance of NO_3_RR. They found that the as prepared Co_3_O_4_–TiO_2_/Ti cathode with calcination at 500 °C was negligibly impacted by solution pH in the range of 3.0–9.0, and ammonium ions were the main final NO_3_RR product. The electrochemical analyses, scavenging experiments, and DFT calculations collectively confirmed that NO_3_RR was mainly induced by the Co^2+^–Co^3+^–Co^2+^ redox process instead of being directly resulted from the electrons generated at the cathode.

Unlike noble metal (e.g., Pd and Ag)-based catalytic reaction systems, in the present Co_3_O_4_ mediated electrocatalytic reaction process, atomic H* would more favorably turn to H_2_ by Heyrovsky and Tafel routes and therefore contribute marginally to the NO_3_RR. Yang et al. introduced CuO and Co_3_O_4_ to the surface of Ti via a coating-calcination method and successfully prepared a CuO–Co_3_O_4_/Ti composite [90]. The CuO–Co_3_O_4_/Ti cathode coupled with an anode in a reaction cell could eventually remove NO_3_^−^ with the main products of N_2_, NH_4_^+^, and NO_2_^−^, delivering an enhancement of approximately 20% higher than the Co_3_O_4_/Ti electrode. Moreover, the presence of Cl^−^ could greatly promote the removal efficiency of NO_3_^−^ from 40.1% to 94.0%, resulting from the NH_4_^+^ oxidation with free chlorine produced from the anode.

Furthermore, CuO–Co_3_O_4_/Ti was applied to three types of actual wastewater to test the practical activity of CuO–Co_3_O_4_/Ti catalyst for nitrate removal, including biological effluent of municipal wastewater, industrial wastewater, and a regeneration concentrate from an anion exchange process, which showed the feasibility of the electrode to the water with high conductivity and high Cl^−^ concentration and potential for practical application.

Recently, designing and preparing tandem catalysts have drawn much attention. In order to understand the detailed reaction mechanism and improve the performance of Co_3_O_4_ for NO_3_RR, Zhang et al. reported a Cu_2_O + Co_3_O_4_ tandem catalyst by physically mixing of Cu_2_O and Co_3_O_4_ nanocubes [26]. This tandem catalyst showed a superior 85.4% FE of NH_3_ formation and a high NH_3_ yield rate of 12.76 mg h^−1^ cm^−2^ at − 0.3 V vs. RHE, which exceeded that of Co_3_O_4_ by ~ 2.7-fold and that of Cu_2_O by ~ 7.5-fold, respectively. Interestingly, they also established a carbon nanoelectrode (CNE) platform that allowed a single entity of Cu_2_O + Co_3_O_4_ nanocubes with two particles relative to each other. They confirmed the sequential tandem catalysis and monitored the structural and phase evolution caused by tandem catalysis during the reaction, which gives an important inspire for further design of a Co_3_O_4_-based tandem catalyst for NO_3_RR. Additionally, He et al. synthesized a tandem catalyst consisting of core–shell Cu/CuOx and Co/CoO phases [114]. Electrochemical evaluation, kinetic studies, and in situ Raman spectra revealed that the inner Cu/CuOx phases preferentially catalyzed NO_3_RR to NO_2_^−^, which was rapidly reduced to NH_3_ at the nearby Co/CoO shell. This unique tandem catalyst system leaded to a NO_3_^−^-to-NH_3_ FE of 93.3% ± 2.1% in a wide range of NO_3_^−^ concentrations at pH 13, and a high NH_3_ yield rate of 1.17 mmol cm^−2^ h^−1^ in 0.1 M NO_3_^−^ at − 0.175 V vs. RHE, demonstrating the advantages of composition strategy.

Other cobalt oxides, such as CoO, have also been applied for electrocatalytic NO_3_RR. Wang et al. designed and prepared a non-proportional cobalt oxide of ultrathin CoO_x_ nanosheets with abundant surface oxygen, which delivered an ultrahigh ammonia yield of 82.4 ± 4.8 mg h^−1^ mg_cat_^−1^ with a FE of 93.4% ± 3.8% at − 0.3 V vs. RHE [92]. Theoretical calculation revealed that the surface oxygen on cobalt sites could stabilize the adsorbed hydrogen on cobalt oxide, which hampered the evolution of hydrogen and led to enhanced NO_3_RR activity. This work demonstrated that surface modification played a critical role in suppressing the HER and facilitating the NO_3_RR through a *NHO pathway with a lower energy barrier. Recently, a high-efficiency electrocatalyst of CoO (111) nanowire arrays grown on titanium mesh (CoO NWA/TM) was successfully prepared and reached a remarkable NH_3_ yield of 6.8 mg h^−1^ cm^−2^ and a high FE of 95.1%, along with excellent electrochemical durability [93]. These works demonstrate the great potential of non-Co_3_O_4_-based cobalt oxides for NO_3_RR, nevertheless, it seems more effort should be devoted to this research direction.

The structural and electronic versatility of cobalt oxides has driven significant advancements in nitrate electroreduction. Through atomic-scale engineering strategies such as heteroatom doping and vacancy control, materials ranging from defect-rich Co_3_O_4_ to metastable CoO_x_ demonstrate broad performance metrics, achieving ammonia FE between 54% and 99.5% across varying pH conditions, as critically compared in Table 2. Notably, Mn-doped Co_3_O_4_ nanotubes exhibit a 20-fold activity enhancement over pristine counterparts, highlighting the transformative potential of targeted electronic modulation. These oxide-based systems not only establish performance benchmarks but also provide foundational insights into active site design. Such insights prove equally vital for understanding subsequent material classes, including cobalt phosphides and borides, which leverage distinct coordination environments to further optimize catalytic behavior.

The incorporation of an additional metal element enhances electrical conductivity compared to Co-based binary materials . Furthermore, these metal elements act as reductants that decompose NO_3_^−^ to NH_3_ or N_2_. Heteroatom doping can enrich the coordination environment beyond O, and is an effective method to boost the electrocatalytic activity of materials with different dimensions. Jin et al. employed a mechanochemical strategy to precisely anchor Ir single-atom catalysts (SACs) or nanoclusters onto spinel Co_3_O_4_, and gained insights into the mechanisms of NO_3_RR at an atomic level [94]. Compared with the conventional pyrolysis method, the established mechanochemical strategy could realize ultrafast (10 min) and mild synthesis at ambient temperature, avoiding hard-to-control agglomeration at high pyrolysis temperature. The results revealed that atomic-scale Ir SAC–Co_3_O_4_ could significantly boost NO_3_RR activity compared with nanocluster-scale Ir NC–Co_3_O_4_ by effectively enhancing charge transfer and decreasing the energy barrier. This study not only successfully fabricates nano-catalysts with controllable size, and further gives new insights into the NO_3_RR mechanism on the basis of the size effect.

Wei et al. reported that Fe-doped Co_3_O_4_ nanoarray could efficiently catalyze NO_3_RR for NH_3_ production in neutral conditions, which achieved a large NH_3_ yield of 0.624 mg h^−1^ mg_cat_^−1^ and high FE of 95.5% at − 0.7 V vs. RHE, as well as quite good stability [91]. Density functional theory calculations revealed that the Fe doping favored the adsorption of NO_3_^−^ on Co_3_O_4_, thus being conducive to NH_3_ production in the NO_3_RR process. Recently, Liu et al. found that the incorporation of manganese (Mn) into the Co_3_O_4_ lattice could achieve high activity and selectivity for NO_3_RR [95]. In more detail, the Mn-incorporated Co_3_O_4_ nanotubes show a high ammonia yield rate of 35 mg h^−1^ cm^−2^ and a FE for ammonia up to 99.5% in neutral media, which are significantly higher than those of transition-metal oxides. Calculations further revealed that the replacement of Co by Mn could reduce the limiting potential of NO_3_RR. Further experimental and calculational results revealed that the Mn ion could easily replace Co in the CoO_6_ octahedron of spinel Co_3_O_4_ partially, which favored to suppress the HER and tuned the adsorption behavior of intermediates, thus boosting the NO_3_RR activity and selectivity. This work successfully provided an insightful understanding on the catalytic origin and demonstrated that incorporation is an effective way to engineer the spinel oxides for enhanced activity and selectivity toward ammonia production in NO_3_RR.

#### Co-coordination with Metallic and Oxygen (Co–O–M)

Besides the incorporation of an additional metal element, this configuration (Co–O–M) exemplifies heterometallic synergy in oxygen-bridged systems, taking Co-based spinel catalysts (e.g., ZnCo_2_O_4_, CuCo_2_O_4_, and Co_2_AlO_4_) as samples, the Co–O–M motif creates dual-metal active sites where M modulates the electronic structure of Co centers via bridging O atoms and eventually enhances NO_3_^−^ to NH_3_ conversion efficiency [95–100]. In these material phases, one third of Co atoms are be replaced by Zn, Cu or Al, which helps reduce the cost of the electrocatalyst due to the abundance of these non-noble metals in the earth's crust compared with Co. Therefore, exploring the use of these Co-based ternary materials as catalysts for NH_3_ electrosynthesis is scientifically and technologically significant. Huang et al. developed a three-dimensional (3D) flower-like zinc cobaltite (ZnCo_2_O_4_) electrocatalyst to convert nitrate into ammonia [99]. The NH_3_ yield rate could reach up to around 2100 µg mg^−1^ h^−1^ at a potential of − 0.6 V vs. RHE, which was around 2.0 times higher than that of pristine Co_3_O_4_. The NH_3_ FE could reach around 95.4% at potential of − 0.4 V vs. RHE with good structural and morphological stability. The authors found that the improved activity of electrocatalytic NO_3_RR could be attributed to the existence of abundant active sites and the charge transfer from Co atoms to Zn atoms after Zn doping. In another case, ZnCo_2_O_4_ nanosheet arrays supported on carbon cloth (ZnCo_2_O_4_ NSA/CC) were designed as a superb 3D electrocatalyst for the highly active and selective conversion of NO_3_^−^ to NH_3_ [100]. In 0.1 M NaOH with 0.1 M NaNO_3_, such catalysts attained a superior FE of 98.33% at − 0.6 V vs. RHE and a large NH_3_ yield of 634.74 μmol h^−1^ cm^−2^ at − 0.8 V vs. RHE, as well as an excellent durability. Theoretical calculations revealed that ZnCo_2_O_4_ (311) surface severely inhibited HER and was highly active for NO_3_RR with a potential determining step of 0.29 eV.

**Fig. 9:**
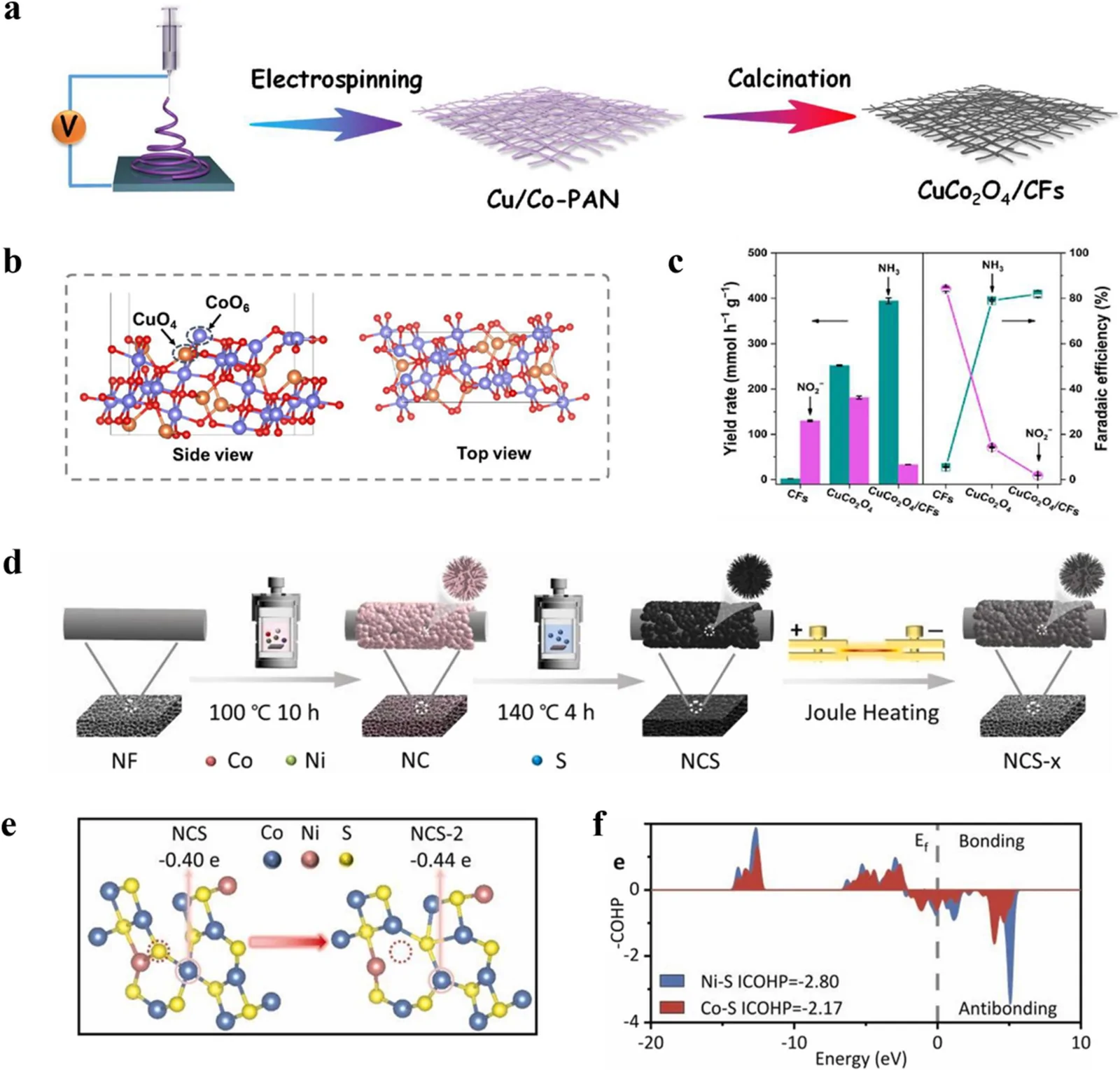
**a** Illustration of the preparation process of CuCo_2_O_4_/CFs [97]. Copyright 2022, Elsevier. **b** Optimized CuCo_2_O_4_ spinel structure model [97]. Copyright 2022, Elsevier. **c** NH_3_ and NO_2_^−^ yield rate and FE of CuCo_2_O_4_/CFs for NO_3_RR over different catalysts with 0.1 M NO_3_^−^ at − 0.3 V vs. RHE [97]. Copyright 2022, Elsevier. **d** Schematic diagram of NCS-x preparation process, where x represents the serial number of samples with different heating temperature [103]. Copyright 2023, Elsevier. **e** Bader charges of Co site in NCS and NCS-2, where NCS-2 represents the sample with heating temperature of 700 °C and performs best for NO_3_RR [103]. Copyright 2023, Elsevier. **f** Crystal orbital Hamilton population (COHP) of Co-S bond and Ni-S bond in NCS [103]. Copyright 2023, Elsevier

As known, Cu exhibits strong NO_3_^−^ absorption and hydrogen evolution reaction (HER) inhibition capabilities during NO_3_RR, but this leads to a lack of *H on the electrode surface [84]. Combining Cu with Co can enhance the *H supply capacity and NH_3_ selectivity in Cu-based catalysts [63]. Niu et al. prepared a porous carbon nanofibers (CFs)-supported CuCo_2_O_4_ electrocatalyst by an electrospun-pyrolysis method (CuCo_2_O_4_/CFs), as shown in Fig. 9a, and the CuCo_2_O_4_ spinel structure model is shown in Fig. 9b [97]. In this composite structure, the CFs was beneficial charge transfer and could inhibit the HER, preferentially catalyzing NO_3_^−^ to NO_2_^−^, while the internally confined CuCo_2_O_4_ nanoparticles could stabilize adsorbed nitrite intermediate (*NO_2_) for further conversion to NH_3_. Benefiting from these, the CuCo_2_O_4_/CFs exhibited a maximum NH_3_ FE of 81.9% at − 0.3 V vs. RHE, with a yield rate of 394.5 mmol h^−1^ g^−1^ at ambient conditions (Fig. 9c). While Deng et al. prepared an electrocatalyst of Co_2_AlO_4_ nanosheet array on carbon cloth (Co_2_AlO_4_/CC) via a simple hydrothermal method [101]. In 0.1 M PBS solution with 0.1 M NO_3_^−^, this catalyst attained an NH_3_ yield of 7.9 mg h^−1^ cm^−2^ with a FE of 92.6%, as well as a high durability. Theoretical calculations revealed that Al ions reduced the electron cloud density on the surface of Co, making it highly conducive to NO_3_^−^ adsorption at Co sites. Additionally, Co_2_AlO_4_ (220) surface inhibited hydrogen evolution reaction but was highly active for NO_3_RR with a low energetically uphill pathway (ΔG of 0.42 eV) for the potential-determining step (*NO_3_ to *NO_3_H), much smaller than that of Co_3_O_4_ (220) (ΔG of 0.83 eV). Additionally, Zhang's group synthesized a plasma-induced defective CoTiO_3−x_ nanofiber with oxygen vacancies that effectively reduced NO_3_^−^ to NH_3_ in a 0.1 M NaOH solution containing 0.1 M NO_3_^−^, achieved a significant NH_3_ yield of 30.4 mg h^−1^ mg_cat_^−1^ and a FE of up to 92.6% [102].

CuCo_2_O_4_ possesses both tetrahedral (Td) and octahedral (Oh) structures, and doping atoms to replace Td or Oh sites can lead to an increase in oxygen vacancies (O_v_), enhancing the local electronic structure of these sites and providing more opportunities for regulating reaction intermediates. Moreover, the incorporation of dispersed single atoms not only modifies the spinel structure but also introduces active reaction centers. For instance, p-block metals such as Bi can effectively adjust the electronic structure of Cu and Co, optimizing the adsorption and desorption of reaction intermediates [39]. It is considered that enhancement is attributed to inhibitory effect of Bi on HER, which prevents *H coupling, resulting in more *H, as well as the strong interaction between Bi 6*p* orbitals and N 2*p* orbitals, which increases the adsorption capacity of nitrogen oxide reaction intermediates [29, 30].

Based on these, Lin et al. designed Bi-doped CuCo_2_O_4_ hollow carbon nanofibers to boost NH_3_ production from NO_3_RR [154]. The hollow structure could provide efficient active sites for the NO_3_RR, and the replacement Co sites with single Bi atoms in CoohO_6_ increased the O_v_ concentration and formed Bi–O–Co bonds, effectively optimizing the electronic properties of CuCo_2_O_4_ active sites. The DFT calculations demonstrated that Bi doping in CuCo_2_O_4_ could reduce the reaction barrier for *NO_2_ to *NO_2_H, while the energy barrier for the final step of *NH_3_ desorption also decreased from 1.44 to 1.2 eV, promoting the reactivation of surface-active sites. The maximum FE on NH_3_ production of Bi–CuCo_2_O_4_ reaches 95.53%, and a yield rate of 448.74 μmol h^−1^ cm^−2^ at − 0.8 V vs. RHE was recorded.

Li’s group prepared a catalyst that cobalt manganese spinel nanoparticles embedded in multichannel carbon fibers (CoMn_2_O_4_/NC), obtaining an optimal NH_3_ FE of 92.4% with a yield rate of 144.5 mmol h^−1^ g^−1^ at − 0.7 V vs. RHE [105]. Electrochemical in situ Raman spectra and online differential electrochemical mass spectrometry (DEMS) were used to identify the intermediates and products. These results, as well as the theoretical calculations, revealed that the interfacial between CoMn_2_O_4_ and NC induced the 3*d* orbital electrons of Co and Mn in less localized states, forming more positively charged regions, which promoted *NH_3_ desorption [105]. The theoretical calculation also demonstrated that the existence of NC could induce the electron delocalization of CoMn_2_O_4_, which significantly promoted NO_3_RR by reducing the ΔG of RDS (*NO to *NHO, 0.42 eV), and suppressed HER by enhancing the interaction of *H.

Inspired by the enhancement effect of oxygen vacancy for electrocatalysis, Wang et al. prepared a Ni_3_Co_6_S_8_ (NCS) catalyst and used a facile electrical joule heating method to flexibly regulate the sulfur vacancies (SVs) content on the surface of NCS, as shown in Fig. 9d [103]. The authors found that the NCS with reasonable SVs concentration (NCS-2) performed best and exhibited outstanding selectivity (98.2%), FE (85.3%) and yield rate (2388.4 µg h^−1^ cm^−2^) for electrocatalytic NO_3_RR to ammonia while maintaining their structural integrity. Combining the analysis of the Bader charge (Fig. 9e) and the adsorption energy of catalysts for NO_2_*, the SVs could increase the charge density of Co sites and enhance the adsorption of NO_2_*, reducing the energy barrier of the rate-determining step (HNO_3_* → NO_2_*), as well as suppressing the HER. Besides, the integrated-crystal orbital Hamilton population (ICOHP) value of Co–S bonds (− 2.17 eV) up to the Fermi level was weaker than that of Ni–S bonds (− 2.80 eV), as shown in Fig. 9f, suggesting Co–S bond interaction on the NCS was faintish and more prone to fracture; namely, the Co–S bonds were more likely to break than Ni–S bonds, making Co become the active site. In their another work, they ( Tao et al.) found that replacing the O ions in NiCo_2_O_4_ (NCO) by S ions to form NiCo_2_S_4_ (NCS) could improve the performance of the NO_3_RR to NH_3_. Evidence revealed that the bonding interactions among Ni–O or Co–O bonds are stronger than those among Ni–S and Co–S bonds. therefore, more 3d electrons can be localized at Ni or Co sites and further donate more charges to the intermediates. As a result, the ammonia yield was enhanced to 642 µg h^−1^ cm^−2^ and the selectivity could reach 92.42%, which was obviously more excellent than NiCo_2_O_4_ [106].

Apart from these pure Co-based compounds introduced above, the composition of these Co compounds is also an effective strategy to enhance the performance of electrocatalytic NO_3_RR. Ren et al. developed a catalyst of Ru-incorporated Co(OH)_2_ grown on nickel foam (CoRu–MOF/NF) as a cathode for NO_3_RR, which was in situ reconstructed from a Co-based metal–organic frameworks nanosheets pre-catalyst [107]. Interestingly, this pre-catalyst was also used as an anode that was in situ reconstructed to Ru–CoOOH for polyethylene terephthalate (PET) hydrolysate oxidation, realizing a co-electrolysis system for simultaneous upcycling of nitrate wastewater and PET plastic waste. This co-electrolysis system achieved a current density of 50 mA cm^−2^ at a cell voltage of only 1.53 V, realizing the simultaneous production of ammonia and formate at a lower energy consumption. The authors found that Ru incorporation could optimize the hydrogen adsorption–desorption properties of the Ru–Co(OH)_2_ electrode and supply more activated H species required for surface hydrogenation to proceed, thus facilitating the hydrogenation step for ammonia synthesis. And a high NH_3_ yield rate of 0.244 mmol h^−1^ cm^−2^ with a NH_3_ FE of 94.3% and NH_3_ selectivity of 98.57% were achieved at − 0.3 V vs. RHE. In addition, the Ru–Co(OH)_2_ electrode could still maintain a high catalytic activity in industrial-grade nitrate wastewater and ultrahigh nitrate concentrations, demonstrating the extensive nitrate concentration universality of Ru–Co(OH)_2_ for the NO_3_RR.

Recently, perovskite oxide has been developed as a new type of catalyst material due to its high catalytic activity, adjustable structure, and composition [108]. In particularly, perovskite oxides can allow their metal cations to exist in abnormal or mixed valence states, thereby resulting in enriched oxygen vacancies in their crystal structures [108]. As oxygen vacancies in oxide catalysts can adjust the adsorption energies of intermediates and affect their catalytic performance. In view of this, Zheng et al. sought to explore the catalytic performance of perovskite oxides for NO_3_RR to NH_3_ with respect to the amount of oxygen vacancies, where four perovskite oxides with different crystal structures (cubic LaCrO_3_, orthorhombic LaMnO_3_, and LaFeO_3_, hexagonal LaCoO_3_) were chosen and investigated [108]. Kinds of characterizations, such as X-ray photoelectron spectroscopy, electron paramagnetic resonance spectroscopy and electrochemical measurements, demonstrated that the amount of oxygen vacancies in these perovskite oxides surprisingly follows the same order as their activities toward NO_3_RR catalysis (LaCrO_3_ < LaMnO_3_ < LaFeO_3_ < LaCoO_3_). Notably, the LaCoO_3_ perovskite exhibited superior NO_3_RR activity and stability, with a high FE of 91.5% and an NH_3_ yield rate of 4.18 mmol mg^−1^ h^−1^. Theoretical studies revealed that the existence of oxygen vacancies in LaCoO_3_ perovskite can decrease the energy barriers for the reduction of *HNO_3_ to *NO_2_, thus leading to its superior NO_3_RR performance. This work showed the potential of Co-based perovskite toward electrocatalytic ammonia production, and in view of this, more efforts should be devoted to exploring this field.

#### Coordination with Phosphorus/Boron/Sulfur

**Fig. 10:**
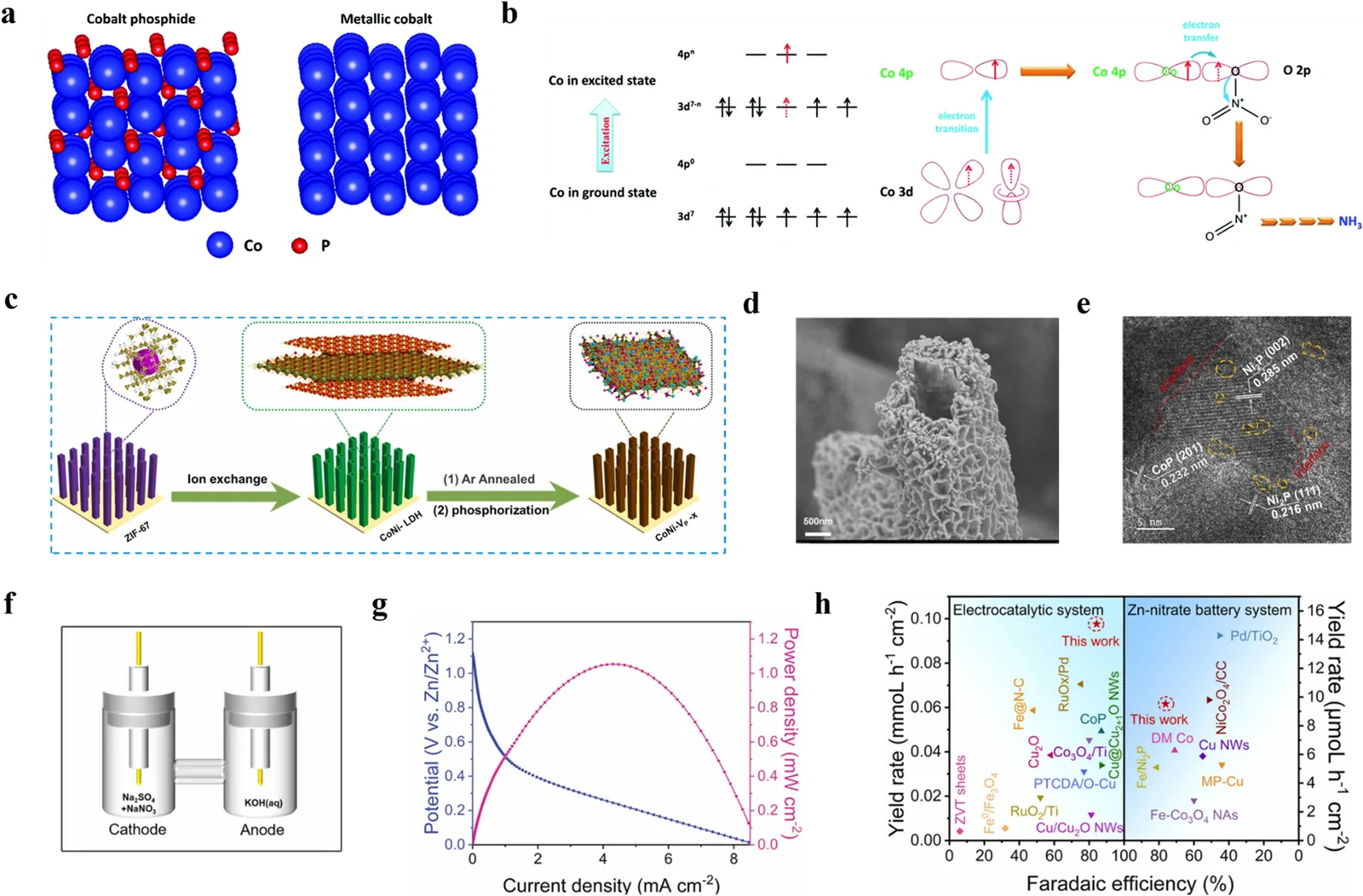
**a** Crystallographic structure of CoP and metallic Co [43]. Copyright 2022, Royal Society of Chemistry. **b** Mechanism of the NO_3_RR on CoP NAs/CFC [43]. Copyright 2022, Royal Society of Chemistry. **c** Schematic diagram for the synthesis of heterogeneous bimetallic phosphide CoP-Ni_2_P [122]. Copyright 2023, Elsevier. **d** SEM image of CoNi-Vp-1.0, where 1.0 represents the content of NaH_2_PO_2_·H_2_O of 1.0 [122]. Copyright 2023, Elsevier. **e** HRTEM image of CoNi-Vp-1.0 [122]. Copyright 2023, Elsevier. **f** Concept diagram of Zn-NO_3_^−^ battery [122]. Copyright 2023, Elsevier. **g** Discharging curves and the resultant power density curve of the CoNi-Vp-1.0-based Zn-NO_3_^−^ battery [122]. Copyright 2023, Elsevier. **h** FE and ammonium yield rate of CoNi-Vp-1.0 are compared with reported electrocatalytic NO_3_RR and Zn-NO_3_^−^-based catalysts [122]. Copyright 2023, Elsevier

Except for oxygen, phosphorus coordination is often used to modify the electronic structure and intrinsic properties for better NO_3_RR performance. Cobalt phosphides have a trigonal prism structure with chemical bonds ranging from metallic or covalent and metallic and semiconducting properties, giving rise to their high intrinsic activity and stability [109]. In detail, Co_2_P manifests a sophisticated crystal structure consisting of two cobalt atoms, and shows a relatively high surface area and abundant active sites due to the its intricate atom arrangement, which are pivotal for catalytic efficacy [110]. Regarding its electronic characteristics, Co_2_P may exhibit metallic or semimetallic behavior, characterized by mobile electrons within the conduction band [110]. CoP typically features a simpler crystal structure, but as a widely used catalyst, CoP demonstrates commendable catalytic activity and stability in terms of its electronic properties [110]. This MnP-type monophosphide CoP with metalloid atoms in metal lattice gaps, entail the interplay of cobalt and phosphorus atomic orbitals as well as electron filling and distribution, thereby influencing its band structure, conductivity, and shows high electrocatalytic NO_3_RR activity and practical potential []. For instance, A catalyst consisted of porous and amorphous cobalt phosphide nanoshuttles (CoP PANSs) were synthesized via precipitation transformation and high-temperature phosphidation [111]. Thanks to its porous structure, amorphous crystal structure and large surface area, the catalyst exhibited excellent electroactivity for NO_3_RR in neutral electrolyte. At − 0.5 V vs. RHE, CoP PANS achieved a high FE (94.24% ± 2.8%) and NH_3_ yield rate (19.28 ± 0.53 mg h^−1^). Additionally, Ye et al. designed cobalt phosphide nanosheet arrays grown on carbon fiber cloth (CoP NAs/CFC), which had an ammonia evolving rate of 9.56 mol h^−1^ m^−2^ at − 0.3 V vs. RHE with a FE of ∼100% in alkaline conditions [43]. The crystallographic structures of CoP and the corresponding metallic Co are shown in Fig. 10a, while Fig. 10b exhibits the mechanism of the NO_3_RR on CoP NAs/CFC. In this work, they systematic studied the reactive mechanism of NO_3_RR on CoP and metal Co; Operando X-ray absorption fine structure (XAFS) revealed that the electrons in the Co 3*d* orbitals were excited to 4*p* orbitals when NO_3_RR was triggered, and Co 4*p* orbitals may directly participated in the nitrate adsorption and electron transfer steps. In detail, the authors inferred that the excited electrons were transferred to the O 2*p* orbitals of nitrate via the Co–O–N covalent bond and injected into the π* orbitals of NO_3_^−^, then the adsorbed NO_3_^−^ was destabilized and eventually reduced. According to this, upshifting 3d orbitals to reduce the energy gap between 3*d* orbitals and 4*p* orbitals of metal sites might be an alternative to reduce the overpotential of NO_3_RR. Moreover, it is found that slight self-reconstruction at the surface of CoP (to form Co(OH)_2_) promoted water dissociation to release active hydrogen to hydrogenate the adsorbed NO_3_^−^, while severe self-reconstruction made it lose the catalytic activity. These results suggested that phosphorus has positive effects on catalytic activity and stability, and CoP nanostructure showed great potential for the application of NO_3_RR.

Cobalt-sulfur compounds are also widely considered to be potential electrocatalysts for NO_3_RR, although there is still a lack of research on the probable structural evolution, long-term stability, and reactive sites of cobalt-based sulfides during NO_3_RR. Guan et al. employed cobalt sulfides (Co_9_S_8_, CoS_2_, CoS_1.097_) as catalysts for electrocatalytic NO_3_RR under alkaline conditions, and found out that these cobalt sulfides were easily converted into cobalt hydroxide (Co(OH)_2_) during the NO_3_RR, which was seemingly contradictory to the thermodynamic prediction that these CoS_x_ compounds should be stable even under the catalytic condition [112]. Nevertheless, kinds of characterizations revealed that evolved Co(OH)_2_ species was proposed to be responsible for catalyzing NO_3_RR, especially during a long-term catalytic process. At − 0.8 V vs. RHE, all these CoS_x_ showed promising performances with FE of > 80% and a high yield of > 1780 mmol h^−1^ g_cat_^−1^ for NH_3_ production [112].

Beside these pure CoP nanostructures, compositing with other materials is also a feasible strategy [115–122]. For example, Deng et al. confirmed that the electrocatalytic NO_3_RR activity of CoP can be effectively enhanced by constructing p–n heterojunction of CoP/TiO_2_ nanoarrays on a titanium plate (CoP/TiO_2_@TP) [115]. The p–n hetero junction formed on the interface of CoP/TiO_2_ could establish a built-in electric field, which further accelerated the transport. As a result, this heterojunction structure attained an excellent FE of 95.0% and a high NH_3_ yield of 499.8 μmol h^−1^ cm^−2^, which was superior to CoP@TP and TiO_2_@TP. Theoretical calculations revealed that the p–n heterostructure could induce charge redistribution of CoP and TiO_2_, and optimize the adsorptive free energy of intermediates, in agreement with the experimental results.

Vacancy engineering, as a reasonable strategy to optimize the electronic structure and accelerate the kinetic process, can also be used on CoP to improve the performance toward electrocatalytic NO_3_RR. For instance, Gao et al. successfully synthesized heterogeneous bimetallic phosphide CoP-Ni_2_P with controllable phosphorus vacancy, as shown in Fig. 10c–e [122]. They found that the introduction of phosphorus vacancy promoted the charge accumulation of Co, Ni, and P atoms near the vacancy and modulated the electronic configuration so that the d-band center moved down from Fermi level. Besides, the phosphorus vacancy also enhanced the adsorption of NO_3_^−^, and could rarely realize the advance of the kinetic reaction decision step, making the potential determination step move from *NO + H_2_O + 2e −  → *N + 2OH^−^ to *NO_2_ + H_2_O + 2e^−^  → *NO + 2OH^−^ and the free energy of the potential determination step also decreased from 0.78 to 0.52 eV. Benefiting from other advantages such as high active area and superhydrophilicity, this heterogeneous bimetallic phosphide CoP-Ni_2_P nanotubes exhibited a FE of 84.27% for NH_4_^+^ synthesis and nearly 96% NO_3_^−^ conversion, with an excited NH_4_^+^ yield rate of 0.0977 mmol h^−1^ cm^−2^ at − 0.9 V vs. RHE in 50 ppm NO_3_^−^ and 0.5 M Na_2_SO_4_ aqueous solution. Meanwhile, a Zn-NO_3_^−^ battery with Zn foil as anode and CoNi-Vp as cathode also had a good energy supply and high ammonium yield, showing its potential for practical application in energy storage, as shown in (Fig. 10f–h).

Apart from CoP, Co_2_P also shows activity for electrocatalytic NO_3_RR. Yi et al. prepared high-quality Co_2_P nanodendrites by using a molten-salt-assisted synthesis method [116]. These Co_2_P nanodendrites exhibited an ammonia yield of 5.11 mg cm^−2^ h^−1^ at − 0.6 V vs. RHE, with an average FE of 88.57% and quite favorable stability. Similar to cobalt phosphides, Cobalt boride also shows activity for electrocatalytic NO_3_RR. Shi et al. synthesized uniformly dispersed amorphous CoB_x_ nanoparticles supported on carbon paper via a simple wet chemical reduction method^117^ . The CoB_x_ exhibited a maximum FE of 94.00% ± 1.67% and a yield rate of up to 0.787 ± 0.028 mmol h^−1^ cm^−2^ for ammonia production. The enhanced performance could be attributed to a partial electron transfer from B to Co, which was necessary for optimizing the adsorption energies of the reaction intermediates and facilitating electron transport [].

Phosphorus doping can also provide the coordination of P, for example, a cost-effective and stable three-dimensional phosphorus (P)-doped Co_3_O_4_/nickel foam (NF) was successfully prepared by Bi’s group [123]. The doping of P could replace the lattice oxygen in Co_3_O_4_ without changing the epitaxial nanowire morphology and could lead to enhancements in Co^3+^ percentage and empty d orbitals. As a result, the relative energy for the Volmer reaction and the adsorption energy of atomic H* decreased to − 0.73 and − 3.60 eV, respectively. In addition, P doping rendered Co_3_O_4_ with a higher electrochemical active area and a lower interface impedance. Benefiting from these, one as-prepared sample with a P element amount of 2.1% showed an increased NO_3_^−^ removal efficiency from 37 to 98% at the applied cathodic potential of − 1.3 V/SCE at pH 7.0 within 120 min. And this sample outperformed Co_3_O_4_/NF with an 8.45 times faster rate of NO_3_RR, in addition, the proportion of atomic H* to the NO_3_RR for this sample achieved 45%, which was 2.37 times higher than that of Co_3_O_4_/NF cathode (19%).

As for boron coordination, Xie et al. reported the in situ derivation of an amorphous Co_2_B layer on a Co_3_O_4_ nanosheet array on a Ti mesh (Co_2_B @Co_3_O_4_/TM) for efficient NO_3_RR under ambient conditions [34]. HRTEM (high-resolution transmission electron microscopy) images of the Co_2_B@Co_3_O_4_ nanosheet demonstrated that the surface of Co_3_O_4_ was covered by the Co_2_B layer. According to the fact that the NO_3_RR activity of the Co_3_O_4_/TM, Co_2_B/TM, and TM was much lower than that of the Co_2_B@Co_3_O_4_/TM, it could be inferred that Co_2_B in the Co_2_B@Co_3_O_4_/TM was the active site for NO_3_RR catalysis. Theoretical calculations also revealed that the Co_2_B layer was highly active for catalyzing the NO_3_RR with a free energy of 0.61 eV for the potential-determining step of *NO_2_H-to-*NO conversion. Even so, it can be seen that the Co_3_O_4_ also played a unique and indispensable role with regard to the high performance of Co_2_B@Co_3_O_4_/TM.

**Fig. 11:**
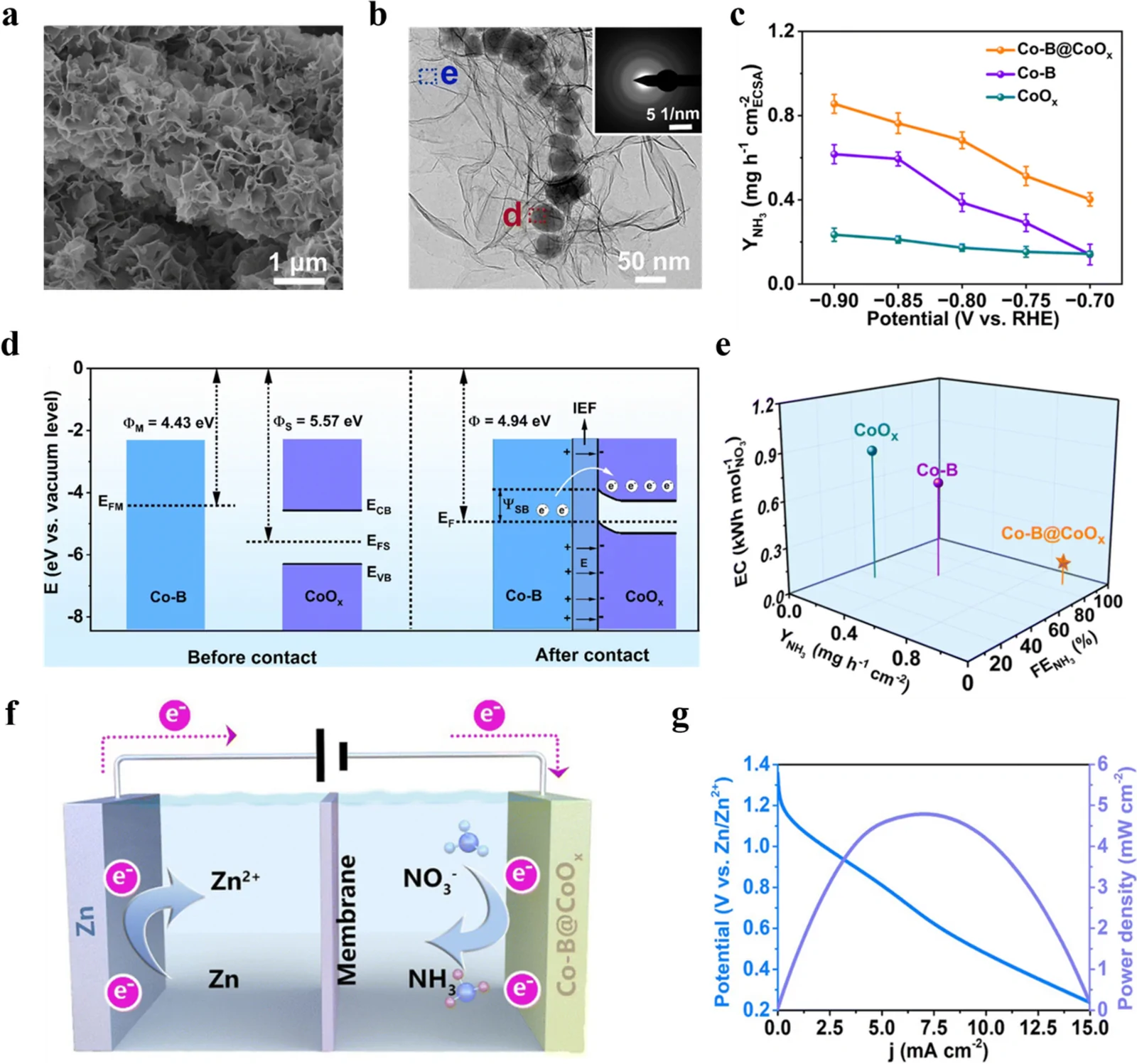
**a** SEM image of Co-B@CoO_x_ [124]. Copyright 2024, Royal Society of Chemistry. **b** TEM image of Co-B@CoO_x_ (the inset is the SAED pattern) [124]. Copyright 2024, Royal Society of Chemistry. **c** Normalized Y_NH3_ of Co-B@CoO_x_, Co-B, and CoO_x_ at different potentials [124]. Copyright 2024, Royal Society of Chemistry. **d** Energy band diagrams of metallic Co-B and p-type semiconductive CoO_x_ before and after contact. EFM = Fermi level of metallic Co–B, EFS = Fermi level of p-type semiconductive CoO_x_, IEF: internal electric field, CSB: Schottky barrier [124]. Copyright 2024, Royal Society of Chemistry. **e** Comparison of Y_NH3_, FE_NH3_, and EC over Co-B@CoO_x_, Co-B, and CoO_x_ at − 0.75 V [124]. Copyright 2024, Royal Society of Chemistry. **f** Schematic diagram of the Co-B@CoO_x_-based Zn-NO_3_^−^ battery [124]. Copyright 2024, Royal Society of Chemistry. **g** Discharging polarization curves and the corresponding power density of the Co-B@CoO_x_-based Zn-NO_3_^−^ battery [124]. Copyright 2024, Royal Society of Chemistry

Wang’ group designed a Mott–Schottky contact to synergistically boost the NO_3_RR under low-nitrate concentration, which was composed of an amorphous Co-B nanochain (cobalt borides) embedded in amorphous CoO_x_ nanosheets (Co-B@CoO_x_), as shown in Fig. 11a, b [124]. In 100 ppm NO_3_^−^, the Co-B@CoO_x_ catalyst exhibited remarkable performance, achieving over 95% NO_3_^−^ removal within 40 min at − 0.90 V vs. reversible hydrogen electrode and nearly 100% NH_3_ selectivity at − 0.80 V, surpassing the performance of both Co-B and CoO_x_ catalysts (Fig. 11c). Based on the relationship between the work function (WF) of metal and the energy band of the semiconductor, the energy band diagrams of metallic Co-B and p-type semiconductive CoO_x_ before and after contact are illustrated in Fig. 11d. Since the WF of metallic Co–B is lower than that of p-type semiconductive CoO_x_, charges at the interface undergo redistribution, with Co-B positively charged and CoO_x_ negatively charged. Such unidirectional transfer of electrons leads to the establishment of stable local electrophilic/nucleophilic regions, which could facilitate regulation of the adsorption behavior of reactant species. Furthermore, Co-B@CoO_x_ demonstrated an ultra-low energy consumption of 0.39 kW h mol_NO3_^−1^, establishing it as one of the most active catalysts available. Comprehensive experimental investigations and theoretical calculations indicated that the high conversion efficiency of Co-B@CoO_x_ originated from the formation of local nucleophilic/electrophilic regions at the Co-B/CoO_x_ Mott–Schottky interface, which effectively optimized the targeted adsorption behavior of NO_3_^−^ at the Co–B site and H_2_O at the CoO_x_ site, thereby enhancing simultaneously NO_3_^−^ affinity and active hydrogen availability. Furthermore, a novel Zn-NO_3_^−^ battery utilizing the Co-B@CoO_x_ catalyst delivered a remarkable power density of 4.78 mW cm^−2^, outperforming most recently reported Zn-NO_3_^−^ batteries (Fig. 11f, g).

### Single-Atom or Co-doped Single-Atom Catalyst

Single-atom catalysts (SAC) have emerged as a promising type of catalyst with much higher atomic utilization, distinct activity, and selectivity in comparison with bulk structures [94]. More importantly, their coordination environment is unique and can be easily regulated and controlled, showing great potential in effective nitrate-to-ammonia synthesis.

**Fig. 12:**
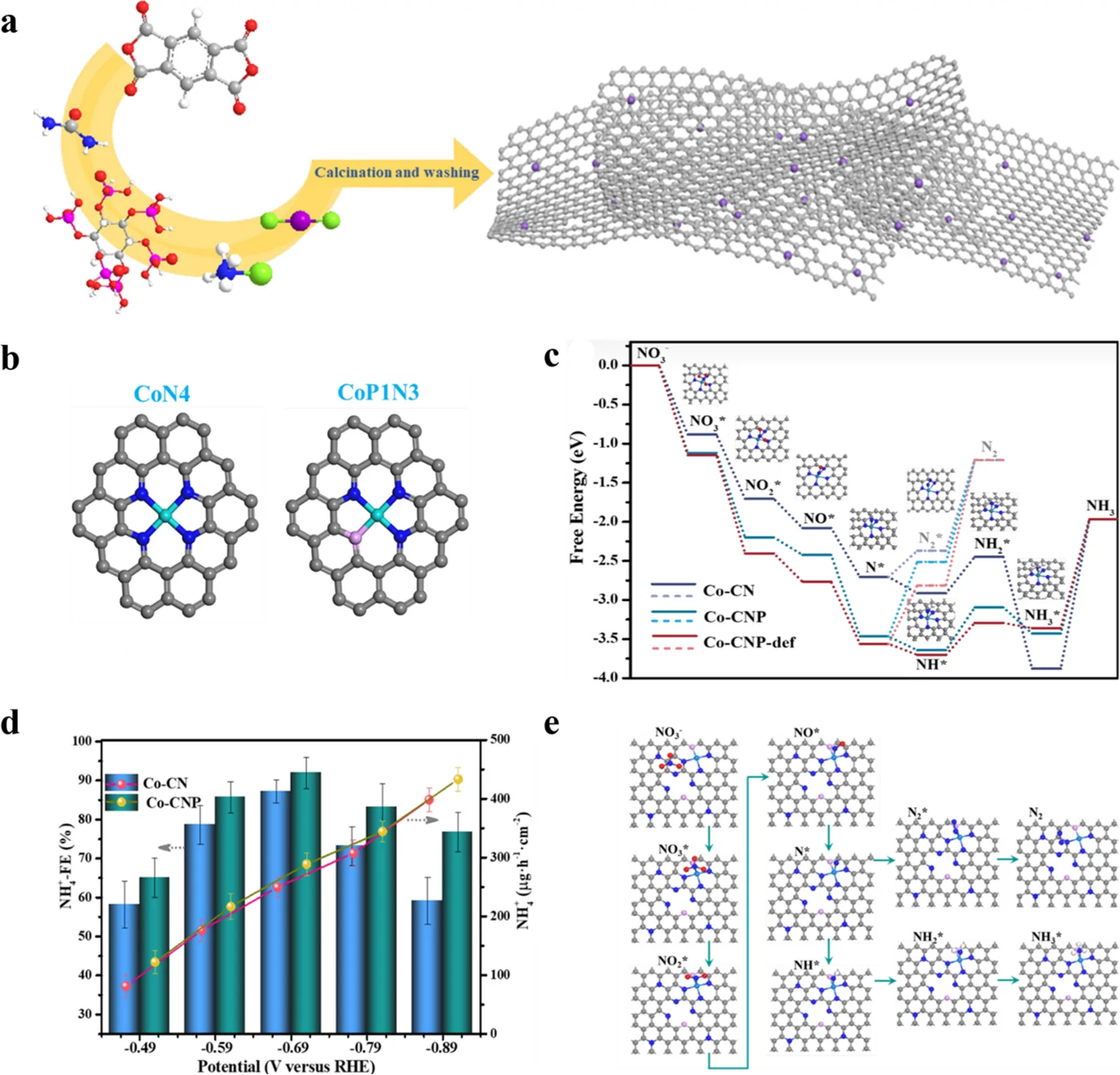
**a** Illustration of the preparation of Co SACs [119]. Copyright 2022, National Academy of Sciences. **b** Fitted configuration models for CoN_4_ and CoP_1_N_3_ in Co-CN and Co-CNP; the gray, blue, pink, and cyan balls refer to C, N, P, and Co atoms, respectively [119]. Copyright 2022, National Academy of Sciences. **c** DFT calculations: free energy changes for the elementary NO_3_RR steps on different SACs and adsorption models for different intermediates on the CoN_4_ site [119]. Copyright 2022, National Academy of Sciences. **d** NH_4_^+^ Faradic efficiency and NH_4_^+^ yield rate over Co-CN and Co-CNP at different potentials [119]. Copyright 2022, National Academy of Sciences. **e** Adsorption models on the CoP_1_N_3_ site affected by neighboring defect sites (The gray, blue, pink and cyan balls refer to C, N, P, and Co atoms, respectively [119]. Copyright 2022, National Academy of Sciences

Liu’s group designed Co-SACs supported on an N-doped carbon basal plane (Co–CN), and further modified with P to increase the number of defects and provide additional anchoring sites for single Co atoms (Co-CNP), resulting a strong metal-support interactions and CoP_1_N_3_ coordination structures (Fig. 12a, b) [119]. Both Co–N and Co–P bonds were formed in a CoP_1_N_3_ configuration, where Co was directly tetracoordinated with one P atom and three N atoms (Fig. 12b). In this configuration, the local environment of the Co atom was optimized by P-modification with asymmetric charge distribution and electron redistribution, which could not only exhibit better N=O activation performance than conventional N-cooperated single-atom sites, but also effectively inhibit the N–N coupling step, thus improving the selectivity toward NH_4_^+^ (Fig. 12c). As a result, a high Faradic efficiency of 92.0% and a maximum ammonia yield rate of 433.3 μg h^−1^ cm^−2^ were obtained (Fig. 12d). According to this interesting work, the strategy of heteroatom modification may significantly improve atom efficiency and further guide the future functional SACs design for NO_3_RR.

Besides this M–N–C structure, Co doping is often used to improve the performance of NO_3_RR due to the unique electronic structure of Co. Zhao et al. designed a highly active electrocatalyst with Co-doped TiO_2_ nanoribbon arrays supported on a Ti plate (Co–TiO_2_/TP)[120]. The Co doping TiO_2_ achieved a large NH_3_ yield of 1127 μmol h^−1^ cm^−2^ and high FE of 98.2% in 0.1 M NaOH with 0.1 M NO_3_^−^, much superior to its TiO_2_/TP counterpart (88.5 μmol h^−1^ cm^−2^; 35.1%). Theoretical calculations revealed that Co doping could effectively increase the content of oxygen vacancies in TiO_2_, thus enhancing the electrocatalytic performance of Co–TiO_2_/TP. Yu et al. designed and prepared mesoporous Co-doped Cu_2_(OH)_2_CO_3_ malachite nanosheets with oxygen vacancies via a facile hydrothermal synthesis method [31] . The authors found that the electrocatalytic performance was subjected to the Co/Cu ratio of this malachite, and the optimal electrocatalyst, 0.3Co@Cu_2_(OH)_2_CO_3_ (the molar ratio of Co and Cu was 1:3) displayed fast and highly efficient removal capacity of nitrate and high N_2_ selectivity. Above all, high total nitrogen removal efficiency (81.92%) and chemical oxygen demand (73.74%) in actual wastewater were also obtained. Density functional theory (DFT) calculations demonstrated that the introduction of Co into Cu_2_(OH)_2_CO_3_ weakened the activation energy of key reaction steps, thereby improving its NO_3_RR catalytic performance. This study discovered a new type of highly efficient electrocatalysts for applied to actual wastewater treatment to meet drinking water control standards.

As the electrochemical NO_3_RR catalyzed by single-atom catalysts (SACs) is an attractive and efficient way, more appropriate SACs, especially for M–N–C structures, should be explored to solve the problem of nitrate pollution in water and obtain valuable products such as ammonia. In addition, techniques traditionally employed to characterize ensemble materials are only able to provide limited information on SACs because of the less accessible active species, and the complexity of the NO_3_RR also leaves it a major challenge to obtain empirical evidence of the catalytic behavior of individual sites. Therefore, researchers should employ and explore more advanced characterization methods to address this challenge.

### Molecular Catalysts

Conventional phase-like catalysts such as metals, alloys and oxides, which are composed of closely packed atoms or ions, generally do not possess spatially separated active sites. While molecular catalysts are consisted of individual molecules, such as metal complexes, have clearly defined and active site, providing opportunity for detailed mechanistic insight [125]. Besides, their redox properties can be modified through ligand design, allowing for tunable electrochemistry, and finally control over reactant and product selectivities [126–129]. In particular, molecular electrocatalysts are also promising alternatives for the selective conversion of NO_3_^−^ to NH_3_ due to their well-defined structures, benefiting precise control of reaction pathways for desired products [128, 129]. The Co-based molecular catalysts such as cobalt phthalocyanine (CoPc) and cobalt porphyrin, have a similar coordination environment with Co single-atom catalyst, but with more molecular structures. Despite challenges including high cost, limited long-term stability, and potential toxicity that hinder practical implementation, molecular catalysts remain a research hotspot due to their unique advantages.

#### CoPc

Recently, metal phthalocyanines (MPcs) have received more and more attention in the field of catalysis due to their high catalytic activity, which is attributed to the central metal ions [129]. Metal phthalocyanines offer an ideal research model for NO_3_RR in light of the easy accessibility, chemical stability, and structural tunability of the unique conjugated 18π-electron skeleton [127, 129]. As one of the most widely used molecular electrocatalysts, cobalt phthalocyanine (CoPc) is used for different kinds of electrocatalytic reactions, such as CO_2_RR, NRR and NO_3_RR [127–135]. CoPc belongs to a kind of p-type semiconductor, and the centrosymmetric molecular structure endows it with mechanical and thermal stability, as well as electronic, catalytic, and semiconductor proprieties; besides, it can be anchored in cationic substrates by simple adsorption processes, which makes it easily be assembled with CoPc-based composites [127–129].

**Fig. 13:**
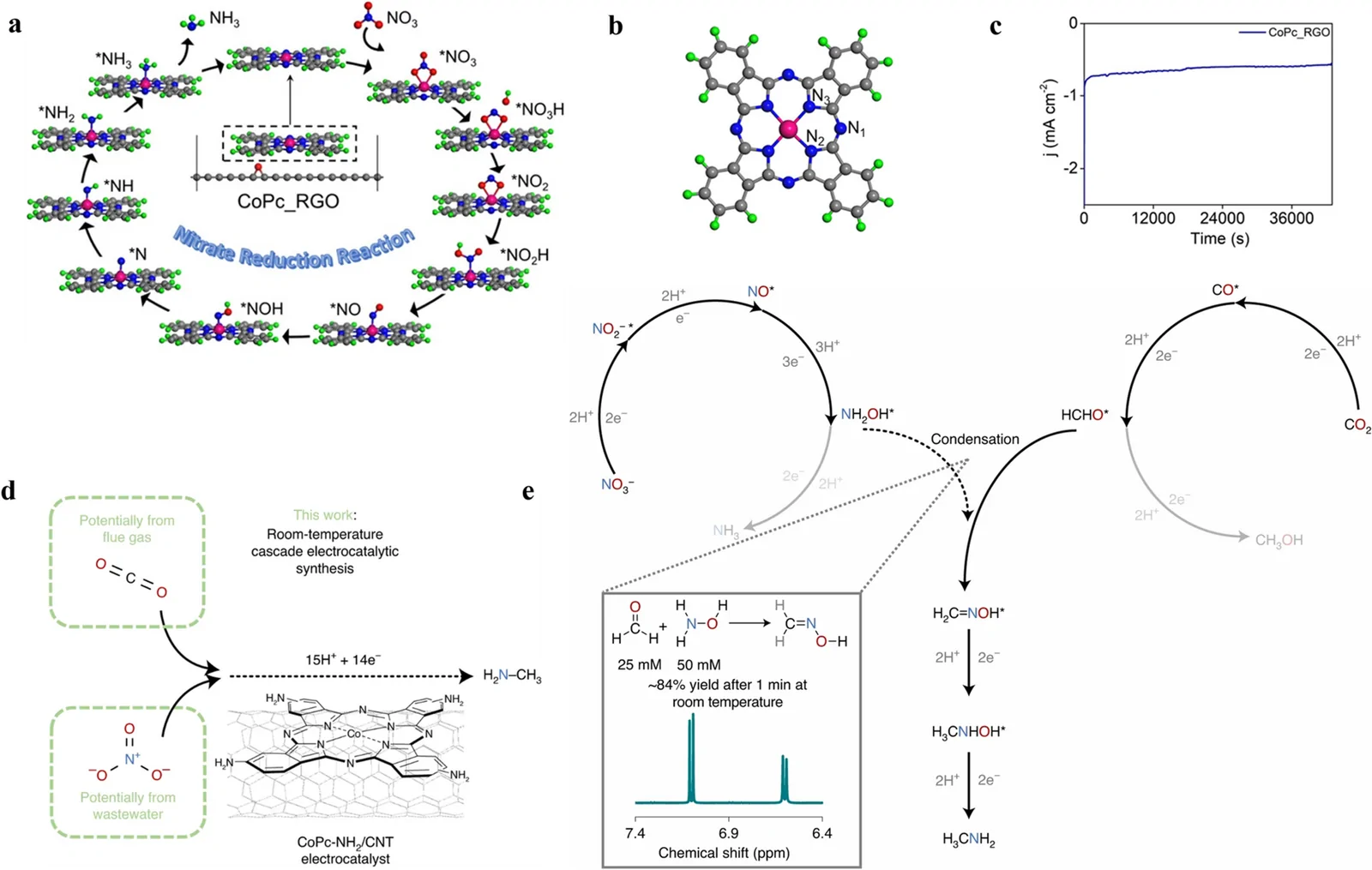
**a** Demonstration of the various steps of the NO_3_RR on the Co site of CoPc_RGO system (the symbol * denotes adsorption). The Co, N, C, O, and H atoms are denoted with pink, blue, gray, red, and green color spheres, respectively [121]. Copyright 2023, American Chemical Society. **b** The optimized structures of CoPc (Top view), the Co, N, C, and H atoms are denoted with pink, blue, gray, and green color sphere, respectively [121]. Copyright 2023, American Chemical Society. **c** 12 h long term chronoamperometry test for CoPc RGO toward NO_3_RR at − 0.2 V vs. RHE [121]. Copyright 2023, American Chemical Society. **d** One-pot electrosynthesis of methylamine from inorganic wastes at ambient temperature and pressure by CoPc-NH_2_/CNT catalyst [134]. Copyright 2021, Springer Nature. **e** Reaction intermediates of the C-N coupling process, which have been confirmed by experiments. The inset shows the fast kinetics and favorable thermodynamics of the C-N coupling step [134]. Copyright 2021, Springer Nature

Despite of the shortcomings of aggregation and hydrophobicity due to huge π-π-conjugated systems, the use of cobalt phthalocyanine (CoPc) has been favored over other metal phthalocyanine owing to its greater advantages such as high charge transfer capabilities, stability, high catalytic current density, reduced overpotential, diversify preparation methods, and high water-solubility. Paul et al. reported a CoPc-based electrocatalyst in which the CoPc nanotubes were anchored on reduced graphene oxide (RGO) 1D-2D heterostructures, and investigated the reaction route (Fig. 13a) [121]. The CoPc structure is shown in Fig. 13b, in which the Co atom is coordinated with four N atoms. This composite electrocatalyst (CoPc_RGO) exhibited an effective NO_3_RR performance with an NH_3_ yield rate and an FE of 58.82 μg h^−1^ mg_cat_^−1^ and 95.12% at − 0.2 V vs. RHE, as well as favorable stability, as shown in Fig. 13c. Bader charge investigation revealed the transport of charge to Co–N_4_ active sites from RGO, which aided the production of intermediates *NOH for NO_3_RR along with suppression of the parasitic HER, thereby demonstrating good selectivity and FE. In another case, Jiang et al. demonstrated that molecularly dispersed electrocatalysts (MDEs) of CoPc on carbon nanotubes could enable rapid and selective NH_3_ production from the NO_3_RR [126] . They found that these MDEs showed higher activity than the aggregative CoPc catalyst and showed higher activity and were more selective than the CuPc (copper phthalocyanine) MDE. The calculation results showed that the hybridization between NO_3_^−^ and the Co atom was stronger and the electron transfer was also enhanced, thus improving the favorable NO_3_^−^ absorption. Recently, an electrochemical reaction that converts nitrate (NO_3_^−^) and carbon dioxide (CO_2_) to nitrogen-containing compounds through a C-N coupling route has drawn great research interest [134]. This mild and energy-efficient approach provides an appealing alternative to the traditional energy-intensive N-containing compounds synthetic protocol, such as urea and methylamine. Wang’s group firstly reported that the co-electrochemical reduction of carbon dioxide and nitrate can produce methylamine (CH_3_NH_2_) in aqueous media under ambient conditions, which was catalyzed by a cobalt β-tetraaminophthalocyanine (CoPc) molecular catalyst supported on carbon nanotubes (CoPc-NH_2_/CNT) [134]. The overall reaction needed 14 electrons and 15 protons of transformation to form each methylamine molecule, involving an eight-step catalytic cascade process, as shown in Fig. 13d. The key C–N bond-forming step was found to be the spillover of hydroxylamine (NH_2_OH) from NO_3_RR and its subsequent condensation with formaldehyde (CH_2_O) from carbon dioxide reduction, as shown in Fig. 13e. This study provided a successful example of sustainable alkylamine synthesis from inorganic carbon and nitrogen wastes, which could guide future research to fully utilize the electrochemical NO_3_RR, and extend its reaction product beyond NH_3_ or N_2_. After this study, a phthalocyanine-based covalent organic framework (CoPc-COF) has also been successfully fabricated via a nucleophilic substitution reaction, which was grown on the surface of multilayered TiO_2_ nanotubes (CoPc-COF@TiO_2_ NTs) [118]. Differently, urea (CO(NH_2_)_2_) was produced from the co-reduction of CO_2_ and NO_3_^−^, and superior electrocatalytic activity was obtained, with a high urea yield of 1205 µg h^−1^ cm^−2^ and an outstanding FE of 49% at − 0.6 V vs. RHE. In situ attenuated total reflection infrared spectroscopic investigation and theoretical calculations unveiled the efficient C–N coupling reaction between *CO and *NH_2_ intermediates, which were mainly derived from CO_2_ on CoPc moieties and NO_3_^−^ on TiO_2_ NTs, respectively. Though the CoPc in this composite might not directly catalyzed the reduction reaction of NO_3_^−^, the synergistic effect between CoPc and TiO_2_ sites on this catalyst shows great potential for urea synthesis, which is helpful toward designing and fabricating high-performance electrocatalysts through the efficient synergistic effect of multiactive centers.

In situ attenuated total reflection infrared spectroscopic investigation and theoretical calculations unveiled the efficient C–N coupling reaction between *CO and *NH_2_ intermediates, which were mainly derived from CO_2_ on CoPc moieties and NO_3_^−^ on TiO_2_ NTs, respectively. Though the CoPc in this composite might not directly catalyzed the reduction reaction of NO_3_^−^, the synergistic effect between CoPc and TiO_2_ sites on this catalyst shows great potential for urea synthesis, which is helpful toward designing and fabricating high-performance electrocatalysts through the efficient synergistic effect of multiactive centers.

#### Co-porphyrins and Co-macrocycle

Other molecular electrocatalysts, such as metalloporphyrins and metal-cyclam have also been employed as catalysts for the electrochemical reduction of nitrate [136]. For instance, a series of simple molecular catalysts, such as Co(III), Fe(II), and Rh(II) protoporphyrins (metal-PP), directly adsorbed on pyrolytic graphite have been utilized for electrocatalytic NO_3_RR by coper’s group [136]. They found that among all investigated porphyrins, the Co-based protoporphyrin showed the highest selectivity toward hydroxylamine (NH_2_OH), and the reactivity and selectivity of the immobilized Co-protoporphyrin depended significantly on pH, with more acidic conditions leading to higher reactivity and higher selectivity toward hydroxylamine over ammonia. Besides, the potential also greatly influenced the selectivity: at pH 1, hydroxylamine was the main product around − 0.5 V with approximately 100% selectivity, while hydroxylamine and ammonia were both formed at a more negative potential, − 0.75 V. The mechanism of the reaction was also discussed, invoking the possibility of two pathways for hydroxylamine/ammonia formation: a sequential pathway in which hydroxylamine was produced as an intermediate, with ammonia subsequently formed through the reduction of NH_2_OH/NH_3_OH^+^, and a parallel pathway in which the formation of hydroxylamine and ammonia was derived from a common intermediate.

**Fig. 14:**
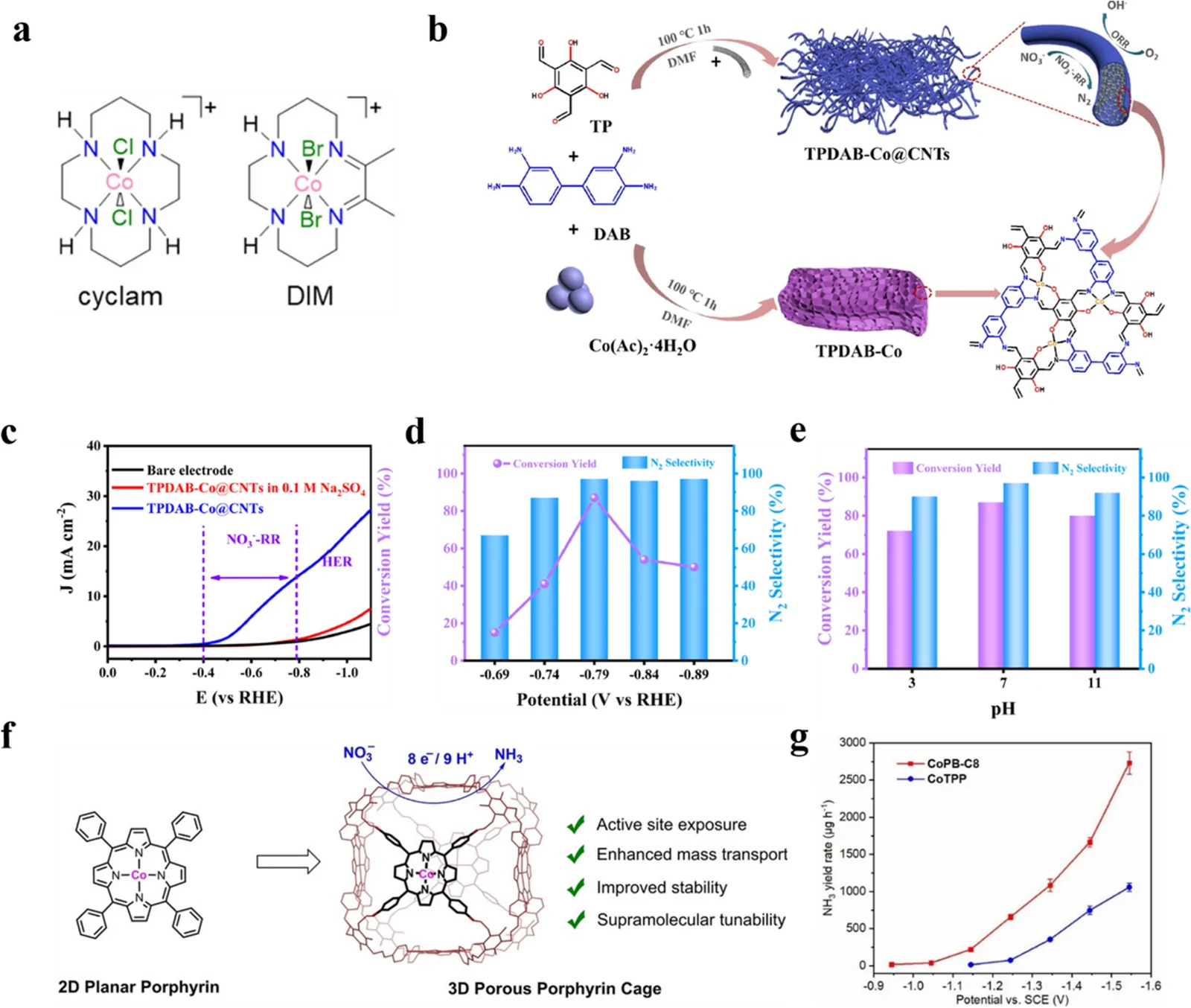
**a** Structure of cobalt macrocycle complexes [125]. Copyright 2022, American Chemical Society. **b** Schematic illustration of the synthesis procedures of TPDAB-Co and TPDAB-Co@CNTs [138]. Copyright 2020, Elsevier. **c** LSV patterns of TPDAB-Co@CNTs in the potential window of 0 to − 1.1 V vs RHE [138]. Copyright 2020, Elsevier. **d** Conversion yield and N_2_ selectivity of NO_3_RR on TPDAB-Co@CNTs under different electrolyzing voltages [138]. Copyright 2020, Elsevier. **e** Conversion yield and N_2_ selectivity of TPDAB-Co@CNTs with different pH of electrolytes [138]. Copyright 2020, Elsevier. **f** Supramolecular enhancement of electrochemical NO_3_RR catalyzed by embedding 2D molecular cobalt porphyrin into a 3D porous organic cage architecture [139]. Copyright 2023, Wiley–VCH. **g** NH_3_ yield rate for CoPB-C8 (red), and CoTPP (blue) [139]. Copyright 2023, Wiley–VCH

Recently, there has been significant interest in macrocycle complexes, and some of their structures are illustrated in Fig. 14a [125]. Xu et al. also reported that the cobalt macrocycle complex [Co(DIM)Br_2_]^+^ (DIM = 2,3-dimethyl-1,4,8,11-tetraazacyclotetradeca-1,3-diene) could be an effective electrocatalyst for the selective reduction of nitrate to ammonia in aqueous solution [137]. The catalyst could operate over a wide pH range with very high FE up to 97% for NH_3_ production, albeit with a large overpotential.

The mechanism for cleaving the N–O bond of nitrate has been investigated by experimental investigations as well as electronic structure calculations, which revealed that catalysis started when nitrate bound to the two-electron reduced species CoII (DIM), where cobalt and the macrocycle were each reduced by a single electron. The reduced DIM ligand played an important role in these mechanisms by directly transferring a single electron to the bound nitrate substrate, activating it for further reactions, and the DIM macrocycle was critical to NO_3_RR, specifically its combination of redox non-innocence, hydrogen-bonding functionality and flexibility in coordination mode. Similarly, Partovi et al. reported a cobalt complex, [Co(CR)Br_2_]^+^, where CR was the redox-active macrocycle 2,12-dimethyl-3,7,11,17-tetraazabicyclo-[11.3.1]-heptadeca-1(17),2,11,13,15-pentaene.

In 2020, Kwon et al. compared three potential macrocycle-based electrocatalysts for NO_3_RR: [Co(DIM)]_3_^+^, [Co(cyclam)]_3_^+^, and [Co(TIM)]_3_^+^, and found only [Co(DIM)]_3_^+^ was active for selective NO_3_RR to produce ammonia, even though these complexes have similar structures [140]. [Co(cyclam)]_3_^+^ could reduce aqueous nitrate to ammonia and hydroxylamine at heavy metal electrodes, while [Co(TIM)]_3_^+^ is inactive for the reduction of nitrate. DFT calculations revealed that [Co(DIM)]_3_^+^ showed the best performance due to a combination of less negative reduction potentials, which is attributed to the redox non-innocent ligand acting as an electron reservoir, and an accessible reaction barrier, stimulated by intramolecular hydrogen bonding. Liu et al. prepared a Co-salophen-enriched covalent organic frameworks (COFs) coating on carbon nanotube (CNT) wires, which were prepared with 1,3,5-triformylphloroglucinol (TP) and diaminobenzidine (DAB) by one-step Schiff-base type condensation reaction, as shown in Fig. 14b [138] . The Co-salophen COF@CNTs (TPDAB-Co@CNTs) exhibited large-pore mesoporous (4–60 nm) nanostructures with cotton-like morphology, in which the CNTs (~ 5 nm in diameter) were tightly coated by Co-salophen COFs. As a result, the porous polymer as the “linker agent” and CNTs as the “pillars” contributed to the spacious and porous composites, leading to an efficient electrocatalytic performance for reducing NO_3_^−^ to harmless N_2_, with a conversion yield of 87% and a N_2_ selectivity of 97%, as well as a wide available pH range and acceptable durability (Fig. 14c–e) [138].

In the field of molecular catalyst, supramolecular architectures recently have drowned much attention, as the molecular catalysts could be integrated into these discrete porous supramolecular architectures, such as organic cages, which represents an effective strategy to combine molecularly structural tailor ability with material porosity and stability and offers potential to bridge the gap between homogeneous and heterogeneous catalysis [139]. These supramolecular electrocatalysts retain the intrinsic reactivities of the molecular subunits, and usually augment their properties by embedding them within a confined space microenvironment with size-tunable cavities. It has been demonstrated that supramolecular systems are benefiting to NO_3_ recognition and transport, moreover, the porphyrin box organic cages are capable of serving as synthetic ion channels to transport nitrate anions across lipid bilayers [141, 142]. Based on these, An et al. integrated two-dimensional (2D) molecular cobalt porphyrin (CoTPP) catalyst units into a three-dimensional (3D) porous organic cage structure (CoPB-C8), and found that this hybrid bioinorganic structure markedly improved the catalysis’s activity and stability for electrochemical NO_3_RR, as shown in Fig. 14f, g [139] .

#### Polymers and Metal–organic Frameworks

**Fig. 15:**
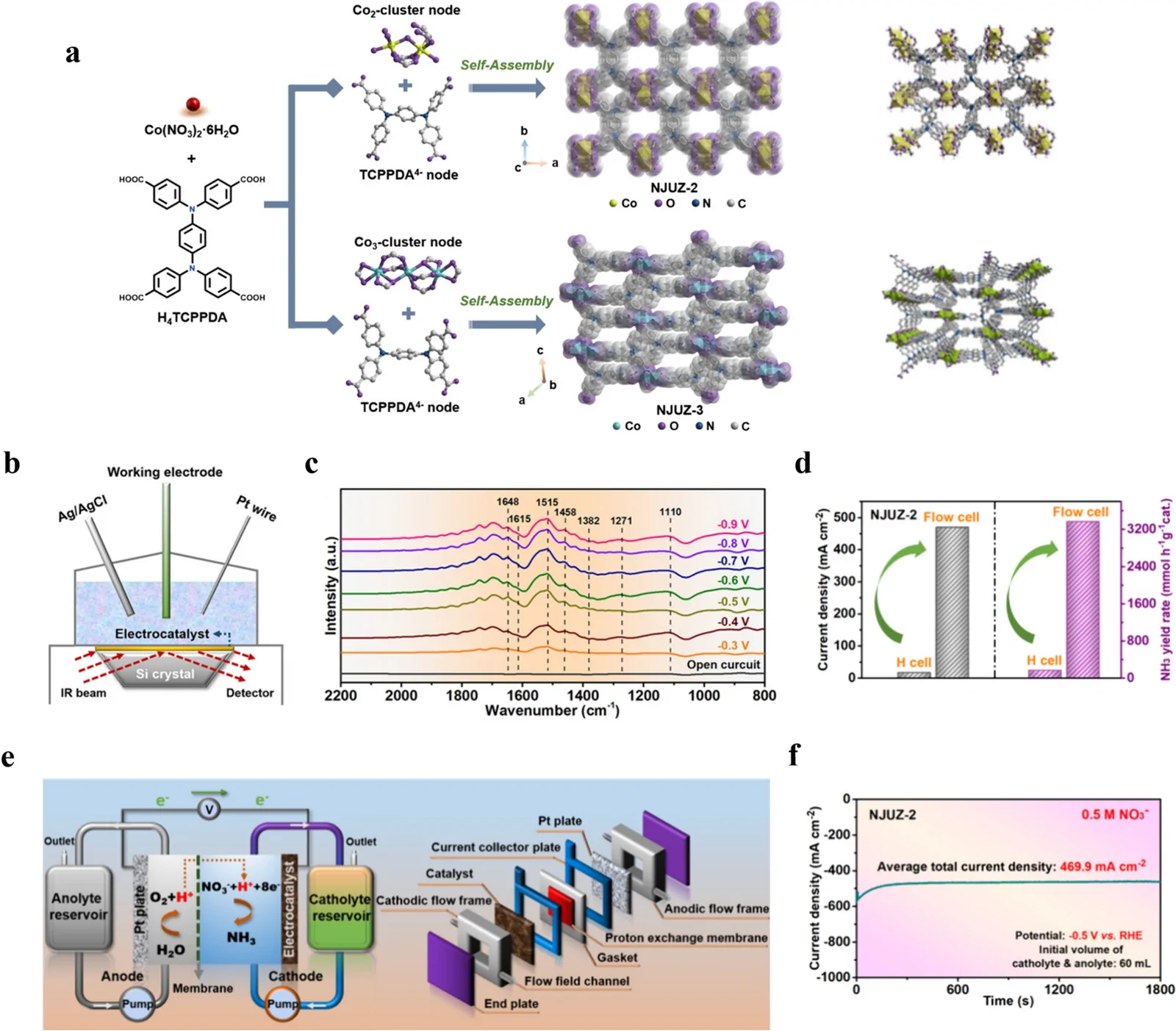
**a** Schematic structure assembly processes and crystal structures of (upper) NJUZ-2 and (bottom) NJUZ-3 CPs using organic − inorganic hybrid building blocks [35]. Copyright 2024, American Chemical Society. **b** Schematic illustration of the apparatus for in situ electrochemical ATR-FTIR characterizations [35]. Copyright 2024, American Chemical Society. **c** In situ electrochemical ATR-FTIR spectra of NJUZ-2 under different potentials, respectively [35]. Copyright 2024, American Chemical Society.** d** Comparisons of the as-measured current densities and NH_3_ yield rates measured in conventional H-type cell and flow cell under the same reaction conditions (0.5 M KNO_3_ electrolyte, − 0.5 V vs. RHE, 0.5 h reaction time) [35]. Copyright 2024, American Chemical Society. **e** Schematic illustration of the flow cell system [35]. Copyright 2024, American Chemical Society. **f** Time-dependent industrially relevant current density curve of the NJUZ-2 electrocatalyst at an applied potential of − 0.5 V vs. RHE [35]. Copyright 2024, American Chemical Society

As one kind of popular molecular catalytic materials, coordination polymers (CPs) and/or metal–organic frameworks (MOFs) have also emerged as promising catalytic materials for various electrocatalysis process [128–133]. Their distinct structural features, regular porosity, and tunable electron/ion transport characteristics originated from ligand design, offering CPs the advantages such as uniform well-defined scaffolds with multistage channels, enhancing mass diffusion, and allowing for mechanistic insight with atomic precision, as well as abundant catalytic sites, including explicit metal or cluster centers and functional organic linkers/groups. Wang et al. synthesized two distinct CPs, {[Co_2_(TCPPDA)(H_2_O)_5_]·(H_2_O)_9_(DMF)} and {Co_1.5_(TCPPDA)[(CH_3_)_2_NH_2_]·(H_2_O_6_(DMF)_2_}, designated as NJUZ-2 and NJUZ-3, as shown in Fig. 15a, where DMF denotes N, N-dimethylformamide [35]. These CPs were developed through the integration of polynuclear Co-based clusters as central nodes interconnected with the bridging H_4_TCPPDA ligands. Their electrocatalytic assessments for NO_3_RR demonstrated an impressive FE of ∼98.5% for NH_3_ production, surpassing previously reported catalysts.

Moreover, in situ ATR-FTIR (attenuated total reflectance Fourier transform infrared) spectroscopy was performed to gain insights into the intrinsic electrocatalysis mechanism during the NO_3_RR process; the schematic illustration of the apparatus and the results is shown in Fig. 15b, c. The peak at approximately 1458 cm^−1^ corresponds to the σ(N–H) bending mode of NH_4_^+^ species, suggesting significant hydrogenation leading to NH_3_ evolution. A negatively evolved band at 1382 cm^−1^ is attributed to the stretching mode of NO_3_^−^, indicating continuous consumption of NO_3_^−^. Several positive peaks concurrently emerged from various nitrogen-containing intermediates. The peak at 1271 cm^−1^ signified the N–O antisymmetric stretching vibration of NO_2_^−^, indicating the formation of the NO_2_^−^, intermediate through the NO_3_^−^, reduction. Another observed intermediate around 1110 cm^−1^ can be associated with the–N–O–stretching vibration of *ONH_2_, a crucial intermediate for NH_3_ formation. The positive peaks at 1515, 1615, and 1648 cm^−1^ correspond to the stretching mode of NO, representing the deoxygenation steps during NO_3_RR. As the development of a flow cell technique is essential to demonstrate a transition from benchtop-scale prototype to industrial-scale NO_3_RR technology deployment, the authors designed a custom-made flow cell system for conducting electrocatalytic tests, as displayed in Fig. 15d, e. In this system, an average total current density calculated to be 469.9 mA cm^−2^ could be obtained for the NJUZ-2 electrocatalyst (Fig. 15f), and a remarkable NH_3_ yield rate of 3370 mmol h^−1^ g_cat._^−1^ was achieved, marking a 20-fold increase when compared to that obtained in a conventional H-type cell reduction [35]. As a concise summary, Table 2 have gathered the electrochemical performance of some Co-based electrodes for NO_3_RR.

### Nanoclusters

In addition to what we have discussed above, atomic-scale nanoclusters also have attracted extensive attention due to their unique properties derived from their smaller size than conventional nanostructures, especially for atomically metal nanoclusters (MNCs, ≤ 2 nm) [143]. The ultra-small size endows these nanoclusters with high specific surface area, excellent atomic utilization efficiency and abundant active sites, therefore making them pioneer candidate materials in the field of electrocatalysis [144–147].

**Fig. 16:**
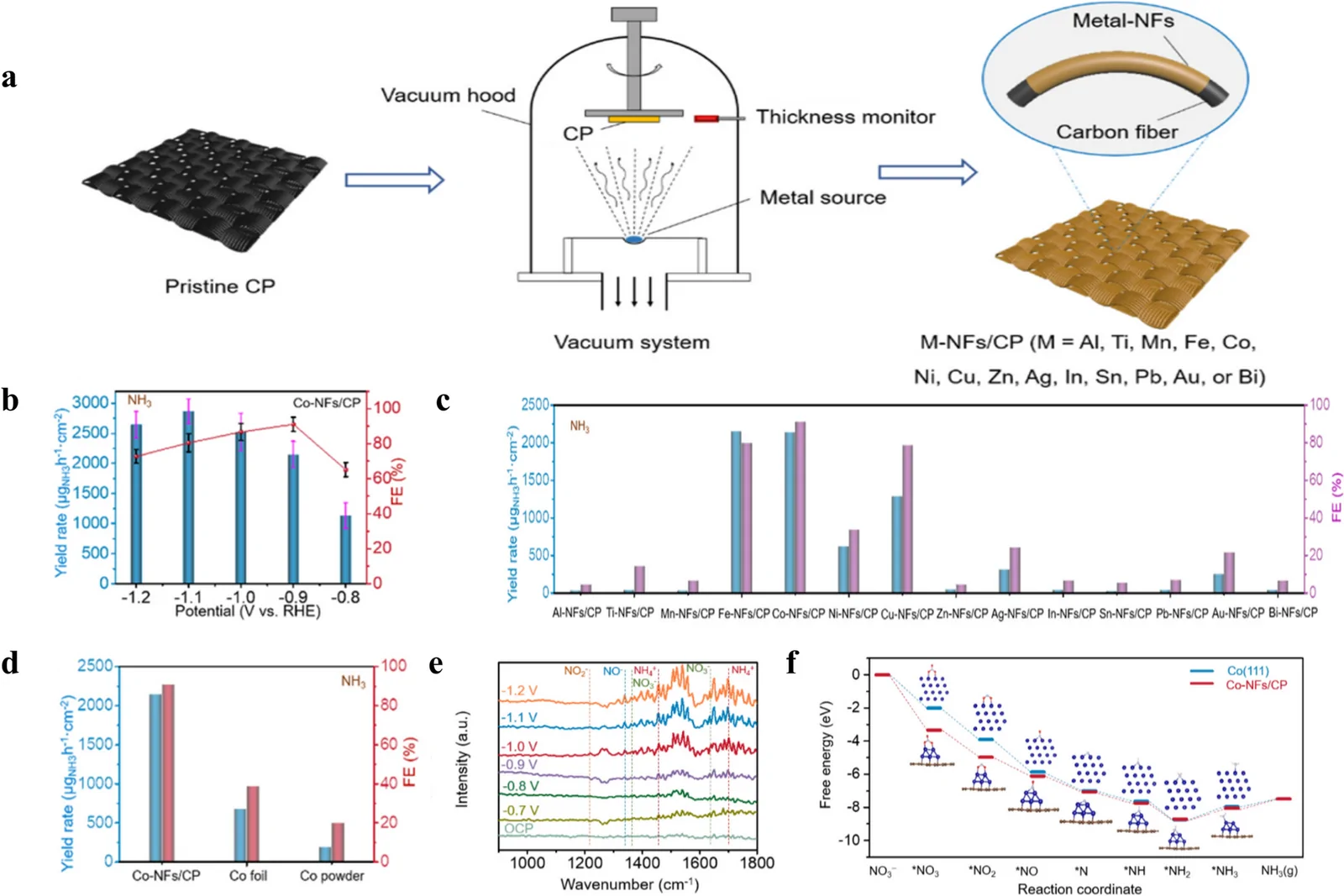
**a** Schematic diagram of different metal nanofilms deposited on CP by the thermal evaporation method [146]. Copyright 2024, Elsevier. **b** NH_3_ yields and corresponding FE values for Co-NFs/CP at given potentials for the NO_3_RR [146]. Copyright 2024, Elsevier. **c** Comparison of the NH_3_ yields and FE values of M-NFs/CP (M = Al, Ti, Mn, Fe, Co, Ni, Cu, Zn, Ag, In, Sn, Pb, Au, and Bi) samples at − 0.9 V vs. RHE [146]. Copyright 2024, Elsevier. **d** Comparison of the NH_3_ yields and FE values of Co-NFs/CP, commercial Co foil, and Co powder samples at − 0.9 V vs. RHE [146]. Copyright 2024, Elsevier. **e** In situ FTIR spectroscopy of Co-NFs/CP catalyst at different negative applied potentials containing 500 ppm NO_3_^−^ [146]. Copyright 2024, Elsevier. **f** Free energy diagrams of possible NO_3_RR pathways on Ti-COF Co-NFs/CP and Co (111) surface, respectively [146]. Copyright 2024, Elsevier

In recent years, some scholars have conducted some research on Co nanoclusters (Co NCs), and found them showed high NO_3_RR performance [146]. Jiang’s group reported the successful synthesis of 14 kinds of amorphous/low crystallinity metal nanofilms (such as Al, Ti, Mn, Fe, Co, Ni, Cu, Zn, Ag, In, Sn, Pb, Au or Bi) on the three-dimensional (3D) carbon fiber through a facile and rapid thermal evaporation method，as shown in Fig. 16 a. [146].

Compared with the other 14 amorphous/low-crystallinity mental nanofilms on carbon fibers, Co-NFs/CP was the optimal active metal materials for simultaneously enhancing NO_3_RR activity and selectivity in neutral electrolyte (Fig. 16b, c). Significantly, the NH_3_ FE of resulting Co-NFs/CP is 2.3- and 4.55-fold higher than those of commercial Co foil and Co powder (Fig. 16d), respectively, at − 0.9 V vs. RHE. The author detected the key intermediate and the possible reaction ways on the surface of the Co-NFs/CP during NO_3_RR by using in situ FTIR spectroscopy and DFT calculations, respectively (Fig. 16e, f). The appearance of signal peaks at approximately 1444, 1294, and 1116 cm^−1^ was indicative of the molecular adsorption of NH_3_ products. DFT calculations demonstrated that the amorphous Co structure on the Co-NFs/CP catalyst is conducive to enhancing the adsorption of *NO_3_ intermediates, while reducing the energy barrier required for the rate-determining step (*NH_2_ → *NH_3_), which is conducive to the occurrence of the NO_3_RR process. This study provides new insights into the systematic design of high-performance heterogeneous electrocatalysts based on amorphous or low crystallinity metals for NO_3_RR.

Beside these Co metal nanoclusters, Co oxides nanoclusters also have been developed for NO_3_RR. For example, Kani et al. synthesized a CoO nanoclusters/Graphene catalyst with a maximum NH_3_ FE of 98% at − 389 mA cm^−2^ and an NH_3_ yield of 25.63 mol g^−1^ h^−1^ [148]. DFT calculation showed that CoO NC/Graphene had similar activity as that of Co foil. In addition, due to the high price and scarcity of Co, this catalyst had the extremely low Co loading required for manufacturing nanoclusters, to a certain extent, achieved economic progress.

## Summary and Outlooks

In this review, we systematically analyze the electrocatalytic mechanisms, emphasizing the critical role of cobalt's electronic structure-governed by factors such as work function, hydrogen chemisorption energy, and d-band center position (E_d_ vs. E_F_)-in achieving high FE and ammonia selectivity. By tailoring coordination environments, dimensions, and compositions, the performance of Co-based catalysts can be significantly enhanced. We categorize and discuss various Co-based materials, including metallic cobalt, alloys, oxides, phosphides, borides, ternary compounds, single-atom catalysts, molecular catalysts, and nanocluster catalysts. Key findings reveal that catalytic activity is strongly influenced by crystallographic facets, nanostructure size, and morphology, as well as the coordination environment around Co sites, composition of the catalysts. Alloying strategies, particularly with metals like Fe, Cu, and Ru, effectively modulate electronic structures and improve conductivity. Cobalt oxides (e.g., Co_3_O_4_, CoO) and other compounds (e.g., CoP, CoB) demonstrate remarkable potential due to their tunable electronic properties. Single-atom and molecular catalysts further stand out for their high atom utilization efficiency and exceptional selectivity.

Among these Co-based materials, Co_3_O_4_ shows the advantages of low cost and the large reserves of raw material (the cost of industrial-grade cobalt oxide is approximately ￥160–170 per kilogram), as well as a good stability in alkaline environments. Moreover, It can be produced on a large scale through simple method, with controllable morphology (such as octahedrons, nanosheets), and is suitable for electrode preparation. However, the intrinsic semiconductor properties result in low electron conduction efficiency, and need compensatory conductive carriers (such as graphene, carbon nanotubes) to be provided. During the catalytic process, surface reconstruction (such as Co^3+^  → CoOOH) is prone to occur, and the activity may decay severely after long-term operation. In addition, under the actual wastewater, Cl⁻ will form [CoCl₄]^2−^ complexes with Co, competing for active sites and reducing the catalytic efficiency. While for CoP/Co_2_P, they show an high corrosion resistance, especially in acidic electrolyte. However, the syntheses of cobalt phosphides are often complex and even with high risk. As for alloys, the introduction of noble metal such as RuCo, AuCo alloy may highly improve the performance of NO_3_RR, especially in the applicable supply of active hydrogen species. while the high cost is a concern for industrial application, resulting in poor economic performance.

**Fig. 17:**
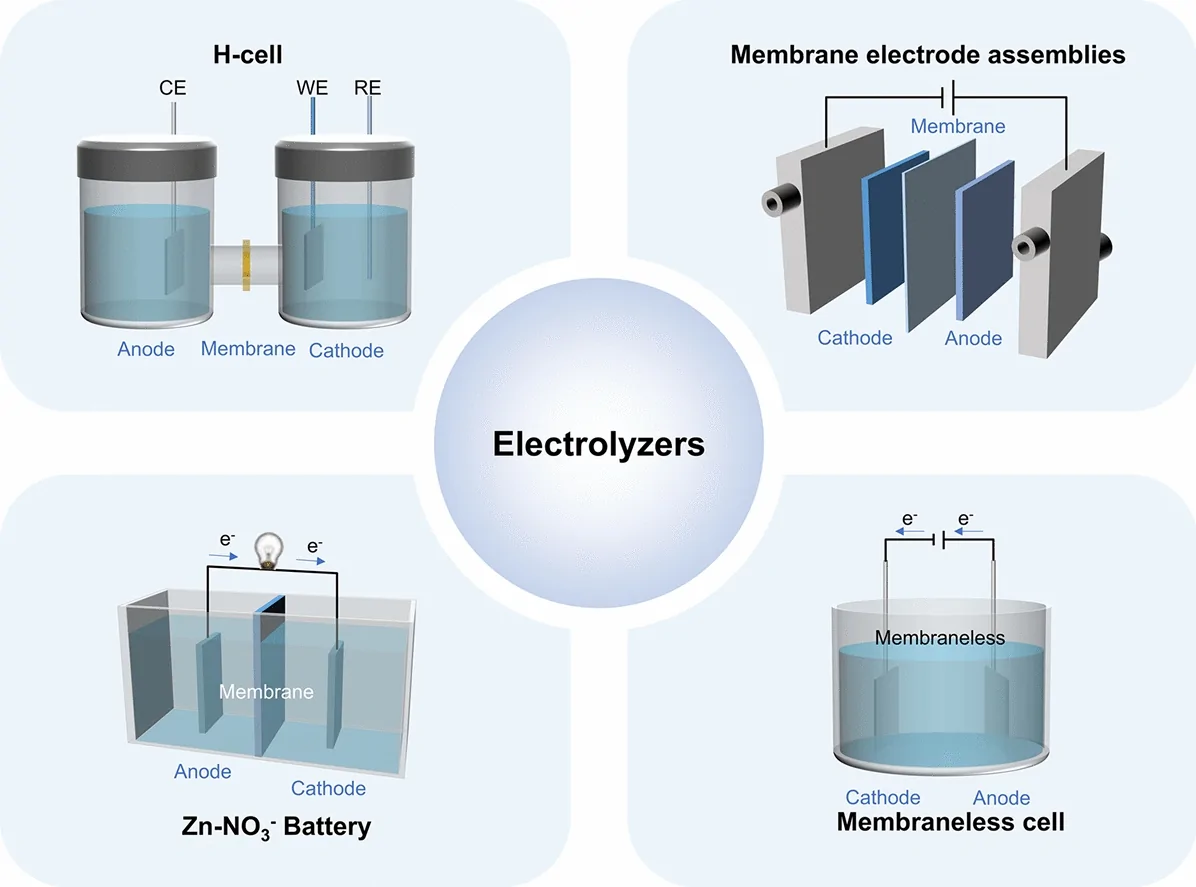
The schematic diagram of configurations for H-cell, MEA, Zn-NO_3_^−^ battery, and membraneless reactor

As for future industrial application for NO_3_RR, the appropriate catalysts with low overpotential (high energy efficiency) and industrial-grade current density are necessary and thus can be expected to become the preferred alternative to the traditional Haber–Bosch process for ammonia synthesis. On the other hand, as the core of NO_3_RR research involves around two major pillars: catalyst innovation and electrolyzer design, complementing these advancements in catalysts with qualified electrolyzer configurations is also necessary for industrial application. H-cell configurations are sample and easy to be carried out, having been instrumental in elucidating reaction mechanisms and evaluating catalyst performance. Although it is notable that only a few studies have employed membrane electrode assemblies (MEA), these MEA-based systems are essential for scaling up NO_3_RR technologies from laboratory-scale research to industrial applications. MEA technology represents a significant advancement in electrolyzer design and can enhance operational stability by facilitating efficient proton transport while minimizing side reactions such as hydrogen evolution. In addition, Zn–NO_3_^−^ batteries device offers the possibility of combining the two functions of NH_3_ synthesis and power supply. While membrane-free reactors have expanded the operational flexibility of NO_3_RR technologies, with high system stability, product selectivity, and low cost (due to the high price of membrane). The configurations of these electrolyzers are shown in Fig. 17. Since the NO_3_RR occurs in a liquid system, the obtained ammonia actually exists in the aqueous solution in the form of NH_4_^+^, which is still a pollutant from the perspective of environmental governance. Therefore, only if one could separate ammonia products from the original system can wastewater denitrification finally be achieved.

In conclusion, despite the advancements, challenges remain: (1) sluggish kinetics due to weak NO_3_^−^ adsorption and complex proton/electron transfer steps; (2) scaling relations between intermediate binding energies; (3) limited product diversity (primarily N_2_ and NH_3_); and (4) insufficient durability under operational conditions. (5) insufficient research attention oriented toward industrial application. To address these issues, future research should focus on the following key directions:

1. Advanced catalyst design: Develop hierarchical nanostructures and tailored coordination environments to optimize active site accessibility, charge transfer, and mass transport, while minimizing byproduct formation and enhancing NH_3_ selectivity. Expand the study of underutilized cobalt oxides (e.g., CoO, Co_2_O_3_) and multicomponent alloys to uncover new catalytic synergies and improve performance. Investigate dynamic structural transformations or hybridization with single-atom catalysts and molecular catalyst systems to create multifunctional catalytic platforms. Implement high-throughput screening and machine learning approaches for discovering cobalt-based catalysts with optimal characteristics.
2. Durability enhancement: Improve catalyst robustness through surface engineering, dynamic operational strategies (e.g., pulsed electrolysis), and optimized intermediate desorption properties. Exploit strategies for improving stability under high current densities or during extended nitrate electrolysis.
3. Product diversification: Design catalysts capable of producing high-value nitrogen-containing compounds (e.g., urea, methylamine) by incorporating diverse carbon sources and exploring novel reaction pathways.
4. Mechanism analysis: Utilize operando techniques and machine learning approaches to monitor structural changes during catalysis, obtaining a better understanding of the fundamental mechanism including the reaction pathway, catalyst deterioration, and so on.
5. Developing scalable and uniform synthesis methods for cobalt-based catalysts.
6. Real-World Application: Address challenges related to real-world applications in complex wastewater matrices, including the impact of water contaminants (e.g., Ca^2+^, Mg^2+^, organic matter) on NO_3_RR performance, electrochemical devices design and fabrication for industrialized NO_3_RR under real-world wastewater condition.

**Fig. 18:**
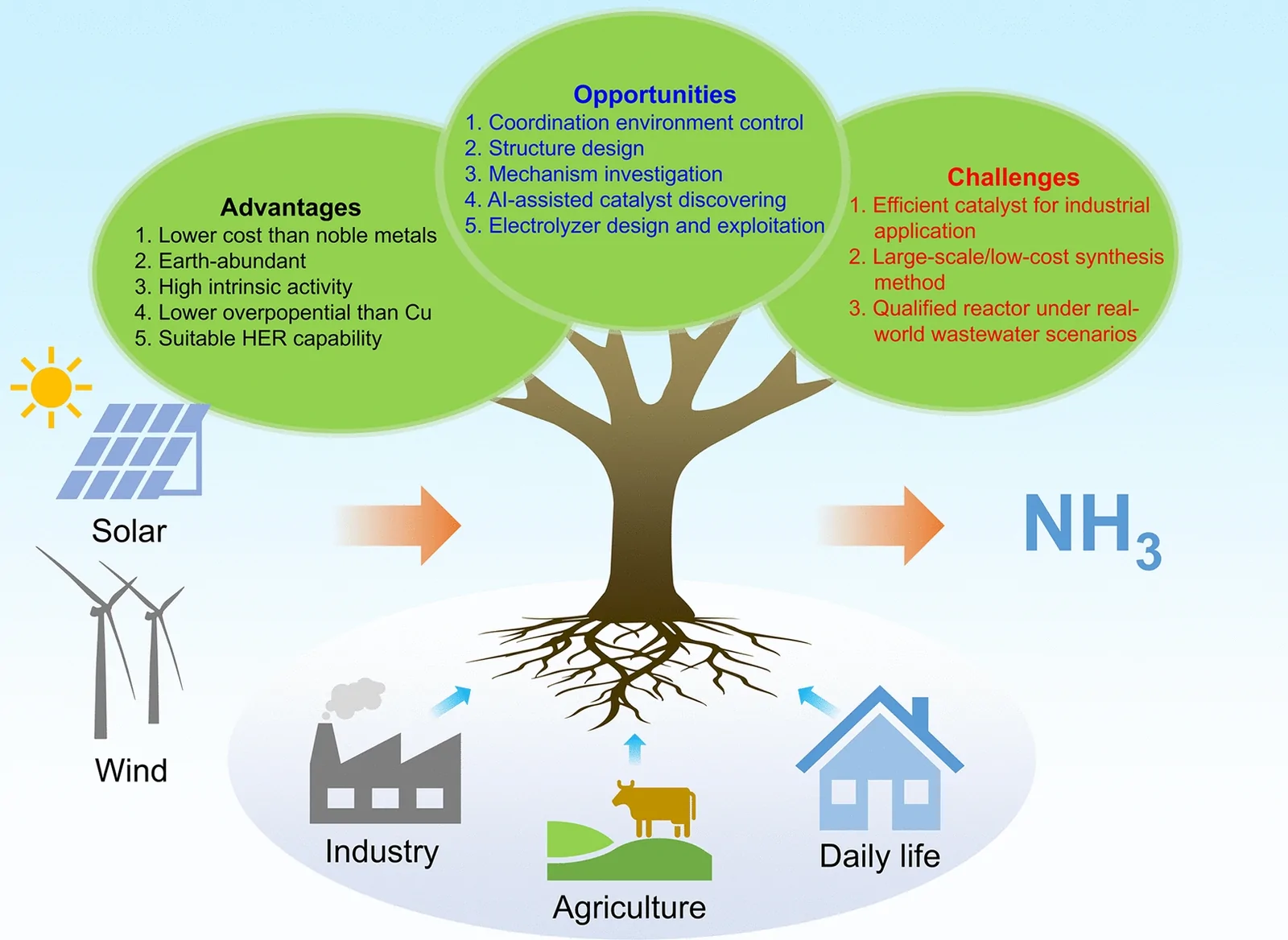
Schematic diagram of the advantages, opportunities, and challenges of Co-based catalysts for NO_3_RR

**Fig. 19:**
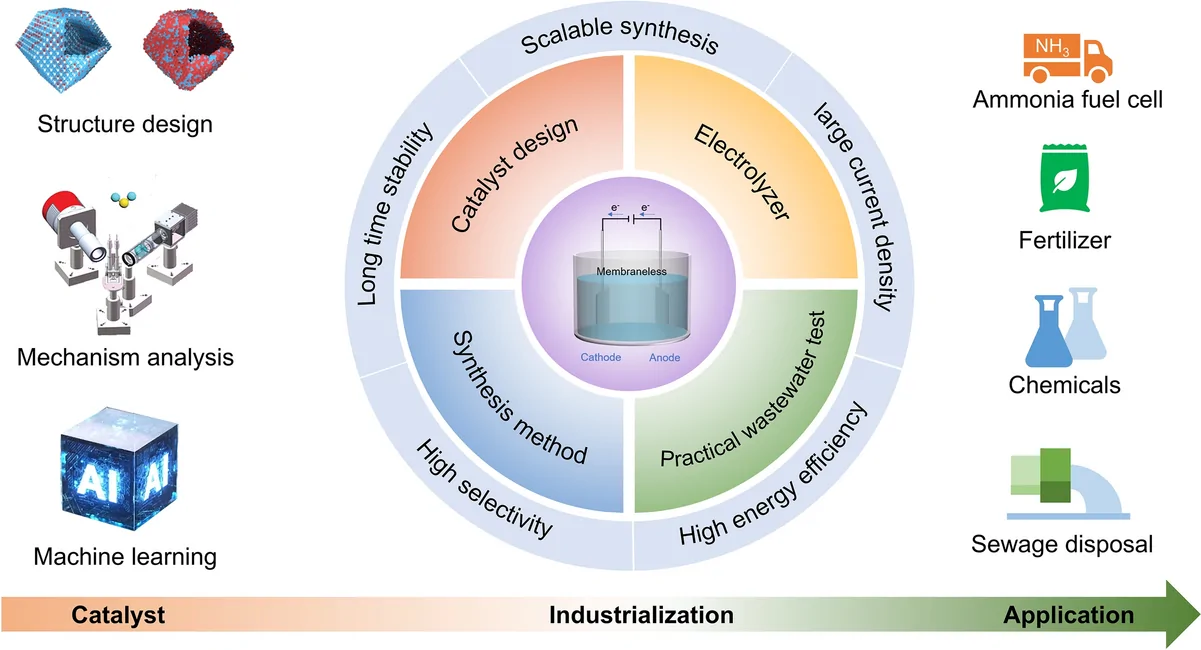
Schematic diagram of the fundamental research, industrialization, and application of NO_3_RR

The advantages, opportunities, and challenges of Co-based catalysts for NO_3_RR are summarized in Fig. 18. Moreover, a schematic diagram including the catalysts design and synthesis, electrolyzer innovation, industrialization of NO_3_RR, as well as the future application of these advances, is shown in Fig. 19. By addressing these challenges and pursuing these research directions, Co-based electrocatalysts can advance toward practical applications, enabling sustainable nitrate conversion and contributing to the development of green nitrogen chemistry.
